# Construction and analysis of mRNA, miRNA, lncRNA, and TF regulatory networks reveal the key genes associated with prostate cancer

**DOI:** 10.1371/journal.pone.0198055

**Published:** 2018-08-23

**Authors:** Yun Ye, Su-Liang Li, Sheng-Yu Wang

**Affiliations:** 1 Department of Clinical Laboratory, The First Affiliated Hospital of Xi'an Medical University, Xi'an, Shaanxi, China; 2 Department of Respiration, The First Affiliated Hospital of Xi’an Medical University, Xi’an, Shaanxi, China; University of São Paulo, BRAZIL

## Abstract

**Purpose:**

Prostate cancer (PCa) causes a common male urinary system malignant tumour, and the molecular mechanisms of PCa are related to the abnormal regulation of various signalling pathways. An increasing number of studies have suggested that mRNAs, miRNAs, lncRNAs, and TFs could play important roles in various biological processes that are associated with cancer pathogenesis. This study aims to reveal functional genes and investigate the underlying molecular mechanisms of PCa with bioinformatics.

**Methods:**

Original gene expression profiles were obtained from the GSE64318 and GSE46602 datasets in the Gene Expression Omnibus (GEO). We conducted differential screens of the expression of genes (DEGs) between two groups using the online tool GEO2R based on the R software limma package. Interactions between differentially expressed miRNAs, mRNAs and lncRNAs were predicted and merged with the target genes. Co-expression of miRNAs, lncRNAs and mRNAs was selected to construct mRNA-miRNA-lncRNA interaction networks. Gene Ontology (GO) and Kyoto Encyclopedia of Genes and Genomes (KEGG) pathway enrichment analyses were performed for the DEGs. Protein-protein interaction (PPI) networks were constructed, and transcription factors were annotated. Expression of hub genes in the TCGA datasets was verified to improve the reliability of our analysis.

**Results:**

The results demonstrate that 60 miRNAs, 1578 mRNAs and 61 lncRNAs were differentially expressed in PCa. The mRNA-miRNA-lncRNA networks were composed of 5 miRNA nodes, 13 lncRNA nodes, and 45 mRNA nodes. The DEGs were mainly enriched in the nuclei and cytoplasm and were involved in the regulation of transcription, related to sequence-specific DNA binding, and participated in the regulation of the PI3K-Akt signalling pathway. These pathways are related to cancer and focal adhesion signalling pathways. Furthermore, we found that 5 miRNAs, 6 lncRNAs, 6 mRNAs and 2 TFs play important regulatory roles in the interaction network. The expression levels of EGFR, VEGFA, PIK3R1, DLG4, TGFBR1 and KIT were significantly different between PCa and normal prostate tissue.

**Conclusion:**

Based on the current study, large-scale effects of interrelated mRNAs, miRNAs, lncRNAs, and TFs established a new prostate cancer network. In addition, we conducted functional module analysis within the network. In conclusion, this study provides new insight for exploration of the molecular mechanisms of PCa and valuable clues for further research into the process of tumourigenesis and its development in PCa.

## Introduction

Prostate cancer (PCa) involves a common male urinary system malignant tumour that has the highest incidence among European and American populations [1,2]. This disease not only seriously affects the quality of life of patients but is also associated with financial burdens for society and the family [3,4]. PCa is closely regulated by various cytokines, related genes and intercellular signalling networks in the course of its development. However, the specific molecular mechanisms remain unclear. Therefore, it is important to study the molecular mechanisms of prostate cancer and to prevent the disease and develop effective treatments.

miRNAs are a class of non-coding small RNAs that can be combined with the 3-'UTR of target mRNA to regulate gene expression, which leads to abnormal expression of target genes. A large number of studies have reported abnormal expression of miRNAs in prostate cancer involving multiple miRNAs, such as miR-16, 221, 375, 1290 and 141 [5–7]. Abnormal miRNA expression has been confirmed to be closely related with long non-coding RNAs (lncRNAs) and transcription factors (TFs). lncRNAs regulate miRNA expression by binding and sequestering target miRNAs and participating in mRNA expression regulation [8,9]. lncRNAs are emerging as novel diagnostic markers of PCa, and Chang et al. found that HOTAIR may participate in PCa progression[10]. Tian et al. revealed that RNCR3 functions as a tumour-promoting lncRNA in prostate cancer[11]. Li et al. found long noncoding RNA BDNF-AS is associated with clinical outcomes and plays a functional role in human prostate cancer[12]. As important factors in gene transcription and post-transcriptional regulation, TFs are also involved in controlling signaling pathways with miRNAs [13,14]. A previous study found that CREB1 and FoxA1 associated with advanced prostate cancer, and CREB1/FoxA1 target gene panels can predict prostate cancer recurrence[15]. However, further research is needed to determine the regulatory mechanisms of miRNAs, lncRNAs, TFs and mRNAs in PCa.

Microarray analysis can quickly identify all genes that are expressed at the same time-point [16]. Future research could benefit from integration and analysis of these data [17]. In this work, we identified differentially expressed genes (DEGs) in prostate cancer from the GSE64318 [18] and GSE46602 [19] datasets. We performed Gene Ontology and Kyoto Encyclopedia of Genes and Genomes pathway analysis for significant DEGs. Furthermore, we analysed mRNA-miRNA-lncRNA and protein-protein interaction (PPI) networks to reveal interactions and identified some factors that may be associated with PCa regulatory mechanisms. Finally, we analysed hub genes based on the PPI network and TCGA datasets. This study will contribute to understanding the molecular mechanism of prostate cancer, providing valuable clues for further research.

## Material and methods

### Raw data

The datasets used in the present study were downloaded from the National Center of Biotechnology Information (NCBI) Gene Expression Omnibus (GEO) (https://www.ncbi.nlm.nih.gov/geo/) [20]. The experimental articles that were used to compare the RNAs in prostate cancer tissue and noncancerous prostate tissue from patients were included. The original gene expression profiles were obtained from the GSE64318 [18] and GSE46602 datasets [19] (S1 and S2 Tables). The GSE64318 dataset includes microRNA expression profiles of prostate biopsy samples from 54 prostate biopsy specimens (27 tumours and 27 normal tissues). The platform used for these data is the GPL8227 Agilent-019118 Human miRNA Microarray 2.0 G4470B (miRNA ID version). The GSE46602 dataset includes genome expression profiling of tumour tissue specimens from 36 patients with prostate cancer and normal prostate biopsies from 14 patients. The platform used to analyse these data was the GPL570 Affymetrix Human Genome U133 Plus 2.0 Array.

### Identification of differentially expressed genes

GEO2R (http://www.ncbi.nlm.nih.gov/geo/geo2r/) is an interactive web tool based on the R language limma package [21] that can be used to compare two or more groups of samples to identify differential expression in a GEO series. In the present study, GEO2R was used to filter differentially expressed mRNAs and miRNAs between normal and tumour samples separately in each of the datasets. The false discovery rate (FDR) is a method of conceptualizing the rate of type I errors in null hypothesis testing when conducting multiple comparisons. GEO2R calculates the FDR automatically. The multiple t test was used to detect statistically significant genes at the same time with FDR correction. Fold change (FC) >2 and P-value <0.05 were set as the cut-off criteria. Probes without a corresponding gene symbol were then filtered.

### Prediction of mRNA-miRNA-lncRNA interactions

Interactions between differentially expressed miRNAs and differentially expressed mRNAs were predicted using miRWalk 3.0 (http://mirwalk.umm.uni-heidelberg.de/), which integrated the prediction results of both TargetScan [22] and miRDB [23], and a score ≥0.95 was considered as the cutoff criterion for the prediction analysis in miRWalk. Only the target mRNAs included in all of these databases were selected for the further analysis. The interaction between miRNA and lncRNA was predicted by using DIANA-LncBase v2.0 [24] and the score ≥0.4 was considered as cutoff criterion for the prediction analysis in the experimental module of LncBase. After the predicted targets were intersected with DEGs, miRNAs, lncRNAs and mRNAs were selected for further analysis. Cytoscape software (version 3.40) was used to visualize the regulatory network.

### Gene function analysis

The DAVID database (http://david.abcc.ncifcrf.gov/) was used to conduct gene ontology (GO) and Kyoto Encyclopedia of Genes and Genomes (KEGG) pathway analysis on significant DEGs and target genes of miRNAs with differential expression. The species was limited to Homo sapiens, and the “adjusted P-value (from the Benjamini–Hochberg method), 0.05” was considered statistically significant [25]. The GO terms included these three criteria: molecular function (MF), cellular component (CC), and biological process (BP).

### Protein-protein interaction (PPI) network construction and analysis

The protein-protein interaction network was constructed using the STRING online database. PPI pairs with a combined score ≥0.4 were used to construct the PPI network. Transcription factors were annotated using a TF checkpoint [26]. Then, the regulatory relationship between genes was visualized using Cytoscape software (version 3.4.0) and analysed through a topological property of the computing network including the degree distribution of the network using the CentiScaPe app [27]. Furthermore, the gene with a degree >5 was defined as a hub gene in the regulatory network according to a previous study [28].

### TCGA dataset analysis

TCGA is a platform for researchers to download and assess free public datasets (https://cancergenome.nih.gov/) [29]. In the present study, we verified the expression of hub genes in TCGA datasets to improve the reliability of our analysis using an online tool, UALCAN (http://ualcan.path.uab.edu/) [30]. UALCAN used TCGA level 3 RNA-seq and clinical data from 31 cancer types. As described by Li and Dewey [31], the “scaled_estimate” was multiplied by 10^6^ to obtain transcripts per million (TPM) expression value using an in-house PERL (Practical Extraction and Report Language) program. As TPM has been suggested to be more comparable across samples than fragments per kilobase of transcript per million mapped reads (FPKM) and reads per kilobase of transcript per million mapped reads (RPKM) [32], TPM was used as the measure of expression here. Fifty-two normal samples and 497 primary tumour samples of prostate adenocarcinoma (PRAD) in TCGA datasets were included in the present study. Box and whisker plots were used to show hub gene expression levels between normal samples and primary tumour samples in PRAD.

## Results

### Identification of differentially expressed mRNAs, miRNAs and lncRNAs

**Fig 1:**
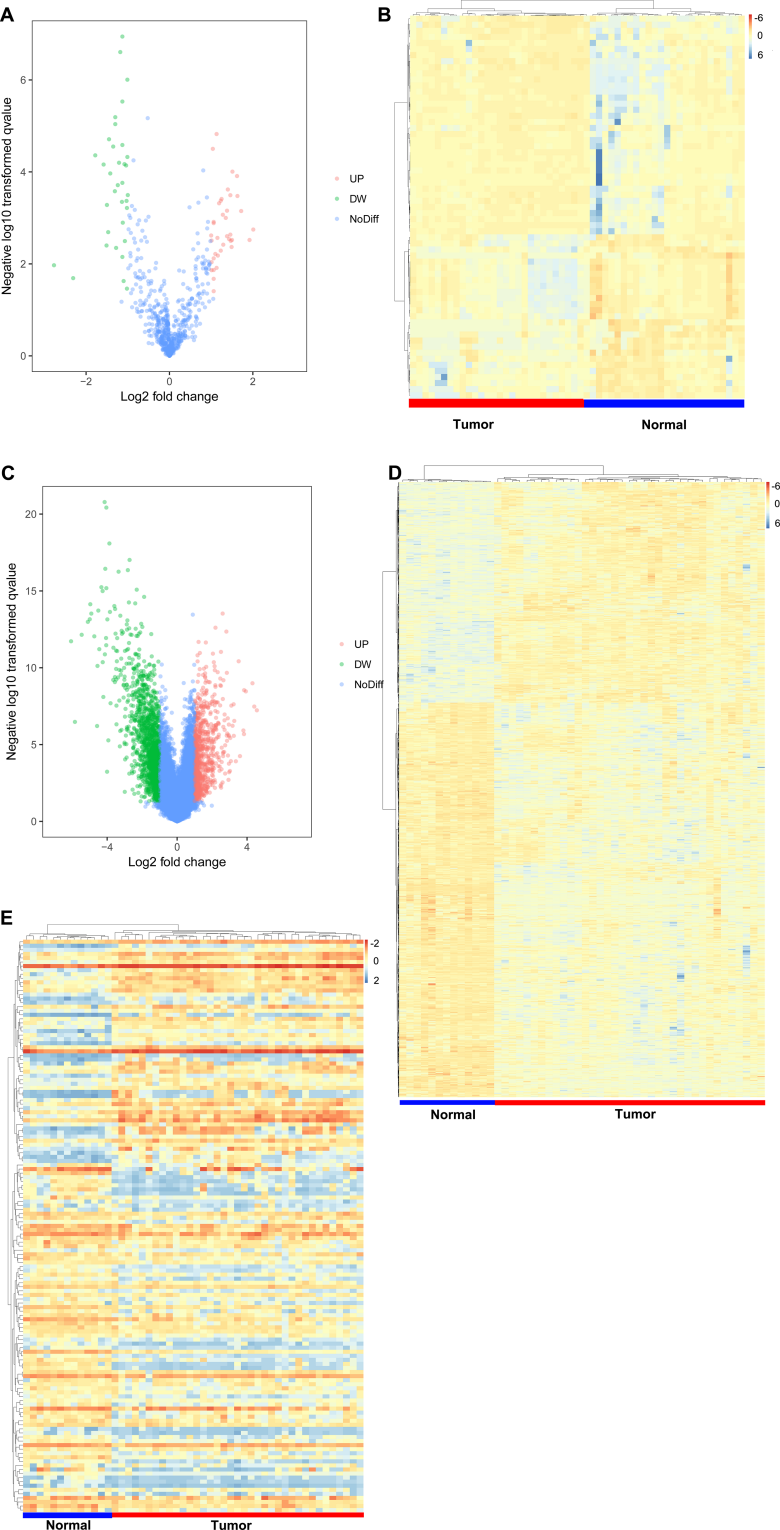
Differentially expressed genes in prostate cancer (PCa) and normal prostate tissue. (A. Volcano plot of differentially expressed miRNAs. B. Heatmap of differentially expressed miRNAs. C. Volcano plot of differentially expressed mRNAs. D. Heatmap of differentially expressed mRNAs. E. Heatmap of differentially expressed lncRNAs).

The results show that 60 miRNAs, 1578 mRNAs and 128 lncRNAs were differentially expressed in prostate cancer (S3, S4 and S5 Tables). Analysis of the GSE64318 dataset led to the identification of 60 miRNAs in PCa tumour tissues compared with normal prostate tissues, including 35 up-regulated miRNAs and 25 down-regulated miRNAs. Analysis of the GSE46602 dataset led to the identification of 1578 mRNAs, including 583 up-regulated and 995 down-regulated mRNAs, and 128 lncRNAs, including 49 up-regulated and 79 down-regulated lncRNAs. We selected all significantly up-regulated and down-regulated mRNAs, miRNAs and lncRNAs to plot their expression on heat-maps and volcano plots (Fig 1). The top 10 significantly up-regulated and down-regulated miRNAs, mRNAs and lncRNAs are shown in Tables 1, 2 and 3, respectively.

**Table 1:** Top 10 significantly up-regulated and down-regulated miRNAs in the GSE64318 dataset.

miRNA_ID	Down/Up	logFC	adj.P.Val	P.Value
hsa-miR-671-5p	Down	-1.58	0.003129	6.91E-05
hsa-miR-923	Down	-1.5	0.01131	5.23E-04
hsa-miR-483-5p	Down	-1.47	0.027441	2.04E-03
hsa-miR-92b	Down	-1.42	0.004019	1.08E-04
hsa-miR-150	Down	-1.35	0.002095	2.81E-05
hsa-miR-498	Down	-1.31	0.007999	2.63E-04
hsa-miR-933	Down	-1.3	0.000927	6.46E-06
hsa-miR-765	Down	-1.3	0.001072	9.14E-06
hsa-miR-563	Down	-1.24	0.006355	1.94E-04
hsa-miR-665	Down	-1.21	0.003129	6.32E-05
hsa-miR-221	Up	1.46	0.009099	3.21E-04
hsa-miR-454	Up	1.46	0.029644	2.31E-03
hsa-miR-26b	Up	1.47	0.034161	3.16E-03
hsa-let-7a	Up	1.5	0.034161	3.02E-03
hsa-miR-203	Up	1.51	0.003856	9.86E-05
hsa-miR-148a	Up	1.62	0.004387	1.23E-04
hsa-miR-183	Up	1.63	0.009145	3.34E-04
hsa-miR-200a	Up	1.72	0.01323	7.09E-04
hsa-miR-1	Up	1.92	0.034161	3.02E-03
hsa-let-7f	Up	2.01	0.024917	1.79E-03

**Table 2:** Top 10 significantly up-regulated and down-regulated mRNAs in the GSE46602 dataset.

Gene Symbol	Down/Up	logFC	adj.P.Val	P.Value
CD177	Down	-6.06431	2.03E-09	1.89E-12
NEFH	Down	-5.84333	3.13E-05	3.34E-07
SLC14A1	Down	-5.45679	8.83E-10	7.15E-13
KRT5	Down	-5.10424	2.09E-10	1.03E-13
KRT15	Down	-4.99578	1.56E-10	7.00E-14
TRIM29	Down	-4.96486	2.68E-11	7.36E-15
TP63	Down	-4.90695	7.76E-11	2.98E-14
JUP	Down	-4.73608	1.03E-09	9.03E-13
NEFH	Down	-4.57684	5.07E-05	6.18E-07
DST	Down	-4.55381	4.33E-08	7.60E-11
CXCL14	Up	3.356898	2.96E-03	1.22E-04
SIM2	Up	3.518509	2.60E-08	3.80E-11
F5	Up	3.592734	1.63E-03	5.63E-05
LUZP2	Up	3.789194	8.41E-05	1.21E-06
PCA3	Up	3.822882	1.28E-04	2.08E-06
AMACR	Up	3.830373	7.49E-07	2.88E-09
GLYATL1	Up	3.928912	8.57E-07	3.45E-09
AMACR	Up	4.307398	3.25E-07	1.02E-09
DLX1	Up	4.410439	5.09E-06	3.35E-08
PCA3	Up	4.558841	7.89E-06	5.87E-08

**Table 3:** Top 10 significantly up-regulated and down-regulated lncRNAs in the GSE46602 dataset.

Gene Symbol	Location	adj.P.Val	P.Value	logFC	Down/Up
LINC00844	chr10:58999518–59001617	5.83E-09	6.18E-12	-4.45143	Down
MIR205HG	chr1:209428820–209432552	4.37E-13	5.59E-17	-3.34821	Down
RP11-44F21.5	chr4:75081702–75084717	2.14E-07	6.06E-10	-3.01543	Down
RP11-680G24.5	chr16:15018106–15020488	1.49E-05	1.28E-07	-2.57293	Down
RP11-203B7.2	chr4:146052604–146056762	2.96E-07	9.10E-10	-2.25844	Down
MAGI2-AS3	chr7:79452957–79471208	1.29E-07	3.08E-10	-1.94617	Down
LSAMP-AS1	chr3:116360024–116370085	4.05E-06	2.48E-08	-1.94409	Down
LINC00937	chr12:8356963–8390752	1.63E-04	2.83E-06	-1.93046	Down
LINC00883	chr3:107240692–107326964	3.22E-04	6.79E-06	-1.86947	Down
PGM5-AS1	chr9:68355164–68357866	4.00E-02	4.20E-03	-1.86179	Down
RP5-1092A3.4	chr1:28239509–28241453	1.16E-01	2.21E-02	1.132616	Up
LINC01023	chr5:108727825–108728261	9.06E-04	2.61E-05	1.13526	Up
LINC01128	chr1:827591–859446	4.49E-02	5.00E-03	1.199525	Up
BAIAP2-AS1	chr17:81029133–81034719	1.48E-03	4.96E-05	1.201015	Up
RP11-48B3.4	chr8:80541300–80543104	1.15E-02	7.49E-04	1.284279	Up
RP11-339B21.15	chr9:128305161–128305515	6.32E-05	8.40E-07	1.310892	Up
GABPB1-AS1	chr15:50354174–50358312	8.11E-04	2.26E-05	1.367447	Up
RP11-391M1.4	chr3:40466466–40468587	7.89E-06	5.87E-08	1.489649	Up
RP11-295G20.2	chr1:231522517–231528556	7.32E-04	1.96E-05	1.673082	Up
LINC00992	chr5:117415512–117579744	1.90E-04	3.44E-06	2.288701	Up

### mRNA-miRNA-lncRNA network analysis

**Fig 2:**
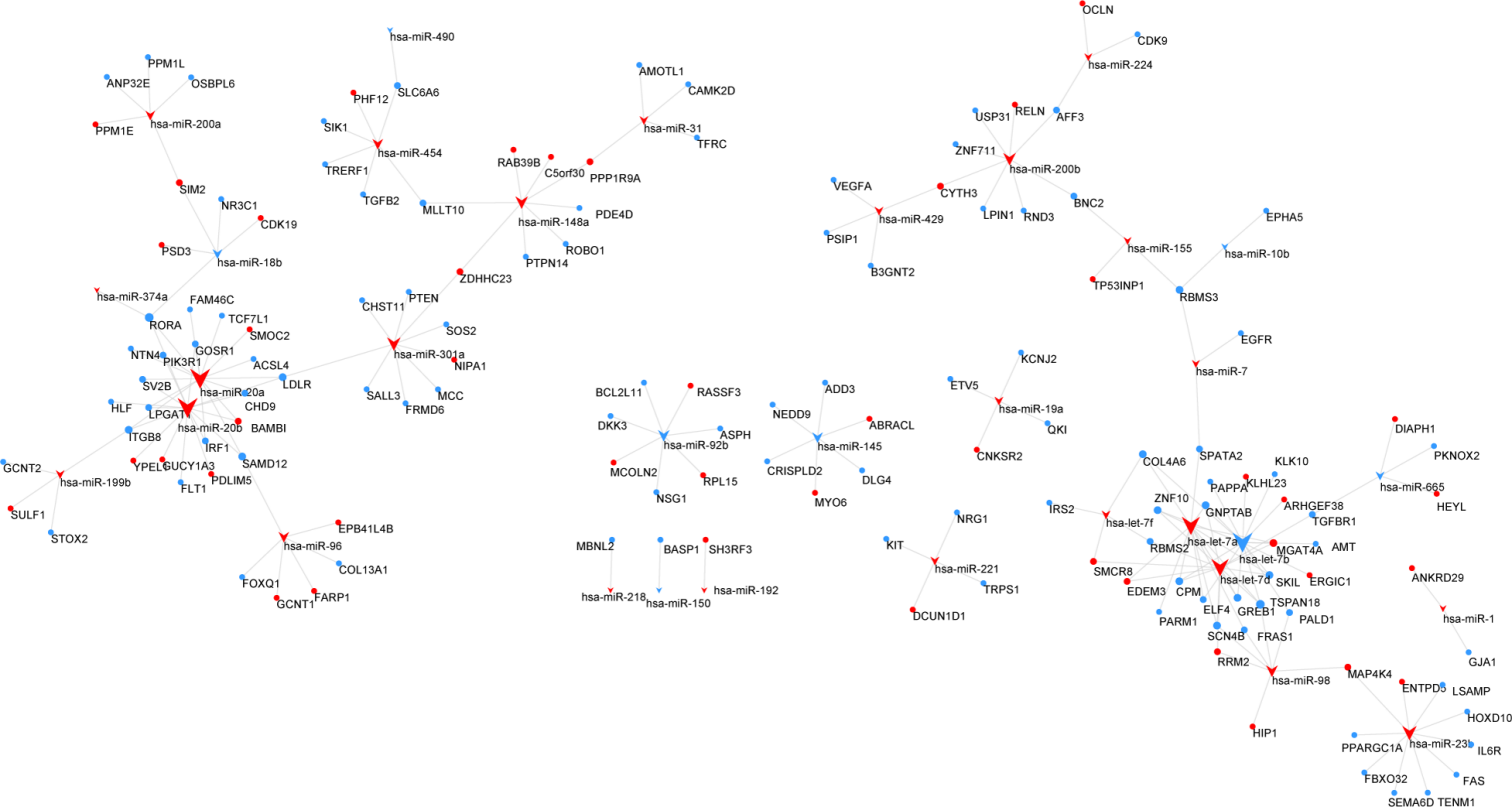
miRNA–mRNA regulatory network. Every node represents one gene, and each edge represents the interaction between genes. miRNAs are indicated with a V-shape, and mRNAs are indicated with circles. The colour red represents high expression, and blue represents low expression. There were 33 miRNAs, 143 mRNAs and 179 edges in the network.

**Fig 3:**
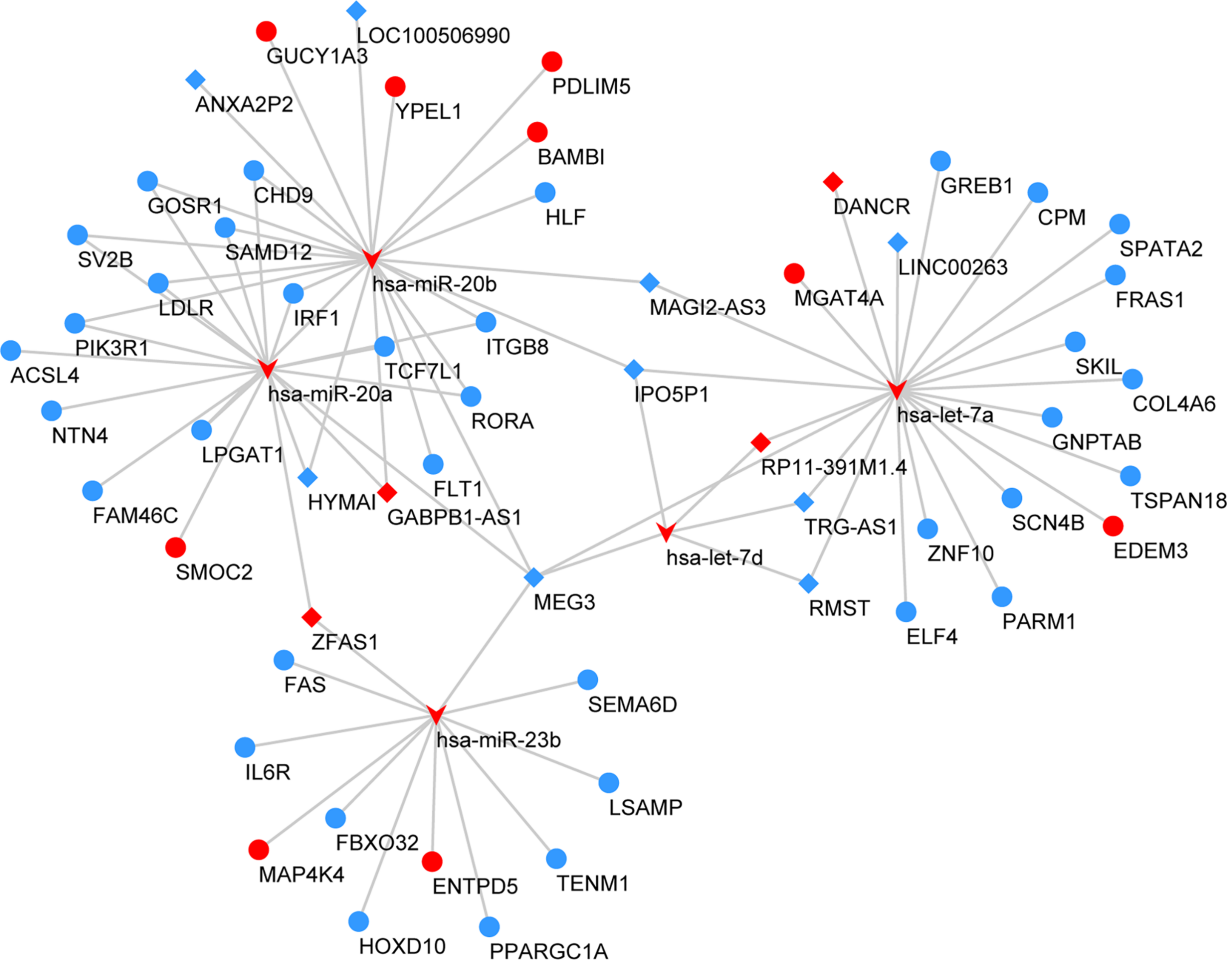
mRNA-miRNA-lncRNA regulatory network. Every node represents one gene, and each edge represents the interaction between genes. mRNAs, miRNAs and lncRNAs are indicated with circles, V-shapes and diamond shapes, respectively. The colour red represents high expression, and blue represents low expression. There were 13 lncRNAs, 5 miRNAs, 45 mRNAs and 81 edges in the network.

The miRNA–mRNA regulatory network was constructed. Interaction analysis shows that 33 differentially expressed miRNAs targeted 143 mRNAs, up-regulating or down-regulating them (Fig 2). In detail, 25 over-expressed miRNAs down-regulate 104 mRNAs, while 8 under-expressed miRNAs up-regulate 15 mRNAs. Subsequently, we reconstructed the lncRNA–miRNA–mRNA network after selecting the pre-treated data with P-values <0.05 and fold change >2 (Fig 3). In the network, there are 5 miRNA nodes, 13 lncRNA nodes, and 45 mRNA nodes. In detail, 9 under-expressed lncRNAs up-regulate 19 miRNAs, while 5 over-expressed miRNAs down-regulate 44 mRNAs.

### Function enrichment analysis of DEGs

**Fig 4:**
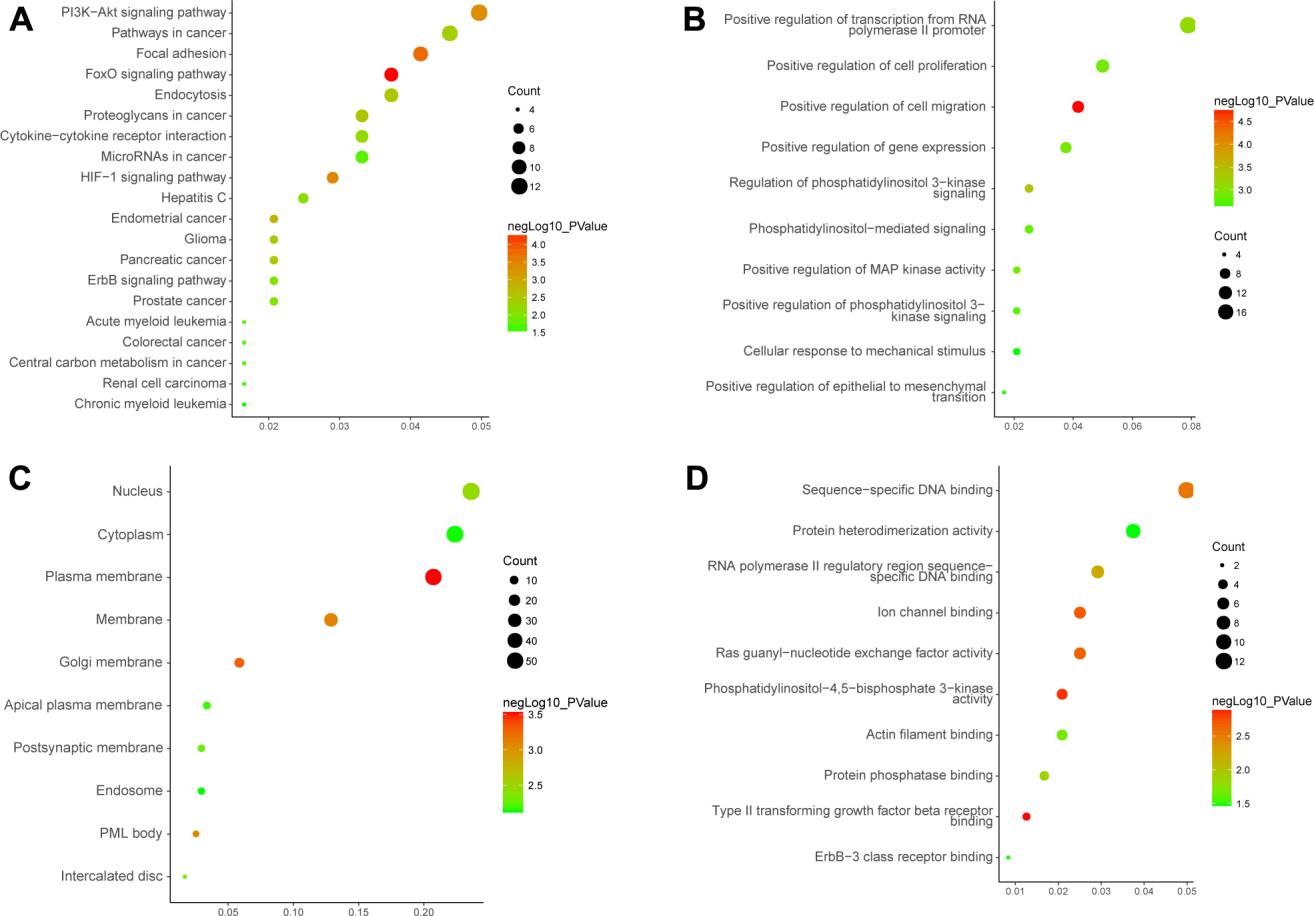
Gene ontology (GO) and Kyoto Encyclopedia of Genes and Genome (KEGG) enrichment analysis. (A. The top 20 most significant KEGG pathway terms. B. The top 10 most significant changes in the GO biological process. C. The top 10 most significant changes in the GO cellular component. D. The top 10 most significant changes in the GO molecular function.).

GO enrichment analyses for DEGs based on the mRNA-miRNA-lncRNA network were performed. The top 10 most significant GO terms of each group were shown. On the MF level, the DEGs were mainly enriched in sequence-specific DNA binding. On the CC level, the DEGs were mainly enriched in the nucleus, cytoplasm and plasma membrane. On the BP level, the DEGs were mainly enriched in positive regulation of transcription from the RNA polymerase II promoter, cell proliferation and cell migration. The top 20 most significant KEGG pathway terms are shown. The genes were mainly enriched in the PI3K-Akt signalling pathway, pathways in cancer and the focal adhesion signalling pathway (Fig 4, S1 File).

### Protein-protein interaction network (PPI) analysis

**Fig 5:**
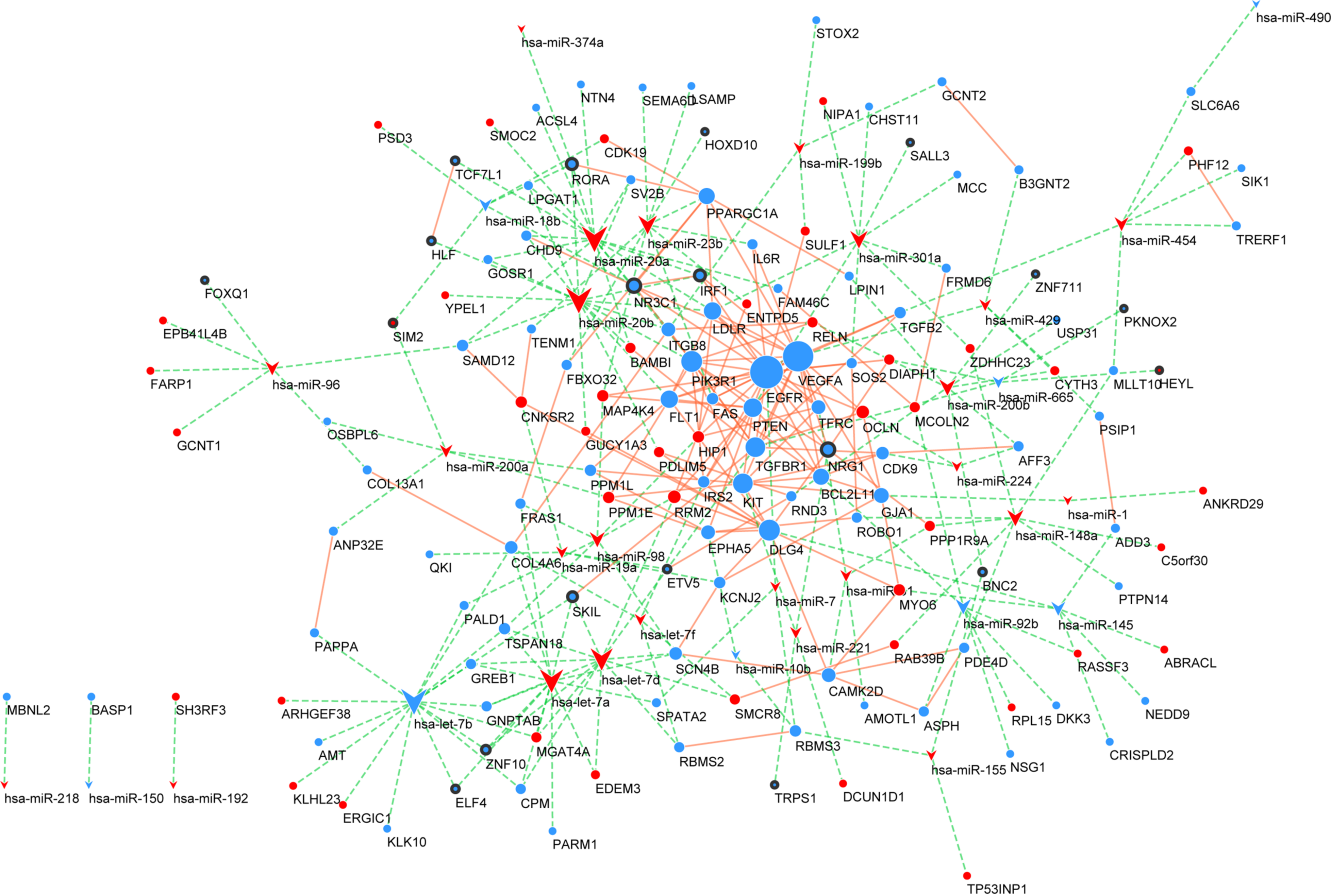
Protein-protein interaction (PPI) network. mRNAs and miRNAs are indicated with circles sand arrows, respectively. TFs are indicated with black-traced circles. The green dotted line indicates the interaction of the miRNAs-mRNAs. The orange line indicates the relationship of the interaction of the proteins. The colour red represents high expression, and green represents low expression.

Using the STRING online database and Cytoscape software, a total of 128 of the 1578 DEGs were mapped into the PPI network complex. In this network, with a degree >5, 32 nodes were chosen as hub nodes, including 2 TFs, 14 miRNAs, and 16 mRNAs; the results are presented in Table 4, Fig 5 and S2 File. Moreover, we found that 6 mRNAs (i.e., EGFR, VEGFA, PIK3R1, DLG4, TGFBR1 and KIT) exhibited higher degrees (>10) and miRNA-mRNA pairs. These findings suggest that these nodes may play important roles in the development of PCa.

**Table 4:** Topology parameters of hub genes (degree >5) in the miRNA-mRNA-TF regulatory network.

Gene	Closeness	Betweenness	Degree
EGFR	0.00212766	4046.701286	22
VEGFA	0.002159827	4331.82023	20
hsa-miR-20b	0.001855288	2752.2272	16
hsa-miR-20a	0.001821494	2973.867274	16
hsa-let-7b	0.001481481	2436.558748	16
hsa-let-7a	0.001582278	1383.644882	14
hsa-let-7d	0.00174216	2553.049636	13
PIK3R1	0.002057613	2045.442867	12
DLG4	0.001869159	3167.065846	12
TGFBR1	0.001976285	3691.770395	11
KIT	0.001904762	1517.550434	11
hsa-miR-23b	0.001587302	1375.820161	10
PTEN	0.001923077	1345.754047	10
hsa-miR-301a	0.001610306	1995.023424	9
LDLR	0.001960784	1888.001784	9
FLT1	0.001930502	1179.255452	9
hsa-miR-200b	0.001445087	1353.57487	8
hsa-miR-148a	0.00140647	1235.527073	8
PPARGC1A	0.001801802	1571.190602	8
BCL2L11	0.001834862	1606.829419	8
NR3C1*	0.00177305	733.3472529	7
NRG1*	0.001872659	1359.250724	7
hsa-miR-98	0.001618123	802.5761652	7
hsa-miR-92b	0.001506024	1655.33632	7
GJA1	0.001745201	1149.048086	7
hsa-miR-96	0.001335113	1416.966025	6
hsa-miR-454	0.001420455	1854.243032	6
hsa-miR-145	0.001461988	1257.608267	6
TFRC	0.001821494	445.8511305	6
ITGB8	0.001808318	2026.300029	6
EPHA5	0.001689189	845.4402231	6
CAMK2D	0.001620746	1045.62274	6

* transcription factors (TFs).

### TCGA dataset analysis

**Fig 6:**
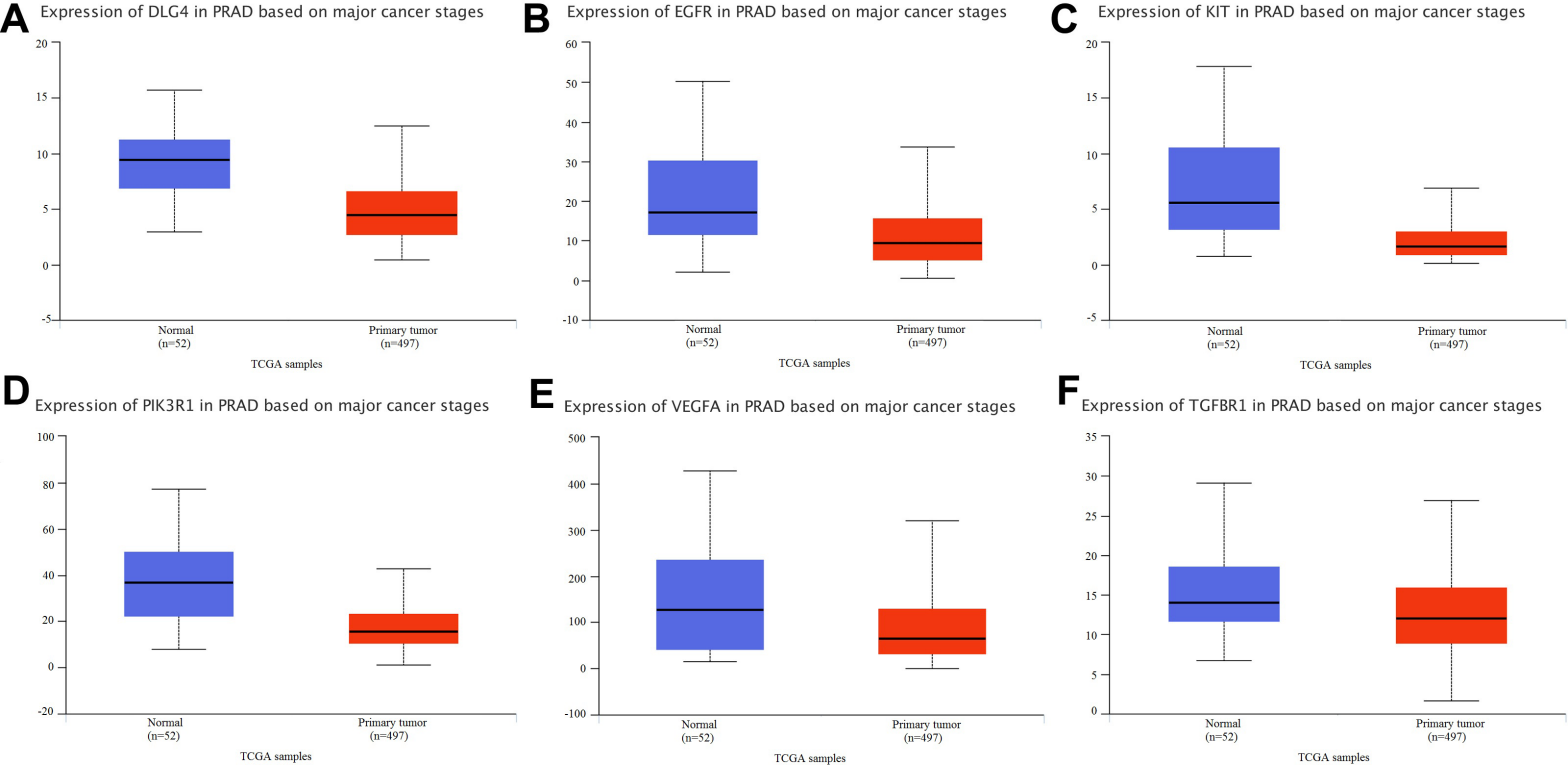
TCGA dataset analysis. (A. DLG4 expression in PCa. B. EGFR expression in PCa. C. KIT expression in PCa. D. PIK3R1 expression in PCa. E. VEGFA expression in PCa. F. TGFBR1 expression in PCa).

TCGA dataset analyses were performed to demonstrate that aberrant expression of the hub genes, including EGFR, VEGFA, PIK3R1, DLG4, TGFBR1 and KIT, were significantly different between PCa and normal prostate tissues (Fig 6).

## Discussion

Prostate cancer (PCa) has become a public health issue of great concern worldwide [33]. However, the molecular mechanisms involved in PCa progression are still unclear. Therefore, it is crucial to study the mechanism and identify molecular targets for diagnosis and treatment. A number of studies have found that the development of PCa is related to a variety of signalling pathways, and mRNAs, miRNAs, lncRNAs and TFs play important regulatory roles. In this study, we performed a comprehensive bioinformatics analysis and retrieved mRNAs, miRNAs, lncRNAs and TFs in the interaction network, revealing key genes associated with prostate cancer.

miRNA expression in tumour tissues often differs from that of normal tissue, and this differential expression affects the occurrence, development and prognosis of tumours [34,35]. Studies have confirmed that miRNA expression profiles can be used as biomarkers for the early detection, classification and prognosis of tumours [36,37]. lncRNAs act as miRNA sponges and can regulate miRNA abundance and compete with mRNA for miRNA binding [38]. By constructing an mRNA-miRNA-lncRNA network, we found that the aberrant expression of lncRNAs led to abnormal expression of 5 miRNAs (i.e., has-miR-20a, has-miR-20b, has-miR-23b, has-let-7a and has-let-7d) in PCa and thus regulated the expression of target mRNAs. A previous study demonstrated that 5 miRNAs regulate the development of prostate cancer by targeting specific mRNAs and play an important role in the regulation of prostate cancer [39–41]. The involvement of key lncRNAs including HYMAI, MEG3, IPO5P1, MAG12-AS3, RMST and TRG-AS1 are important. Among them, MEG3 is an important tumour suppressor gene that inhibits cell proliferation and induces apoptosis in PCa [42]. The discovery of these lncRNAs suggests potential diagnostic and therapeutic targets for PCa.

Next, we conducted a functional enrichment analysis of the DEGs based on the mRNA-miRNA-lncRNA network. We found that the DEGs were mainly enriched in the nucleus and cytoplasm, were involved in transcriptional regulation, were related to sequence-specific DNA binding and participated in regulation of the PI3K-Akt signalling pathway, which is related to cancer, and the focal adhesion signalling pathway. To further analyse the key genes related to PCa, we constructed a PPI network. More significantly, we found that the transcription factor NR3C1 was a hub gene that participates in regulation of the expression of multiple miRNAs (has-miR-20a, has-miR-20b, has-miR-23b). Puhr M et al. assessed NR3C1 expression and the functional significance in tissues from PCa and found that it is a key factor for the development of PCa [43]. Moreover, we found that its regulatory function is similar to MEG3 and its down-regulation leads to increased expression of miRNAs (has-miR-20a, has-miR-20b, and has-miR-23b). Based on these results, we speculate that some regulatory relationship may exist between NR3C1 and MEG3.

Finally, through PPI and TCGA dataset analyses, we found that 6 mRNAs (i.e., EGFR, VEGFA, PIK3R1, DLG4, TGFBR1 and KIT) had higher degrees and miRNA-mRNA pairs. This expression was lower and significantly different between PCa and normal prostate tissues. EGFR belongs to a family of cell membrane receptor tyrosine kinases and is a key factor in tumour cell growth and invasion [44,45]. Previous studies have demonstrated that abnormal expression of EGFR and its downstream signalling contributes to disease progression in PCa [46]. EGFR downregulation is associated with enhanced signalling [47], which can lead to the development of cancer [48]. VEGFA is a mitogen with high endothelial cell specificity that plays a major regulatory role in the development of PCa [49]. A previous study found that PIK3R1, TGFBR1 and KIT might have clinical utility in distinguishing PCa [50–52]. EGFR can regulate VEGF expression by influencing MAPK and PI3K signalling pathways[53]. TGFBR1, DLG4 and KIT are also related to the regulation of MAPK and PI3K signalling pathways[54]. Our results further confirmed the potential biological relevance of 6 mRNAs in PCa. We also found that 6 mRNAs (EGFR, VEGFA, PIK3R1, DLG4, TGFBR1 and KIT) were associated with abnormal expression of 5 miRNAs (has-miR-20a, has-miR-20b, has-miR-23b, has-let-7a and has-let-7d) in PCa. In conclusion, the hub genes that we identified may play crucial roles in PCa.

## Conclusion

We constructed and analysed mRNAs, miRNAs, lncRNAs, and TF interaction networks to reveal the key genes associated with prostate cancer. We found that 5 miRNAs (has-miR-20a, has-miR-20b, has-miR-23b, has-let-7a and has-let-7d), 6 lncRNAs (HYMAI, MEG3, IPO5P1, MAG12-AS3, RMST and TRG-AS1), 6 mRNAs (EGFR, VEGFA, PIK3R1, DLG4, TGFBR1 and KIT) and 2 TFs (NR3C1, NRG1) play important regulatory roles in the interaction network. The expression levels of EGFR, VEGFA, PIK3R1, DLG4, TGFBR1 and KIT were significantly different between PCa and normal tissues. Further research is needed to specify the molecular mechanism of these hub genes in PCa.

## Supporting information

S1 Table Clinical and histopathological variables of the study cohort in GSE64318. (DOCX)

S2 Table Clinical and histopathological variables of the study cohort in GSE46602. (DOCX)

S3 Table Differentially expressed miRNAs in the GSE64318 dataset. (DOC)

S4 Table Differentially expressed mRNAs in the GSE46602 dataset. (DOC)

S5 Table Differentially expressed lncRNAs in the GSE46602 dataset. (DOC)

S1 File GO and KEGG analysis dataset. (XLSX)

S2 File PPI analysis dataset. (XLSX)

## References

[1] Ferlay J, Shin HR, Bray F, Forman D, Mathers C, Parkin DM.Estimates of worldwide burden of cancer in 2008: GLOBOCAN 2008.Int J Cancer. 2010;127(12):2893–2917. 10.1002/ijc.25516 21351269

[2] Siegel RL, Miller KD, Jemal A. Cancer Statistics, 2017. CA Cancer J Clin. 2017; 67(1):7–30. 10.3322/caac.21387 28055103

[3] Larson SR, Zhang X, Dumpit R, Coleman I, Lakely B, Roudier M, et al Characterization of osteoblastic and osteolytic proteins in prostate cancer bone metastases. Prostate. 2013; 73(9): 932–940. 10.1002/pros.22639 23334979 PMC4214278

[4] Rajpar S, Fizazi K. Bone targeted therapies in metastatic castration-resistant prostate cancer. Cancer J. 2013;19(1):66–70. 10.1097/PPO.0b013e31827f123e 23337759

[5] Schaefer A, Jung M, Mollenkopf HJ, Wagner I, Stephan C, Jentzmik F, et al Diagnostic and prognostic implications of microRNA profiling in prostate carcinoma.Int J Cancer. 2010;126(5):1166–1176. 10.1002/ijc.24827 19676045

[6] Nguyen HC, Xie W, Yang M, Hsieh CL, Drouin S, Lee GS, et al Expression differences of circulating microRNAs in metastatic castration resistant prostate cancer and low-risk, localized prostate cancer. Prostate. 2013;73(4):346–354. 10.1002/pros.22572 22887127 PMC3980954

[7] Huang X, Yuan T, Liang M, Du M, Xia S, Dittmar R, et al Exosomal miR-1290 and miR-375 as prognostic markers in castration-resistant prostate cancer.Eur Urol. 2015;67(1):33–41. 10.1016/j.eururo.2014.07.035 25129854 PMC4252606

[8] Guttman M, Rinn JL. Modular regulatory principles of large non-coding RNAs. Nature. 2012;482(7385):339–346. 10.1038/nature10887 22337053 PMC4197003

[9] Chen L, Yao H, Wang K, Liu X. Long Non-Coding RNA MALAT1 Regulates ZEB1 Expression by Sponging miR-143-3p and Promotes Hepatocellular Carcinoma Progression. J Cell Biochem. 2017;118(12):4836–4843. 10.1002/jcb.26158 28543721

[10] Chang YT, Lin TP, Tang JT, Campbell M, Luo YL, Lu SY, et al HOTAIR is a REST-regulated lncRNA that promotes neuroendocrine differentiation in castration resistant prostate cancer. Cancer Lett. 2018; pii: S0304-3835 (18)30435-X. 10.1016/j.canlet.2018.06.029 29944905

[11] Tian C, Deng Y, Jin Y, Shi S, Bi H.Long non-coding RNA RNCR3 promotes prostate cancer progression through targeting miR-185-5p.Am J Transl Res. 2018;10(5):1562–1570 29887969 PMC5992541

[12] Li W, Dou Z, We S, Zhu Z, Pan D, Jia Z, et al Long noncoding RNA BDNF-AS is associated with clinical outcomes and has functional role in human prostate cancer. Biomed Pharmacother.2018;102:1105–1110. 10.1016/j.biopha.2018.03.118 29710528

[13] Afshar AS, Xu J, Goutsias J. Integrative Identification of Deregulated MiRNA/TF-Mediated Gene Regulatory Loops and Networks in Prostate Cancer. PLoS One. 2014; 9(6): e100806 10.1371/journal.pone.0100806 24968068 PMC4072696

[14] Xu H, He JH, Xiao ZD, Zhang QQ, Chen YQ, Zhou H, et al Liver-enriched transcription factors regulate microRNA-122 that targets CUTL1 during liver development. Hepatology. 2010;52(4):1431–1442. 10.1002/hep.23818 20842632

[15] Sunkel B, Wu D, Chen Z, Wang CM, Liu X, Ye Z, et al Integrative analysis identifies targetable CREB1/FoxA1 transcriptional co-regulation as a predictor of prostate cancer recurrence.Nucleic Acids Res. 2016;44(9):4105–4122. 10.1093/nar/gkv1528 26743006 PMC4872073

[16] Vogelstein B, Papadopoulos N, Velculescu VE, Zhou S, Diaz LA Jr, Kinzler KW. Cancer genome landscapes. Science. 2013; 339(6127): 1546–1558. 10.1126/science.1235122 23539594 PMC3749880

[17] Guo Y, Bao Y, Ma M, Yang W. Identification of Key Candidate Genes and Pathways in Colorectal Cancer by Integrated Bioinformatical Analysis. Int J Mol Sci. 2017;18(4). pii: E722. 10.3390/ijms18040722 28350360 PMC5412308

[18] Wang BD, Ceniccola K, Yang Q, Andrawis R, Patel V, Ji Y, et al Identification and Functional Validation of Reciprocal microRNA-mRNA Pairings in African American Prostate Cancer Disparities.Clin Cancer Res. 2015;21(21):4970–4984. 10.1158/1078-0432.CCR-14-1566 26089375 PMC4631799

[19] Mortensen MM, Høyer S, Lynnerup AS, Ørntoft TF, Sørensen KD, Borre M2,et al Expression profiling of prostate cancer tissue delineates genes associated with recurrence after prostatectomy.Sci Rep. 2015;5:16018 10.1038/srep16018 26522007 PMC4629186

[20] Edgar R, Domrachev M, Lash AE. Gene Expression Omnibus: NCBI gene expression and hybridization array data repository. Nucleic Acids Res. 2002; 30(1): 207–210. 11752295 10.1093/nar/30.1.207 PMC99122

[21] Diboun I, Wernisch L, Orengo CA, Koltzenburg M. Microarray analysis after RNA amplification can detect pronounced differences in gene expression using limma. BMC Genomics. 2006;7:252 10.1186/1471-2164-7-252 17029630 PMC1618401

[22] Lewis BP, Burge CB, Bartel DP. Conserved seed pairing, often flanked by adenosines, indicates that thousands of human genes are microRNA targets. Cell.2005; 120 (1): 15–20. 10.1016/j.cell.2004.12.035 477 15652477

[23] Wong N, Wang X.miRDB: an online resource for microRNA target prediction and functional annotations.Nucleic Acids Res. 2015;43(Database issue):D146–152. 10.1093/nar/gku1104 25378301 PMC4383922

[24] Paraskevopoulou MD, Vlachos IS, Karagkouni D, Georgakilas G, Kanellos I, Vergoulis T, et al DIANA-LncBase v2: indexing microRNA targets on non-coding transcripts.Nucleic Acids Res. 2016;44(D1):D231–238. 10.1093/nar/gkv1270 26612864 PMC4702897

[25] Ashburner M, Ball CA, Blake JA, Botstein D, Butler H, Cherry JM, et al Gene ontology: tool for the unification of biology. The Gene Ontology Consortium. Nat Genet. 2000;25(1): 25–29. 10.1038/75556 10802651 PMC3037419

[26] Chawla K, Tripathi S, Thommesen L, Lægreid A, Kuiper M.TFcheckpoint: a curated compendium of specific DNA-binding RNA polymerase II transcription factors.Bioinformatics. 2013;29(19):2519–2520. 10.1093/bioinformatics/btt432 23933972

[27] Scardoni G, Petterlini M, Laudanna C.Analyzing biological network parameters with CentiScaPe. Bioinformatics.2009;25(21):2857–2859. 10.1093/bioinformatics/btp517 19729372 PMC2781755

[28] Han JD, Bertin N, Hao T, Goldberg DS, Berriz GF, Zhang LV, et al Evidence for dynamically organized modularity in the yeast protein-protein interaction network. Nature. 2004;430(6995):88–93. 10.1038/nature02555 15190252

[29] Tomczak K, Czerwinska P, Wiznerowicz M. The Cancer Genome Atlas (TCGA): an immeasurable source of knowledge. Contemp Oncol (Pozn). 2015;19(1A): A68–77. 10.5114/wo.2014.47136 25691825 PMC4322527

[30] Chandrashekar DS, Bashel B, Balasubramanya SAH, Creighton CJ, Ponce-Rodriguez I, Chakravarthi BVSK, et al UALCAN: A Portal for Facilitating Tumor Subgroup Gene Expression and Survival Analyses.Neoplasia. 2017;19(8):649–658. 10.1016/j.neo.2017.05.002 28732212 PMC5516091

[31] Li B, Dewey CN.RSEM: accurate transcript quantification from RNA-Seq data with or without a reference genome.BMC Bioinformatics. 2011;12:323 10.1186/1471-2105-12-323 21816040 PMC3163565

[32] Li B, Ruotti V, Stewart RM, Thomson JA, Dewey CN.RNA-Seq gene expression estimation with read mapping uncertainty.Bioinformatics. 2010;26(4):493–500. 10.1093/bioinformatics/btp692 20022975 PMC2820677

[33] Mano R, Eastham J, Yossepowitch O. The very-high-risk prostate cancer: a contemporary update. Prostate Cancer Prostatic Dis. 2016; 19(4): 340–348. 10.1038/pcan.2016.40 27618950 PMC5559730

[34] Song C, Chen H, Wang T, Zhang W, Ru G, Lang J. Expression profile analysis of micro RNAs in prostate cancer by next-generation sequencing. Prostate. 2015; 75(5): 500–516. 10.1002/pros.22936 25597612

[35] Avril S. [micro RNA expression in breast development and breast cancer]. Pathologe. 2013;34 Suppl 2: 195–200. 10.1007/s00292-013-1878-7 24196612

[36] Ferracin M, Veronese A, Negrini M. Micromarkers: miRNAs in cancer diagnosis and prognosis. Expert Rev Mol Diagn. 2010;10(3):297–308. 10.1586/erm.10.11 20370587

[37] Søkilde R, Vincent M, Møller AK, Hansen A, Høiby PE, Blondal T, et al Efficient identification of miRNAs for classification of tumor origin. J Mol Diagn.2014;16(1):106–115. 10.1016/j.jmoldx.2013.10.001 24211363

[38] Wang JB, Liu FH, Chen JH, Ge HT, Mu LY, Bao HB, et al Identifying survival-associated modules fromthe dysregulated triplet network in glioblastoma multiforme. J Cancer Res Clin Oncol. 2017;143(4):661–671. 10.1007/s00432-016-2332-z 28168356 PMC11819112

[39] Liu DF, Wu JT, Wang JM, Liu QZ, Gao ZL, Liu YX. MicroRNA expression profile analysis reveals diagnostic biomarker for human prostate cancer. Asian Pac J Cancer Prev. 2012;13(7):3313–3317. 22994753 10.7314/apjcp.2012.13.7.3313

[40] Cai S, Chen R, Li X, Cai Y, Ye Z, Li S, et al Downregulation of microRNA-23a suppresses prostate cancer metastasis by targeting the PAK6-LIMK1 signaling pathway. Oncotarget. 2015;6(6):3904–3917. 10.18632/oncotarget.2880 25714010 PMC4414162

[41] Wagner S, Ngezahayo A, Murua Escobar H, Nolte I. Role of miRNA let-7 and its major targets in prostate cancer. Biomed Res Int. 2014;2014:376326 10.1155/2014/376326 25276782 PMC4168040

[42] Luo G, Wang M, Wu X, Tao D, Xiao X, Wang L, et al Long Non-Coding RNA MEG3 Inhibits Cell Proliferation and Induces Apoptosis in Prostate Cancer. Cell Physiol Biochem. 2015;37(6):2209–2220. 10.1159/000438577 26610246

[43] Puhr M, Hoefer J, Eigentler A, Ploner C, Handle F, Schaefer G, et al The glucocorticoid receptor is a key player for prostate cancer cell survival and a target for improved antiandrogen therapy. Clin Cancer Res. 2018;24(4):927–938. 10.1158/1078-0432.CCR-17-0989 29158269

[44] Lim SO, Li CW, Xia W, Lee HH, Chang SS, Shen J, et al EGFR Signaling Enhances Aerobic Glycolysis in Triple-Negative Breast Cancer Cells to Promote Tumor Growth and Immune Escape. Cancer Res. 2016;76(5):1284–1296. 10.1158/0008-5472.CAN-15-2478 CAN-15-2478 26759242 PMC4775355

[45] Daizumoto K, Yoshimaru T, Matsushita Y, Fukawa T, Uehara H, Ono M, et al A DDX31/mutant-p53/EGFR axis promotes multistep progression of muscle invasive bladder cancer. Cancer Res. 2018;78(9):2233–2247. 10.1158/0008-5472.CAN-17-2528 29440146

[46] Di Lorenzo G, Tortora G, D'Armiento FP, De Rosa G, Staibano S, Autorino R, et al Expression of epidermal growth factor receptor correlates with disease relapse and progression to androgen-independence in human prostate cancer. Clin Cancer Res. 2002; 8(11): 3438–3444. 12429632

[47] Sorkin A, von Zastrow M. Endocytosis and signalling: intertwining molecular networks. Nat Rev Mol Cell Biol. 2009;10(9):609–622. 10.1038/nrm2748 19696798 PMC2895425

[48] Roepstorff K, Grøvdal L, Grandal M, Lerdrup M, van Deurs B. Endocytic downregulation of ErbB receptors: mechanisms and relevance in cancer. Histochem Cell Biol. 2008; 129(5): 563–578. 10.1007/s00418-008-0401-3 18288481 PMC2323030

[49] Lee C, Whang YM, Campbell P, Mulcrone PL, Elefteriou F, Cho SW, et al Dual targeting c-met and VEGFR2 in osteoblasts suppresses growth and osteolysis of prostate cancer bone metastasis. Cancer Lett. 2018;414:205–213. 10.1016/j.canlet.2017.11.016 29174801

[50] Hellwinkel OJ, Rogmann JP, Asong LE, Luebke AM, Eichelberg C, Ahyai S, et al A comprehensive analysis of transcript signatures of the phosphatidylinositol-3 kinase/protein kinase B signal-transduction pathway in prostate cancer. BJU Int. 2008; 101(11):1454–1460. 10.1111/j.1464-410X.2008.07540.x 18336616

[51] Wen J, Li R, Wen X, Chou G, Lu J, Wang X, et al Dysregulation of cell cycle related genes and microRNAs distinguish the low- from high-risk of prostate cancer. Diagn Pathol. 2014;9:156 10.1186/s13000-014-0156-1 25257132 PMC4215008

[52] Fonseca-Alves CE, Kobayashi PE, Palmieri C, Laufer-Amorim R. Investigation of c-KIT and Ki67 expression in normal, preneoplastic and neoplastic canine prostate. BMC Vet Res. 2017;13(1):380 10.1186/s12917-017-1304-0 29207991 PMC5718037

[53] Song ZY, Wang F, Cui SX, Qu XJ. Knockdown of CXCR4 Inhibits CXCL12-Induced Angiogenesis in HUVECs through Downregulation of the MAPK/ERK and PI3K/AKT and the Wnt/β-Catenin Pathways.Cancer Invest. 2018;36(1):10–18. 10.1080/07357907.2017.1422512 29381400

[54] Hamidi A, Song J, Thakur N1, Itoh S, Marcusson A, Bergh A, et al TGF-β promotes PI3K-AKT signaling and prostate cancer cell migration through the TRAF6-mediated ubiquitylation of p85α. Sci Signal. 2017;10(486): pii: eaal4186. 10.1126/scisignal.aal4186 28676490

