# Supplementary material for: Construction and analysis of mRNA, miRNA, lncRNA, and TF regulatory networks reveal the key genes associated with prostate cancer
Source: PLoS One. 2018 Aug 23;13(8):e0198055. doi: 10.1371/journal.pone.0198055 (PMC6107126; doi:10.1371/journal.pone.0198055)
Supplement: S1 Table — (DOCX) [file pone.0198055.s001.docx]

**Table S1. Clinical and histopathological variables of the study cohort in GSE64318**

| **Patient ID** | **Race** | **Age** | **Biopsy samples** | **Gleason Score** |
| --- | --- | --- | --- | --- |
| 4 | AA | 70 | tumor | 6 |
| 4 | AA | 70 | normal |  |
| 10 | AA | 60 | tumor | 6 |
| 10 | AA | 60 | normal |  |
| 13 | AA | 74 | tumor | 6 |
| 13 | AA | 74 | normal |  |
| 17 | AA | 67 | tumor | 6 |
| 17 | AA | 67 | normal |  |
| 19 | AA | 68 | tumor | 6 |
| 19 | AA | 68 | normal |  |
| 29 | AA | 58 | tumor | 7 |
| 29 | AA | 58 | normal |  |
| 43 | AA | 60 | tumor | 6 |
| 43 | AA | 60 | normal |  |
| 48 | AA | 52 | tumor | 7 |
| 48 | AA | 52 | normal |  |
| 49 | AA | 69 | tumor | 7 |
| 49 | AA | 69 | normal |  |
| 51 | AA | 56 | tumor | 6 |
| 51 | AA | 56 | normal |  |
| 75 | AA | 64 | tumor | 7 |
| 75 | AA | 64 | normal |  |
| 106 | AA | 58 | tumor | 7 |
| 106 | AA | 58 | normal |  |
| 115 | AA | 65 | tumor | 6 |
| 115 | AA | 65 | normal |  |
| 120 | AA | 58 | tumor | 7 |
| 120 | AA | 58 | normal |  |
| 9 | EA | 49 | tumor | 6 |
| 9 | EA | 49 | normal |  |
| 16 | EA | 62 | tumor | 6 |
| 16 | EA | 62 | normal |  |
| 18 | EA | 59 | tumor | 7 |
| 18 | EA | 59 | normal |  |
| 28 | EA | 61 | tumor | 6 |
| 28 | EA | 61 | normal |  |
| 30 | EA | 72 | tumor | 6 |
| 30 | EA | 72 | normal |  |
| 44 | EA | 60 | tumor | 6 |
| 44 | EA | 60 | normal |  |
| 57 | EA | 70 | tumor | 6 |
| 57 | EA | 70 | normal |  |
| 58 | EA | 65 | tumor | 6 |
| 58 | EA | 65 | normal |  |
| 60 | EA | 64 | tumor | 7 |
| 60 | EA | 64 | normal |  |
| 105 | EA | 68 | tumor | 8 |
| 105 | EA | 68 | normal |  |
| 121 | EA | 54 | tumor | 7 |
| 121 | EA | 54 | normal |  |
| 124 | EA | 50 | tumor | 6 |
| 124 | EA | 50 | normal |  |
| 139 | EA | 59 | tumor | 7 |
| 139 | EA | 59 | normal |  |

AA= African Americans

EA= European American
