# Supplementary material for: Construction and analysis of mRNA, miRNA, lncRNA, and TF regulatory networks reveal the key genes associated with prostate cancer
Source: PLoS One. 2018 Aug 23;13(8):e0198055. doi: 10.1371/journal.pone.0198055 (PMC6107126; doi:10.1371/journal.pone.0198055)
Supplement: S2 Table — (DOCX) [file pone.0198055.s002.docx]

**Table S2. Clinical and histopathological variables of the study cohort in GSE46602**

| Clinical Variable | Study cohort | | |
| --- | --- | --- | --- |
| Age median(range) Years | 63 (46–71) | | |
| Gleason grade | | |  |
| Low (5–6) | | 17 (47%) |  |
| Intermediate (7) | | 15 (42%) |  |
| High (8–10) | | 4 (11%) |  |
| Pathological stage | | |  |
| T2a–c | | 19 (53%) |  |
| T3a–b | | 17 (47%) |  |
| TxN+ | | 0 |  |
| Time to recurrence (range) Years | | 1.3 (0.1–6.2) |  |
| Follow up non-recurrent cases Years | | 5.5 (2.6–6.7) |  |
| Recurrence status | | |  |
| Yes | | 22 (61%) |  |
| No | | 14 (39%) |  |
| Pre-operative PSA (range) | | 16.0 (5.3–42.5) |  |
| Margin status | | |  |
| Positive | | 16 (44%) |  |
| Negative | | 20 (56%) |  |
