# Supplementary material for: Construction and analysis of mRNA, miRNA, lncRNA, and TF regulatory networks reveal the key genes associated with prostate cancer
Source: PLoS One. 2018 Aug 23;13(8):e0198055. doi: 10.1371/journal.pone.0198055 (PMC6107126; doi:10.1371/journal.pone.0198055)
Supplement: S3 Table — (DOC) [file pone.0198055.s003.doc]

**Table S3. T**he differentially expressed miRNAs in GSE64318 dataset

| ID | adj.P.Val | P.Value | t | B | logFC | miRNA_ID |
| --- | --- | --- | --- | --- | --- | --- |
| hsa-miR-671-5p | 0.003129 | 6.91E-05 | -4.32 | 1.5076 | -1.58 | hsa-miR-671 |
| hsa-miR-923 | 0.01131 | 5.23E-04 | -3.69 | -0.3743 | -1.5 | hsa-miR-923 |
| hsa-miR-483-5p | 0.027441 | 2.04E-03 | -3.24 | -1.621 | -1.47 | hsa-miR-483 |
| hsa-miR-92b | 0.004019 | 1.08E-04 | -4.19 | 1.0928 | -1.42 | hsa-miR-92b |
| hsa-miR-150 | 0.002095 | 2.81E-05 | -4.59 | 2.3499 | -1.35 | hsa-miR-150 |
| hsa-miR-498 | 0.007999 | 2.63E-04 | -3.91 | 0.2624 | -1.31 | hsa-miR-498 |
| hsa-miR-933 | 0.000927 | 6.46E-06 | -5.01 | 3.7301 | -1.3 | hsa-miR-933 |
| hsa-miR-765 | 0.001072 | 9.14E-06 | -4.91 | 3.4037 | -1.3 | hsa-miR-765 |
| hsa-miR-563 | 0.006355 | 1.94E-04 | -4.01 | 0.5474 | -1.24 | hsa-miR-563 |
| hsa-miR-665 | 0.003129 | 6.32E-05 | -4.35 | 1.5902 | -1.21 | hsa-miR-665 |
| hsa-miR-671-3p | 0.000102 | 2.48E-07 | -5.92 | 6.8069 | -1.18 | hsa-miR-671 |
| hsa-let-7f-1 | 0.01052 | 4.48E-04 | -3.74 | -0.2315 | -1.14 | hsa-let-7f-1 |
| hsa-miR-659 | 9.32E-05 | 1.14E-07 | -6.13 | 7.544 | -1.13 | hsa-miR-659 |
| hsa-miR-490-5p | 0.000605 | 2.95E-06 | -5.23 | 4.4696 | -1.13 | hsa-miR-490 |
| hsa-miR-550 | 0.002095 | 2.60E-05 | -4.61 | 2.421 | -1.13 | hsa-miR-550 |
| hsa-miR-483-3p | 0.005903 | 1.73E-04 | -4.04 | 0.6539 | -1.13 | hsa-miR-483 |
| hsa-miR-513a-5p | 0.06164 | 7.06E-03 | -2.8 | -2.7408 | -1.13 | hsa-miR-513a |
| hsa-miR-371-5p | 0.019339 | 1.27E-03 | -3.4 | -1.1918 | -1.12 | hsa-miR-371 |
| hsa-miR-513c | 0.132017 | 2.36E-02 | -2.33 | -3.8033 | -1.1 | hsa-miR-513c |
| hsa-miR-602 | 0.003129 | 6.78E-05 | -4.33 | 1.5251 | -1.08 | hsa-miR-602 |
| hsa-miR-768-5p | 0.034161 | 3.19E-03 | -3.09 | -2.0276 | -1.07 | hsa-miR-768 |
| hsa-miR-887 | 0.003129 | 7.24E-05 | -4.31 | 1.4632 | -1.04 | hsa-miR-887 |
| hsa-miR-425 | 0.010307 | 4.27E-04 | -3.76 | -0.1858 | -1.03 | hsa-miR-425 |
| hsa-let-7b | 0.012173 | 6.08E-04 | -3.65 | -0.5122 | -1.02 | hsa-let-7b |
| hsa-miR-145 | 0.000271 | 9.88E-07 | -5.53 | 5.4998 | -1.01 | hsa-miR-145 |
| hsa-miR-584 | 0.002781 | 4.74E-05 | -4.43 | 1.8589 | -1.01 | hsa-miR-584 |
| hsa-miR-767-3p | 0.009099 | 3.19E-04 | -3.85 | 0.0841 | -1 | hsa-miR-767 |
| hsa-miR-192 | 0.030485 | 2.41E-03 | 3.19 | -1.7744 | 1 | hsa-miR-192 |
| hsa-miR-374b | 0.034161 | 3.11E-03 | 3.1 | -2.0029 | 1.01 | hsa-miR-374b |
| hsa-miR-224 | 0.073062 | 9.17E-03 | 2.71 | -2.9734 | 1.01 | hsa-miR-224 |
| hsa-miR-218 | 0.090777 | 1.37E-02 | 2.55 | -3.3288 | 1.01 | hsa-miR-218 |
| hsa-miR-23b* | 0.002153 | 3.15E-05 | 4.55 | 2.2427 | 1.04 | hsa-miR-23b |
| hsa-miR-95 | 0.018957 | 1.22E-03 | 3.42 | -1.155 | 1.06 | hsa-miR-95 |
| hsa-miR-10b | 0.019339 | 1.30E-03 | 3.4 | -1.2072 | 1.06 | hsa-miR-10b |
| hsa-miR-96 | 0.063508 | 7.58E-03 | 2.78 | -2.8046 | 1.06 | hsa-miR-96 |
| hsa-miR-155 | 0.090777 | 1.36E-02 | 2.55 | -3.3217 | 1.06 | hsa-miR-155 |
| hsa-miR-30e* | 0.121718 | 2.11E-02 | 2.38 | -3.7031 | 1.06 | hsa-miR-30e |
| hsa-miR-19a | 0.192829 | 3.92E-02 | 2.11 | -4.2361 | 1.06 | hsa-miR-19a |
| hsa-miR-20b | 0.055443 | 6.08E-03 | 2.86 | -2.6073 | 1.1 | hsa-miR-20b |
| hsa-miR-29b-1* | 0.001539 | 1.50E-05 | 4.77 | 2.9384 | 1.13 | hsa-miR-29b-1 |
| hsa-miR-429 | 0.086784 | 1.22E-02 | 2.59 | -3.2288 | 1.13 | hsa-miR-429 |
| hsa-miR-199b-5p | 0.032889 | 2.72E-03 | 3.14 | -1.8842 | 1.15 | hsa-miR-199b |
| hsa-miR-199b-3p | 0.05942 | 6.59E-03 | 2.83 | -2.6791 | 1.16 | hsa-miR-199b |
| hsa-miR-18b | 0.010665 | 4.81E-04 | 3.72 | -0.2955 | 1.19 | hsa-miR-18b |
| hsa-miR-505* | 0.010307 | 4.24E-04 | 3.76 | -0.1791 | 1.23 | hsa-miR-505 |
| hsa-miR-200b | 0.048033 | 5.15E-03 | 2.92 | -2.4586 | 1.23 | hsa-miR-200b |
| hsa-miR-31* | 0.009936 | 3.87E-04 | 3.79 | -0.0958 | 1.25 | hsa-miR-31 |
| hsa-miR-20a | 0.015583 | 8.54E-04 | 3.54 | -0.8251 | 1.28 | hsa-miR-20a |
| hsa-miR-301a | 0.038702 | 3.82E-03 | 3.03 | -2.1896 | 1.29 | hsa-miR-301a |
| hsa-miR-200b* | 0.016835 | 1.00E-03 | 3.48 | -0.9743 | 1.35 | hsa-miR-200b |
| hsa-miR-374a | 0.01323 | 6.97E-04 | 3.6 | -0.6387 | 1.38 | hsa-miR-374a |
| hsa-miR-98 | 0.031037 | 2.50E-03 | 3.18 | -1.8046 | 1.38 | hsa-miR-98 |
| hsa-miR-7 | 0.007623 | 2.41E-04 | 3.94 | 0.3421 | 1.4 | hsa-miR-7 |
| hsa-let-7d | 0.033856 | 2.89E-03 | 3.12 | -1.9368 | 1.43 | hsa-let-7d |
| hsa-miR-221* | 0.009099 | 3.21E-04 | 3.85 | 0.0767 | 1.46 | hsa-miR-221 |
| hsa-miR-454 | 0.029644 | 2.31E-03 | 3.2 | -1.7349 | 1.46 | hsa-miR-454 |
| hsa-miR-26b | 0.034161 | 3.16E-03 | 3.09 | -2.0195 | 1.47 | hsa-miR-26b |
| hsa-let-7a | 0.034161 | 3.02E-03 | 3.11 | -1.9779 | 1.5 | hsa-let-7a |
| hsa-miR-203 | 0.003856 | 9.86E-05 | 4.21 | 1.1748 | 1.51 | hsa-miR-203 |
| hsa-miR-148a | 0.004387 | 1.23E-04 | 4.15 | 0.9697 | 1.62 | hsa-miR-148a |
| hsa-miR-183 | 0.009145 | 3.34E-04 | 3.84 | 0.0406 | 1.63 | hsa-miR-183 |
| hsa-miR-200a | 0.01323 | 7.09E-04 | 3.6 | -0.6539 | 1.72 | hsa-miR-200a |
| hsa-miR-1 | 0.034161 | 3.02E-03 | 3.11 | -1.9782 | 1.92 | hsa-miR-1 |
| hsa-let-7f | 0.024917 | 1.79E-03 | 3.29 | -1.5028 | 2.01 | hsa-let-7f |
