# Supplementary material for: Construction and analysis of mRNA, miRNA, lncRNA, and TF regulatory networks reveal the key genes associated with prostate cancer
Source: PLoS One. 2018 Aug 23;13(8):e0198055. doi: 10.1371/journal.pone.0198055 (PMC6107126; doi:10.1371/journal.pone.0198055)
Supplement: S4 Table — (DOC) [file pone.0198055.s004.doc]

**Table S4.** **The differentially expressed mRNAs in GSE46602 dataset**

| ID | adj.P.Val | P.Value | t | B | logFC | Gene Symbol |
| --- | --- | --- | --- | --- | --- | --- |
| 219669_at | 2.03E-09 | 1.89E-12 | -9.34251 | 18.12148 | -6.06431 | CD177 |
| 33767_at | 3.13E-05 | 3.34E-07 | -5.9022 | 6.511364 | -5.84333 | NEFH |
| 229151_at | 8.83E-10 | 7.15E-13 | -9.63073 | 19.04981 | -5.45679 | SLC14A1 |
| 201820_at | 2.09E-10 | 1.03E-13 | -10.2134 | 20.89404 | -5.10424 | KRT5 |
| 204734_at | 1.56E-10 | 7.00E-14 | -10.3317 | 21.26268 | -4.99578 | KRT15 |
| 202504_at | 2.68E-11 | 7.36E-15 | -11.0292 | 23.39803 | -4.96486 | TRIM29 |
| 211194_s_at | 7.76E-11 | 2.98E-14 | -10.594 | 22.07363 | -4.90695 | TP63 |
| 205157_s_at | 1.03E-09 | 9.03E-13 | -9.56114 | 18.82663 | -4.73608 | JUP |
| 204412_s_at | 5.07E-05 | 6.18E-07 | -5.72792 | 5.920458 | -4.57684 | NEFH |
| 204455_at | 4.33E-08 | 7.60E-11 | -8.26936 | 14.58112 | -4.55381 | DST |
| 209863_s_at | 5.79E-11 | 1.90E-14 | -10.7325 | 22.49807 | -4.50602 | TP63 |
| 230577_at | 5.83E-09 | 6.18E-12 | -8.99448 | 16.98711 | -4.45143 | LINC00844 |
| 205856_at | 3.92E-12 | 5.74E-16 | -11.8438 | 25.8045 | -4.33077 | SLC14A1 |
| 203798_s_at | 5.06E-12 | 1.02E-15 | -11.6586 | 25.26575 | -4.26065 | VSNL1 |
| 211106_at | 2.84E-08 | 4.34E-11 | -8.43038 | 15.11993 | -4.25402 | SUPT3H |
| 203434_s_at | 1.68E-06 | 7.97E-09 | -6.95309 | 10.10717 | -4.21942 | MME |
| 205765_at | 9.01E-17 | 1.65E-21 | -16.4085 | 37.56028 | -4.14649 | CYP3A5 |
| 216918_s_at | 1.06E-08 | 1.34E-11 | -8.76891 | 16.2445 | -4.13087 | DST |
| 209270_at | 3.97E-13 | 3.63E-17 | -12.7575 | 28.38963 | -4.10754 | LAMB3 |
| 244581_at | 1.29E-09 | 1.16E-12 | -9.4855 | 18.58335 | -4.06509 | ZBTB20 |
| 204971_at | 3.40E-09 | 3.42E-12 | -9.16813 | 17.5549 | -4.05405 | CSTA |
| 218963_s_at | 1.03E-16 | 3.76E-21 | -16.0844 | 36.81713 | -4.04757 | KRT23 |
| 1553328_a_at | 4.02E-12 | 6.61E-16 | -11.7981 | 25.6721 | -4.04715 | SLC18A2 |
| 231096_at | 9.56E-03 | 5.84E-04 | -3.67854 | -0.59843 | -4.0057 | PCAT4 |
| 212236_x_at | 1.56E-10 | 7.13E-14 | -10.3259 | 21.24474 | -3.98988 | JUP |
| 233993_at | 2.67E-04 | 5.31E-06 | -5.11124 | 3.858562 | -3.93305 | MUC3 |
| 214234_s_at | 1.49E-14 | 8.17E-19 | -14.0758 | 31.90955 | -3.87349 | CYP3A5 |
| 233072_at | 6.71E-11 | 2.33E-14 | -10.6696 | 22.30589 | -3.84059 | NTNG2 |
| 204712_at | 3.49E-06 | 2.04E-08 | -6.68854 | 9.199876 | -3.80737 | WIF1 |
| 203435_s_at | 2.18E-07 | 6.21E-10 | -7.67167 | 12.56218 | -3.65217 | MME |
| 211002_s_at | 4.85E-09 | 4.97E-12 | -9.05824 | 17.19599 | -3.64361 | TRIM29 |
| 211401_s_at | 3.30E-11 | 9.67E-15 | -10.9438 | 23.14051 | -3.59018 | FGFR2 |
| 229735_s_at | 2.82E-07 | 8.57E-10 | -7.58079 | 12.25302 | -3.5592 | NIPAL3 |
| 212171_x_at | 1.51E-07 | 4.01E-10 | -7.79537 | 12.9822 | -3.55527 | VEGFA |
| 211834_s_at | 4.82E-11 | 1.50E-14 | -10.8071 | 22.72573 | -3.54587 | TP63 |
| 203638_s_at | 8.25E-10 | 6.03E-13 | -9.68143 | 19.21206 | -3.52202 | FGFR2 |
| 232929_at | 6.82E-09 | 7.61E-12 | -8.93387 | 16.78813 | -3.49401 | ZBTB20 |
| 211527_x_at | 6.06E-08 | 1.19E-10 | -8.14222 | 14.15402 | -3.4888 | VEGFA |
| 210513_s_at | 2.09E-07 | 5.85E-10 | -7.68866 | 12.61996 | -3.47884 | VEGFA |
| 225207_at | 4.60E-06 | 2.91E-08 | -6.58946 | 8.860095 | -3.46972 | PDK4 |
| 235019_at | 4.16E-05 | 4.79E-07 | -5.80059 | 6.166495 | -3.46806 | CPM |
| 1555942_a_at | 2.60E-08 | 3.79E-11 | -8.46927 | 15.2497 | -3.44643 | MIR205 |
| 205960_at | 7.14E-09 | 8.23E-12 | -8.91114 | 16.7134 | -3.42733 | PDK4 |
| 218332_at | 1.59E-07 | 4.26E-10 | -7.77879 | 12.92595 | -3.37523 | BEX1 |
| 226755_at | 4.37E-13 | 5.59E-17 | -12.6125 | 27.98743 | -3.34821 | MIR205HG |
| 214460_at | 2.57E-06 | 1.36E-08 | -6.80234 | 9.590218 | -3.30397 | LSAMP |
| 213992_at | 6.78E-08 | 1.38E-10 | -8.09948 | 14.01013 | -3.30303 | COL4A6 |
| 206032_at | 8.79E-05 | 1.30E-06 | -5.51664 | 5.208253 | -3.25501 | DSC3 |
| 219195_at | 9.75E-11 | 4.10E-14 | -10.4956 | 21.77054 | -3.24819 | PPARGC1A |
| 204537_s_at | 1.23E-07 | 2.84E-10 | -7.89319 | 13.31359 | -3.22072 | GABRE |
| 239860_at | 1.83E-08 | 2.54E-11 | -8.58445 | 15.63318 | -3.19627 | LOC100130232 |
| 222927_s_at | 7.46E-08 | 1.54E-10 | -8.06738 | 13.90198 | -3.19397 | CPLX3 |
| 204268_at | 7.79E-05 | 1.10E-06 | -5.56452 | 5.369175 | -3.18011 | S100A2 |
| 214235_at | 6.14E-10 | 3.93E-13 | -9.8092 | 19.61939 | -3.17267 | CYP3A5 |
| 205551_at | 8.25E-10 | 6.19E-13 | -9.67358 | 19.18696 | -3.15699 | SV2B |
| 212732_at | 7.27E-10 | 5.05E-13 | -9.73436 | 19.38106 | -3.09182 | MEG3 |
| 210512_s_at | 4.11E-07 | 1.41E-09 | -7.43936 | 11.77094 | -3.06372 | VEGFA |
| 213068_at | 1.77E-06 | 8.69E-09 | -6.92872 | 10.02362 | -3.06115 | DPT |
| 203559_s_at | 3.84E-07 | 1.26E-09 | -7.47228 | 11.88325 | -3.05401 | AOC1 |
| 239111_at | 5.48E-08 | 1.02E-10 | -8.18477 | 14.2971 | -3.03018 | PRDM8 |
| 238632_at | 2.14E-07 | 6.06E-10 | -7.67892 | 12.58685 | -3.01543 | RP11-44F21.5 |
| 239237_at | 3.43E-04 | 7.43E-06 | -5.0137 | 3.537954 | -3.00848 | TRG-AS1 |
| 203892_at | 1.46E-07 | 3.76E-10 | -7.81378 | 13.04462 | -3.00243 | WFDC2 |
| 215692_s_at | 4.10E-04 | 9.30E-06 | -4.94819 | 3.323642 | -2.99997 | MPPED2 |
| 211753_s_at | 7.98E-02 | 1.19E-02 | -2.61312 | -3.36445 | -2.99422 | RLN1 |
| 235111_at | 7.12E-09 | 8.07E-12 | -8.91665 | 16.73154 | -2.98539 | LSAMP |
| 241193_at | 1.73E-05 | 1.52E-07 | -6.12394 | 7.266631 | -2.98385 | ETS2 |
| 1552463_at | 2.41E-03 | 9.28E-05 | -4.25979 | 1.13389 | -2.97481 | SERPINB11 |
| 225875_s_at | 5.68E-07 | 2.06E-09 | -7.33368 | 11.41011 | -2.96225 | NIPAL3 |
| 217156_at | 1.51E-04 | 2.58E-06 | -5.32001 | 4.550475 | -2.93085 | HADHAP1 |
| 205692_s_at | 4.13E-04 | 9.40E-06 | -4.94496 | 3.313101 | -2.90156 | CD38 |
| 219775_s_at | 3.42E-08 | 5.44E-11 | -8.365 | 14.90144 | -2.89091 | CPLX3 |
| 1565034_s_at | 1.92E-03 | 6.96E-05 | -4.34804 | 1.407296 | -2.88724 | AFF3 |
| 226933_s_at | 7.63E-05 | 1.06E-06 | -5.57424 | 5.401909 | -2.88608 | ID4 |
| 214579_at | 3.91E-07 | 1.29E-09 | -7.46617 | 11.86242 | -2.88326 | NIPAL3 |
| 211020_at | 3.84E-07 | 1.25E-09 | -7.47432 | 11.8902 | -2.88098 | GCNT2 |
| 207781_s_at | 2.84E-10 | 1.51E-13 | -10.098 | 20.53221 | -2.87866 | ZNF711 |
| 204989_s_at | 6.39E-09 | 7.01E-12 | -8.9578 | 16.86675 | -2.87578 | ITGB4 |
| 232332_at | 5.82E-05 | 7.47E-07 | -5.67425 | 5.739046 | -2.87384 | KIAA1210 |
| 209351_at | 5.29E-10 | 3.29E-13 | -9.86279 | 19.78961 | -2.86435 | KRT14 |
| 237248_at | 6.80E-05 | 9.22E-07 | -5.61442 | 5.537228 | -2.84695 | PDE11A |
| 205082_s_at | 1.75E-10 | 8.30E-14 | -10.2795 | 21.10036 | -2.84404 | AOX1 |
| 213071_at | 2.94E-06 | 1.66E-08 | -6.74773 | 9.402916 | -2.83892 | DPT |
| 208228_s_at | 5.76E-09 | 6.01E-12 | -9.00286 | 17.01461 | -2.8019 | FGFR2 |
| 227819_at | 2.52E-06 | 1.33E-08 | -6.80929 | 9.614048 | -2.79931 | LGR6 |
| 224224_s_at | 8.67E-05 | 1.27E-06 | -5.52289 | 5.229234 | -2.78722 | PDE11A |
| 219597_s_at | 5.24E-07 | 1.88E-09 | -7.35923 | 11.4974 | -2.77069 | DUOX1 |
| 209747_at | 1.38E-06 | 6.29E-09 | -7.01961 | 10.33519 | -2.7701 | TGFB3 |
| 228218_at | 3.39E-08 | 5.33E-11 | -8.37132 | 14.92259 | -2.75242 | LSAMP |
| 205470_s_at | 1.18E-02 | 7.79E-04 | -3.58396 | -0.86748 | -2.73549 | KLK11 |
| 1563900_at | 1.67E-08 | 2.29E-11 | -8.61455 | 15.73319 | -2.73193 | FAM83B |
| 225806_at | 8.74E-10 | 6.87E-13 | -9.64245 | 19.08737 | -2.66883 | AJUBA |
| 235706_at | 2.95E-05 | 3.11E-07 | -5.92288 | 6.581646 | -2.64969 | CPM |
| 204457_s_at | 4.46E-06 | 2.78E-08 | -6.60183 | 8.902502 | -2.62365 | GAS1 |
| 223890_at | 3.38E-08 | 5.25E-11 | -8.37524 | 14.9357 | -2.61746 | PRO1082 |
| 219864_s_at | 1.01E-05 | 7.83E-08 | -6.31129 | 7.907007 | -2.60152 | RCAN3 |
| 58916_at | 8.83E-10 | 7.27E-13 | -9.62571 | 19.03376 | -2.59939 | KCTD14 |
| 215889_at | 2.00E-05 | 1.87E-07 | -6.06564 | 7.067736 | -2.59891 | SKIL |
| 203849_s_at | 1.36E-06 | 6.14E-09 | -7.02633 | 10.3582 | -2.58609 | KIF1A |
| 219197_s_at | 9.08E-03 | 5.46E-04 | -3.70063 | -0.535 | -2.58442 | SCUBE2 |
| 219498_s_at | 4.61E-07 | 1.61E-09 | -7.40246 | 11.64501 | -2.58203 | BCL11A |
| 216298_at | 1.17E-04 | 1.85E-06 | -5.41573 | 4.869998 | -2.5777 | TARP |
| 1558430_at | 1.49E-05 | 1.28E-07 | -6.17266 | 7.43301 | -2.57293 | RP11-680G24.5 |
| 205725_at | 1.12E-02 | 7.27E-04 | -3.60675 | -0.80302 | -2.56345 | SCGB1A1 |
| 241765_at | 1.61E-05 | 1.40E-07 | -6.1477 | 7.347773 | -2.55083 | CPM |
| 230788_at | 1.38E-06 | 6.29E-09 | -7.0192 | 10.33377 | -2.54855 | GCNT2 |
| 202888_s_at | 6.49E-02 | 8.56E-03 | -2.73975 | -3.06967 | -2.54688 | MAP7 |
| 235651_at | 1.24E-04 | 1.98E-06 | -5.39544 | 4.802146 | -2.54648 | TTC22 |
| 222357_at | 2.08E-08 | 2.92E-11 | -8.54394 | 15.49848 | -2.5422 | ZBTB20 |
| 216598_s_at | 1.33E-03 | 4.32E-05 | -4.49266 | 1.860424 | -2.53851 | CCL2 |
| 220298_s_at | 5.76E-08 | 1.09E-10 | -8.16743 | 14.23882 | -2.51736 | SPATA6 |
| 218717_s_at | 6.53E-04 | 1.69E-05 | -4.77214 | 2.752261 | -2.51638 | LEPREL1 |
| 1555719_a_at | 9.54E-07 | 3.89E-09 | -7.15448 | 10.79716 | -2.5129 | MAATS1 |
| 1552511_a_at | 3.00E-07 | 9.27E-10 | -7.55852 | 12.1772 | -2.51265 | CPA6 |
| 227848_at | 2.96E-04 | 6.02E-06 | -5.07483 | 3.738683 | -2.51078 | PEBP4 |
| 204418_x_at | 4.78E-06 | 3.08E-08 | -6.57353 | 8.805446 | -2.50767 | GSTM2 |
| 206100_at | 6.45E-04 | 1.66E-05 | -4.77754 | 2.769692 | -2.50497 | CPM |
| 223302_s_at | 2.42E-04 | 4.68E-06 | -5.14779 | 3.979177 | -2.50287 | ZNF655 |
| 208399_s_at | 2.20E-06 | 1.14E-08 | -6.85225 | 9.761389 | -2.49894 | EDN3 |
| 217557_s_at | 5.84E-04 | 1.46E-05 | -4.81569 | 2.892965 | -2.49174 | CPM |
| 201850_at | 5.99E-08 | 1.15E-10 | -8.15146 | 14.1851 | -2.47 | CAPG |
| 210096_at | 2.31E-05 | 2.25E-07 | -6.01388 | 6.891328 | -2.46544 | CYP4B1 |
| 219250_s_at | 6.06E-08 | 1.18E-10 | -8.1433 | 14.15765 | -2.46507 | FLRT3 |
| 207938_at | 9.65E-03 | 5.92E-04 | -3.67426 | -0.61068 | -2.46466 | PI15 |
| 211864_s_at | 1.01E-07 | 2.22E-10 | -7.96325 | 13.55052 | -2.46108 | MYOF |
| 1554789_a_at | 7.15E-05 | 9.82E-07 | -5.59633 | 5.476279 | -2.44811 | PDE8B |
| 206714_at | 9.62E-02 | 1.60E-02 | -2.49501 | -3.62993 | -2.44688 | ALOX15B |
| 203639_s_at | 6.13E-08 | 1.21E-10 | -8.13635 | 14.13428 | -2.44324 | FGFR2 |
| 219735_s_at | 4.28E-04 | 9.82E-06 | -4.93215 | 3.27133 | -2.43563 | TFCP2L1 |
| 217054_at | 2.87E-03 | 1.17E-04 | -4.18912 | 0.916728 | -2.43026 | AK096803 |
| 1557706_at | 1.18E-06 | 5.13E-09 | -7.07681 | 10.53117 | -2.41707 | ZHX2 |
| 226210_s_at | 2.77E-05 | 2.85E-07 | -5.94773 | 6.666146 | -2.41457 | MEG3 |
| 209392_at | 2.68E-05 | 2.72E-07 | -5.96018 | 6.708519 | -2.41096 | ENPP2 |
| 207016_s_at | 2.48E-05 | 2.45E-07 | -5.98994 | 6.809799 | -2.40905 | ALDH1A2 |
| 243322_at | 4.14E-07 | 1.43E-09 | -7.43575 | 11.75864 | -2.39914 | ZFHX3 |
| 210102_at | 2.52E-11 | 6.45E-15 | -11.0707 | 23.52298 | -2.39612 | VWA5A |
| 229244_at | 1.01E-08 | 1.24E-11 | -8.79169 | 16.31977 | -2.38873 | LSAMP |
| 229400_at | 9.59E-04 | 2.80E-05 | -4.62271 | 2.272925 | -2.38312 | HOXD10 |
| 205827_at | 1.96E-05 | 1.82E-07 | -6.07334 | 7.094011 | -2.37862 | CCK |
| 222853_at | 4.78E-10 | 2.89E-13 | -9.90211 | 19.91427 | -2.37057 | FLRT3 |
| 209570_s_at | 1.45E-05 | 1.23E-07 | -6.18415 | 7.472261 | -2.36965 | NSG1 |
| 203632_s_at | 1.07E-08 | 1.37E-11 | -8.76357 | 16.22686 | -2.36956 | GPRC5B |
| 208078_s_at | 4.85E-03 | 2.33E-04 | -3.97297 | 0.263223 | -2.36641 | SIK1 |
| 1570393_at | 1.31E-04 | 2.14E-06 | -5.37349 | 4.728839 | -2.36404 | EML5 |
| 204430_s_at | 4.54E-08 | 8.13E-11 | -8.24997 | 14.51608 | -2.35373 | SLC2A5 |
| 209604_s_at | 1.41E-05 | 1.19E-07 | -6.19271 | 7.501505 | -2.33854 | GATA3 |
| 207547_s_at | 9.79E-07 | 4.08E-09 | -7.14084 | 10.75047 | -2.33792 | FAM107A |
| 206132_at | 8.34E-07 | 3.33E-09 | -7.19841 | 10.94753 | -2.33386 | MCC |
| 213139_at | 9.98E-10 | 8.44E-13 | -9.58111 | 18.89074 | -2.3273 | SNAI2 |
| 233882_s_at | 4.02E-03 | 1.82E-04 | -4.05088 | 0.496844 | -2.32644 | SEMA6D |
| 208022_s_at | 1.73E-06 | 8.23E-09 | -6.94384 | 10.07545 | -2.325 | CDC14B |
| 209343_at | 8.66E-05 | 1.27E-06 | -5.52391 | 5.232651 | -2.32323 | EFHD1 |
| 202987_at | 1.06E-08 | 1.34E-11 | -8.76945 | 16.24631 | -2.31257 | TRAF3IP2 |
| 210297_s_at | 8.11E-05 | 1.16E-06 | -5.54898 | 5.316919 | -2.31143 | MSMB |
| 221898_at | 5.12E-08 | 9.45E-11 | -8.20698 | 14.37173 | -2.31029 | PDPN |
| 238584_at | 4.53E-12 | 8.29E-16 | -11.7248 | 25.45894 | -2.30959 | IQCA1 |
| 227475_at | 1.00E-03 | 2.99E-05 | -4.60312 | 2.210493 | -2.30846 | FOXQ1 |
| 1565716_at | 1.19E-03 | 3.71E-05 | -4.53855 | 2.005443 | -2.3084 | FUS |
| 217600_at | 1.04E-05 | 8.09E-08 | -6.30227 | 7.876148 | -2.30736 | SCUBE3 |
| 231527_at | 1.12E-03 | 3.44E-05 | -4.56092 | 2.076377 | -2.30306 | FLJ36840 |
| 226272_at | 1.00E-04 | 1.53E-06 | -5.46976 | 5.050942 | -2.30114 | RCAN3 |
| 238847_at | 1.75E-06 | 8.37E-09 | -6.93925 | 10.05971 | -2.29979 | HOXD10 |
| 215333_x_at | 2.59E-05 | 2.59E-07 | -5.97423 | 6.756341 | -2.29961 | GSTM1 |
| 215025_at | 3.66E-07 | 1.17E-09 | -7.49209 | 11.95081 | -2.28887 | NTRK3 |
| 230398_at | 2.38E-07 | 7.00E-10 | -7.63791 | 12.44739 | -2.28755 | TNS4 |
| 1560369_at | 2.02E-04 | 3.71E-06 | -5.21559 | 4.203489 | -2.27641 | ANKH |
| 1554980_a_at | 4.56E-02 | 5.11E-03 | -2.93229 | -2.60229 | -2.27142 | ATF3 |
| 203349_s_at | 2.71E-07 | 8.09E-10 | -7.59716 | 12.30876 | -2.2708 | ETV5 |
| 230102_at | 7.80E-08 | 1.63E-10 | -8.05069 | 13.84571 | -2.26477 | ETV5 |
| 202949_s_at | 6.88E-06 | 4.99E-08 | -6.43779 | 8.340193 | -2.26176 | FHL2 |
| 209291_at | 1.00E-05 | 7.71E-08 | -6.3158 | 7.922443 | -2.25905 | ID4 |
| 1562269_at | 2.96E-07 | 9.10E-10 | -7.56379 | 12.19513 | -2.25844 | RP11-203B7.2 |
| 222744_s_at | 9.98E-10 | 8.58E-13 | -9.57626 | 18.87518 | -2.2573 | TMLHE |
| 213791_at | 8.09E-04 | 2.25E-05 | -4.68717 | 2.47904 | -2.25605 | PENK |
| 205608_s_at | 9.55E-07 | 3.93E-09 | -7.15172 | 10.78771 | -2.25559 | ANGPT1 |
| 1556190_s_at | 1.24E-04 | 2.00E-06 | -5.39315 | 4.794514 | -2.23474 | LOC100507516 |
| 221555_x_at | 5.72E-06 | 3.87E-08 | -6.5091 | 8.584578 | -2.23453 | CDC14B |
| 233891_at | 4.17E-03 | 1.91E-04 | -4.03588 | 0.451699 | -2.23115 | MUC3 |
| 204550_x_at | 1.73E-05 | 1.53E-07 | -6.12284 | 7.262891 | -2.22497 | GSTM1 |
| 210273_at | 1.67E-04 | 2.93E-06 | -5.28349 | 4.428937 | -2.21952 | PCDH7 |
| 202672_s_at | 8.76E-02 | 1.38E-02 | -2.55521 | -3.49579 | -2.21885 | ATF3 |
| 241631_at | 3.73E-08 | 6.06E-11 | -8.33413 | 14.79813 | -2.21375 | ARHGEF40 |
| 205882_x_at | 2.69E-06 | 1.45E-08 | -6.78477 | 9.529953 | -2.20753 | ADD3 |
| 1555497_a_at | 1.99E-04 | 3.61E-06 | -5.22286 | 4.227606 | -2.20511 | CYP4B1 |
| 235494_at | 1.82E-06 | 9.01E-09 | -6.91864 | 9.989047 | -2.20143 | LSAMP |
| 214091_s_at | 3.91E-08 | 6.65E-11 | -8.30748 | 14.70888 | -2.19828 | GPX3 |
| 209283_at | 1.24E-07 | 2.87E-10 | -7.89057 | 13.30472 | -2.19704 | CRYAB |
| 240211_at | 2.04E-07 | 5.67E-10 | -7.69742 | 12.64972 | -2.19574 | APCDD1 |
| 206211_at | 1.98E-02 | 1.56E-03 | -3.3514 | -1.51158 | -2.19471 | SELE |
| 218631_at | 1.13E-08 | 1.46E-11 | -8.74404 | 16.1623 | -2.19348 | AVPI1 |
| 1553764_a_at | 1.61E-08 | 2.18E-11 | -8.62807 | 15.77806 | -2.19199 | AJUBA |
| 235077_at | 8.25E-10 | 6.12E-13 | -9.67708 | 19.19815 | -2.19015 | MEG3 |
| 208084_at | 6.82E-07 | 2.54E-09 | -7.27384 | 11.20555 | -2.18544 | ITGB6 |
| 1569020_at | 3.03E-05 | 3.22E-07 | -5.91292 | 6.547808 | -2.17987 | NEDD9 |
| 226225_at | 2.79E-06 | 1.54E-08 | -6.7688 | 9.475174 | -2.17479 | MCC |
| 1555809_at | 2.92E-05 | 3.08E-07 | -5.9253 | 6.589881 | -2.17347 | CRISPLD2 |
| 219616_at | 7.24E-06 | 5.30E-08 | -6.42114 | 8.283142 | -2.1731 | ACSS3 |
| 225876_at | 1.06E-05 | 8.39E-08 | -6.29206 | 7.841209 | -2.17076 | NIPAL3 |
| 211276_at | 3.16E-04 | 6.59E-06 | -5.04843 | 3.651909 | -2.16602 | TCEAL2 |
| 225046_at | 7.16E-06 | 5.23E-08 | -6.42493 | 8.296136 | -2.16542 | LOC102724441 |
| 238459_x_at | 4.83E-05 | 5.80E-07 | -5.74635 | 5.982802 | -2.16434 | SPATA6 |
| 206237_s_at | 6.00E-09 | 6.48E-12 | -8.98094 | 16.94271 | -2.16326 | NRG1 |
| 206065_s_at | 1.38E-06 | 6.31E-09 | -7.01845 | 10.33121 | -2.16264 | DPYS |
| 213520_at | 4.73E-05 | 5.65E-07 | -5.75354 | 6.007121 | -2.15729 | RECQL4 |
| 226599_at | 1.63E-04 | 2.85E-06 | -5.29166 | 4.456088 | -2.15379 | FHDC1 |
| 1565786_x_at | 2.68E-05 | 2.73E-07 | -5.95967 | 6.706777 | -2.153 | FLJ45482 |
| 1560834_a_at | 3.59E-05 | 4.00E-07 | -5.85184 | 6.340323 | -2.14525 | RMST |
| 218886_at | 1.50E-07 | 3.92E-10 | -7.80229 | 13.00568 | -2.14209 | PAK1IP1 |
| 1559950_at | 1.07E-06 | 4.56E-09 | -7.11001 | 10.64489 | -2.14048 | FAM66B |
| 226420_at | 1.29E-03 | 4.09E-05 | -4.50865 | 1.910894 | -2.13821 | MECOM |
| 232404_at | 6.82E-07 | 2.53E-09 | -7.27537 | 11.21078 | -2.13787 | SHROOM4 |
| 207430_s_at | 2.40E-04 | 4.63E-06 | -5.15132 | 3.990841 | -2.13442 | MSMB |
| 231998_at | 7.55E-06 | 5.54E-08 | -6.4088 | 8.240872 | -2.13077 | SART1 |
| 222835_at | 3.75E-06 | 2.24E-08 | -6.66307 | 9.112509 | -2.12659 | THSD4 |
| 209737_at | 1.81E-05 | 1.62E-07 | -6.10699 | 7.208813 | -2.1233 | MAGI2 |
| 208083_s_at | 4.92E-06 | 3.21E-08 | -6.56154 | 8.764346 | -2.11924 | ITGB6 |
| 219497_s_at | 7.79E-05 | 1.10E-06 | -5.56399 | 5.367409 | -2.11356 | BCL11A |
| 1569141_a_at | 1.45E-07 | 3.72E-10 | -7.81725 | 13.0564 | -2.1132 | PPARGC1A |
| 1559952_x_at | 8.12E-07 | 3.21E-09 | -7.20879 | 10.98306 | -2.11254 | FAM66B |
| 215078_at | 7.55E-03 | 4.25E-04 | -3.7819 | -0.29995 | -2.1108 | LOC100129518 |
| 216880_at | 5.11E-06 | 3.38E-08 | -6.54711 | 8.71486 | -2.09952 | RAD51B |
| 243617_at | 1.44E-06 | 6.67E-09 | -7.00275 | 10.27741 | -2.09945 | ZNF827 |
| 203708_at | 5.05E-03 | 2.46E-04 | -3.95691 | 0.215325 | -2.09767 | PDE4B |
| 1558569_at | 1.66E-03 | 5.76E-05 | -4.40526 | 1.585855 | -2.09519 | LOC100131541 |
| 209292_at | 7.00E-06 | 5.10E-08 | -6.43204 | 8.320489 | -2.09323 | ID4 |
| 1565666_s_at | 1.78E-01 | 4.95E-02 | -2.01478 | -4.60805 | -2.08903 | MUC6 |
| 1555052_a_at | 2.78E-05 | 2.88E-07 | -5.94439 | 6.654798 | -2.08637 | SYT9 |
| 41660_at | 2.82E-06 | 1.56E-08 | -6.76481 | 9.461488 | -2.08476 | CELSR1 |
| 238050_at | 1.64E-03 | 5.68E-05 | -4.40961 | 1.599465 | -2.08467 | ANTXR2 |
| 211846_s_at | 3.66E-06 | 2.17E-08 | -6.67138 | 9.141011 | -2.08096 | PVRL1 |
| 210360_s_at | 1.02E-04 | 1.57E-06 | -5.46208 | 5.025181 | -2.07768 | MTSS1 |
| 227702_at | 1.98E-04 | 3.61E-06 | -5.22349 | 4.229699 | -2.07703 | CYP4X1 |
| 226535_at | 3.84E-06 | 2.34E-08 | -6.65093 | 9.070897 | -2.07338 | ITGB6 |
| 225809_at | 2.68E-06 | 1.43E-08 | -6.78846 | 9.542603 | -2.07068 | PARM1 |
| 238933_at | 1.68E-04 | 2.95E-06 | -5.28095 | 4.420461 | -2.06607 | IRS1 |
| 1569607_s_at | 1.70E-02 | 1.27E-03 | -3.42206 | -1.31861 | -2.06525 | ANKRD20A1 |
| 225511_at | 2.70E-10 | 1.38E-13 | -10.124 | 20.61405 | -2.05407 | GPRC5B |
| 1556839_s_at | 7.80E-08 | 1.64E-10 | -8.04972 | 13.84243 | -2.05146 | LOC100289090 |
| 204687_at | 1.42E-07 | 3.57E-10 | -7.82863 | 13.09495 | -2.0514 | PARM1 |
| 225293_at | 2.59E-05 | 2.57E-07 | -5.97636 | 6.763578 | -2.04801 | COL27A1 |
| 206938_at | 6.49E-05 | 8.71E-07 | -5.6305 | 5.591434 | -2.04197 | SRD5A2 |
| 226470_at | 1.28E-05 | 1.05E-07 | -6.22776 | 7.621313 | -2.04178 | GGT7 |
| 1555536_at | 3.85E-05 | 4.37E-07 | -5.82651 | 6.25439 | -2.04 | ANTXR2 |
| 218694_at | 3.26E-04 | 6.95E-06 | -5.03314 | 3.601707 | -2.03605 | ARMCX1 |
| 37005_at | 1.43E-06 | 6.60E-09 | -7.00601 | 10.28858 | -2.03605 | MINOS1-NBL1 |
| 205535_s_at | 1.94E-05 | 1.80E-07 | -6.07744 | 7.107996 | -2.03501 | PCDH7 |
| 227390_at | 5.31E-05 | 6.59E-07 | -5.7099 | 5.859513 | -2.03453 | MEG3 |
| 227697_at | 1.70E-02 | 1.27E-03 | -3.42169 | -1.31962 | -2.0314 | SOCS3 |
| 214324_at | 1.20E-01 | 2.37E-02 | -2.33436 | -3.97574 | -2.03094 | GP2 |
| 241382_at | 4.37E-05 | 5.07E-07 | -5.78439 | 6.111596 | -2.02886 | PCP4L1 |
| 201752_s_at | 3.76E-07 | 1.22E-09 | -7.48165 | 11.91521 | -2.02526 | ADD3 |
| 226281_at | 2.00E-05 | 1.88E-07 | -6.06533 | 7.066686 | -2.02223 | DNER |
| 212187_x_at | 2.77E-03 | 1.11E-04 | -4.20449 | 0.96381 | -2.02175 | PTGDS |
| 229947_at | 2.11E-02 | 1.70E-03 | -3.32092 | -1.59402 | -2.01902 | PI15 |
| 235055_x_at | 1.31E-02 | 9.00E-04 | -3.53631 | -1.00154 | -2.01823 | MUC4 |
| 234710_s_at | 5.85E-04 | 1.47E-05 | -4.81437 | 2.888709 | -2.01551 | PARP6 |
| 1560370_x_at | 9.85E-04 | 2.91E-05 | -4.61109 | 2.235869 | -2.00967 | ANKH |
| 218000_s_at | 7.79E-05 | 1.10E-06 | -5.56403 | 5.367523 | -2.00871 | PHLDA1 |
| 1554726_at | 3.29E-04 | 7.02E-06 | -5.03001 | 3.591427 | -2.00797 | ZNF655 |
| 201798_s_at | 2.79E-05 | 2.90E-07 | -5.94199 | 6.646633 | -2.00622 | MYOF |
| 237449_at | 9.15E-03 | 5.51E-04 | -3.69739 | -0.54434 | -2.006 | SP8 |
| 203323_at | 2.10E-07 | 5.91E-10 | -7.68579 | 12.61021 | -2.00592 | CAV2 |
| 229124_at | 5.04E-05 | 6.13E-07 | -5.73037 | 5.928719 | -2.00138 | PROK1 |
| 204855_at | 7.64E-05 | 1.06E-06 | -5.57339 | 5.399041 | -1.9963 | SERPINB5 |
| 1570190_at | 5.73E-07 | 2.09E-09 | -7.32991 | 11.39722 | -1.99307 | AK311120 |
| 1554997_a_at | 1.59E-02 | 1.16E-03 | -3.45182 | -1.23662 | -1.99259 | PTGS2 |
| 229064_s_at | 5.67E-04 | 1.40E-05 | -4.82768 | 2.93177 | -1.98755 | RCAN3 |
| 213787_s_at | 2.47E-03 | 9.56E-05 | -4.25081 | 1.106201 | -1.98604 | EBP |
| 218844_at | 2.23E-07 | 6.44E-10 | -7.6614 | 12.5273 | -1.98028 | ACSF2 |
| 218350_s_at | 7.90E-03 | 4.51E-04 | -3.76239 | -0.35665 | -1.97601 | GMNN |
| 214279_s_at | 1.51E-06 | 7.06E-09 | -6.98692 | 10.22316 | -1.97356 | NDRG2 |
| 203413_at | 1.02E-02 | 6.35E-04 | -3.65123 | -0.67652 | -1.97147 | NELL2 |
| 208962_s_at | 9.08E-05 | 1.35E-06 | -5.5056 | 5.171152 | -1.96346 | FADS1 |
| 206069_s_at | 8.95E-04 | 2.56E-05 | -4.6487 | 2.355902 | -1.96252 | ACADL |
| 208963_x_at | 4.04E-07 | 1.35E-09 | -7.45216 | 11.81463 | -1.96248 | FADS1 |
| 239163_at | 4.04E-05 | 4.61E-07 | -5.81151 | 6.20352 | -1.9578 | UBE2B |
| 210267_at | 7.96E-07 | 3.13E-09 | -7.21574 | 11.00682 | -1.95049 | NIPAL3 |
| 207480_s_at | 1.73E-05 | 1.53E-07 | -6.12211 | 7.260414 | -1.94826 | MEIS2 |
| 227210_at | 3.66E-07 | 1.17E-09 | -7.49344 | 11.95543 | -1.94813 | SFMBT2 |
| 242546_at | 2.67E-02 | 2.38E-03 | -3.20481 | -1.90384 | -1.94798 | DUXAP10 |
| 204287_at | 1.35E-07 | 3.25E-10 | -7.85525 | 13.18513 | -1.94741 | SYNGR1 |
| 219747_at | 4.21E-03 | 1.94E-04 | -4.03148 | 0.438482 | -1.94639 | NDNF |
| 227554_at | 1.29E-07 | 3.08E-10 | -7.87038 | 13.23638 | -1.94617 | MAGI2-AS3 |
| 237027_at | 4.05E-06 | 2.48E-08 | -6.63385 | 9.012299 | -1.94409 | LSAMP-AS1 |
| 222891_s_at | 3.11E-04 | 6.45E-06 | -5.0547 | 3.672495 | -1.94332 | BCL11A |
| 213503_x_at | 6.93E-07 | 2.60E-09 | -7.268 | 11.1856 | -1.94319 | ANXA2 |
| 241124_at | 1.76E-06 | 8.62E-09 | -6.93106 | 10.03165 | -1.94095 | LOC101928702 |
| 228421_s_at | 3.97E-04 | 8.89E-06 | -4.96114 | 3.365946 | -1.93536 | EFEMP1 |
| 228640_at | 1.43E-03 | 4.72E-05 | -4.46557 | 1.77508 | -1.93371 | PCDH7 |
| 209616_s_at | 1.68E-03 | 5.88E-05 | -4.39922 | 1.566935 | -1.93111 | CES1 |
| 1560119_at | 1.63E-04 | 2.83E-06 | -5.29352 | 4.462285 | -1.93046 | LINC00937 |
| 201590_x_at | 2.18E-06 | 1.12E-08 | -6.85763 | 9.779849 | -1.93021 | ANXA2 |
| 210427_x_at | 8.78E-07 | 3.57E-09 | -7.17886 | 10.88061 | -1.92677 | ANXA2 |
| 219545_at | 1.94E-05 | 1.80E-07 | -6.07683 | 7.105899 | -1.92629 | KCTD14 |
| 225062_at | 2.86E-05 | 3.00E-07 | -5.93256 | 6.614552 | -1.92461 | LOC102724441 |
| 234605_at | 1.84E-06 | 9.22E-09 | -6.91213 | 9.966738 | -1.92279 | CDC14B |
| 226638_at | 2.25E-04 | 4.23E-06 | -5.17753 | 4.077489 | -1.92263 | ARHGAP23 |
| 207382_at | 7.80E-08 | 1.65E-10 | -8.04735 | 13.83443 | -1.92089 | TP63 |
| 1570124_at | 4.45E-03 | 2.08E-04 | -4.00894 | 0.37082 | -1.91812 | SLC29A4 |
| 205857_at | 1.39E-07 | 3.46E-10 | -7.83779 | 13.12601 | -1.9144 | SLC18A2 |
| 243213_at | 8.33E-03 | 4.86E-04 | -3.73817 | -0.42678 | -1.91185 | ZBTB46 |
| 213274_s_at | 6.52E-06 | 4.62E-08 | -6.45938 | 8.414156 | -1.90888 | CTSB |
| 1555847_a_at | 3.69E-04 | 8.14E-06 | -4.98692 | 3.450245 | -1.90851 | LOC284454 |
| 208234_x_at | 4.11E-07 | 1.41E-09 | -7.4398 | 11.77246 | -1.90537 | FGFR2 |
| 230518_at | 1.43E-04 | 2.40E-06 | -5.34077 | 4.619665 | -1.90313 | MPZL2 |
| 210397_at | 9.42E-06 | 7.20E-08 | -6.33483 | 7.98759 | -1.90153 | DEFB1 |
| 242714_at | 1.38E-07 | 3.42E-10 | -7.84108 | 13.13716 | -1.89889 | LOC101928429 |
| 219153_s_at | 4.73E-05 | 5.63E-07 | -5.75467 | 6.010965 | -1.89862 | THSD4 |
| 235108_at | 3.72E-05 | 4.19E-07 | -5.83848 | 6.294995 | -1.89776 | KCNK3 |
| 1553787_at | 4.08E-07 | 1.39E-09 | -7.44471 | 11.78922 | -1.89551 | C11orf45 |
| 242414_at | 3.36E-06 | 1.95E-08 | -6.70159 | 9.244649 | -1.89517 | LOC101929880 |
| 31845_at | 3.22E-04 | 6.77E-06 | -5.04074 | 3.626663 | -1.89496 | ELF4 |
| 201348_at | 8.70E-05 | 1.28E-06 | -5.52137 | 5.224134 | -1.88983 | GPX3 |
| 1559988_at | 4.00E-07 | 1.32E-09 | -7.45816 | 11.83509 | -1.88724 | ZNF483 |
| 1553809_a_at | 1.23E-05 | 9.98E-08 | -6.24323 | 7.67421 | -1.88668 | TMEM252 |
| 209791_at | 3.22E-04 | 6.80E-06 | -5.03932 | 3.62198 | -1.88668 | PADI2 |
| 205041_s_at | 1.27E-01 | 2.62E-02 | -2.29248 | -4.06289 | -1.88641 | ORM1 |
| 244321_at | 1.11E-11 | 2.44E-15 | -11.379 | 24.44323 | -1.88632 | PGAP1 |
| 218330_s_at | 2.60E-08 | 3.80E-11 | -8.46828 | 15.2464 | -1.88529 | NAV2 |
| 213228_at | 1.77E-03 | 6.26E-05 | -4.38026 | 1.507709 | -1.88519 | PDE8B |
| 206089_at | 4.52E-05 | 5.31E-07 | -5.77136 | 6.067464 | -1.88286 | NELL1 |
| 230416_at | 4.17E-06 | 2.57E-08 | -6.62446 | 8.980106 | -1.88225 | PDZD8 |
| 209260_at | 7.05E-03 | 3.88E-04 | -3.81133 | -0.21416 | -1.87923 | SFN |
| 205979_at | 6.79E-03 | 3.68E-04 | -3.82839 | -0.16426 | -1.87761 | SCGB2A1 |
| 201286_at | 1.44E-02 | 1.02E-03 | -3.49411 | -1.11937 | -1.87709 | SDC1 |
| 220299_at | 6.03E-05 | 7.86E-07 | -5.65997 | 5.690835 | -1.87549 | SPATA6 |
| 204205_at | 8.44E-04 | 2.37E-05 | -4.67203 | 2.430528 | -1.87337 | APOBEC3G |
| 214823_at | 1.15E-04 | 1.81E-06 | -5.42182 | 4.890368 | -1.87174 | ZNF204P |
| 1567028_s_at | 5.82E-03 | 2.98E-04 | -3.8953 | 0.03255 | -1.87021 | SH3GL1P2 |
| 239466_at | 3.22E-04 | 6.79E-06 | -5.03977 | 3.623471 | -1.86947 | LINC00883 |
| 204748_at | 2.88E-02 | 2.65E-03 | -3.16747 | -2.00194 | -1.86753 | PTGS2 |
| 205382_s_at | 3.95E-04 | 8.81E-06 | -4.96384 | 3.374786 | -1.86612 | CFD |
| 210347_s_at | 1.04E-06 | 4.43E-09 | -7.11808 | 10.67253 | -1.86607 | BCL11A |
| 205735_s_at | 1.34E-02 | 9.19E-04 | -3.52928 | -1.02122 | -1.86257 | AFF3 |
| 230595_at | 4.00E-02 | 4.20E-03 | -3.00334 | -2.42422 | -1.86179 | PGM5-AS1 |
| 203256_at | 3.54E-05 | 3.89E-07 | -5.85914 | 6.365106 | -1.86152 | CDH3 |
| 201329_s_at | 1.72E-05 | 1.52E-07 | -6.12498 | 7.270212 | -1.86074 | ETS2 |
| 226304_at | 6.68E-07 | 2.47E-09 | -7.28226 | 11.23433 | -1.85797 | HSPB6 |
| 201621_at | 9.06E-06 | 6.84E-08 | -6.34941 | 8.037478 | -1.85673 | NBL1 |
| 232481_s_at | 2.48E-03 | 9.63E-05 | -4.2485 | 1.099097 | -1.85286 | SLITRK6 |
| 211748_x_at | 4.17E-03 | 1.91E-04 | -4.03532 | 0.450021 | -1.8507 | PTGDS |
| 204400_at | 2.57E-04 | 5.03E-06 | -5.12706 | 3.910757 | -1.84788 | EFS |
| 207291_at | 6.35E-05 | 8.48E-07 | -5.63821 | 5.617433 | -1.83922 | PRRG4 |
| 205011_at | 7.11E-05 | 9.70E-07 | -5.6 | 5.48863 | -1.83729 | VWA5A |
| 205141_at | 4.55E-04 | 1.06E-05 | -4.90943 | 3.197267 | -1.83475 | ANG |
| 1555416_a_at | 1.23E-01 | 2.46E-02 | -2.31968 | -4.00643 | -1.8314 | ALOX15B |
| 209074_s_at | 3.76E-07 | 1.22E-09 | -7.48134 | 11.91415 | -1.82461 | FAM107A |
| 243967_at | 2.08E-02 | 1.67E-03 | -3.3278 | -1.57547 | -1.82337 | FCRL1 |
| 205952_at | 1.67E-03 | 5.81E-05 | -4.40278 | 1.578082 | -1.81592 | KCNK3 |
| 202218_s_at | 4.46E-05 | 5.23E-07 | -5.77579 | 6.082463 | -1.8128 | FADS2 |
| 1566901_at | 6.23E-04 | 1.58E-05 | -4.79145 | 2.81459 | -1.8115 | TGIF1 |
| 203779_s_at | 8.65E-05 | 1.26E-06 | -5.52489 | 5.235949 | -1.8097 | MPZL2 |
| 1552703_s_at | 2.53E-04 | 4.92E-06 | -5.13343 | 3.931774 | -1.80738 | CARD16 |
| 225842_at | 6.12E-04 | 1.55E-05 | -4.79851 | 2.837424 | -1.80673 | PHLDA1 |
| 236534_at | 1.21E-04 | 1.91E-06 | -5.40584 | 4.836922 | -1.80628 | BNIPL |
| 242525_at | 6.41E-06 | 4.51E-08 | -6.46626 | 8.437755 | -1.80604 | SLC2A5 |
| 228698_at | 1.76E-06 | 8.47E-09 | -6.93597 | 10.04849 | -1.8058 | SOX7 |
| 218807_at | 1.68E-03 | 5.89E-05 | -4.39883 | 1.565716 | -1.80356 | VAV3 |
| 205724_at | 8.54E-03 | 5.01E-04 | -3.72829 | -0.45532 | -1.80149 | PKP1 |
| 213789_at | 3.33E-03 | 1.42E-04 | -4.12919 | 0.733892 | -1.80017 | EBP |
| 209959_at | 8.58E-02 | 1.33E-02 | -2.56833 | -3.46622 | -1.79572 | NR4A3 |
| 209318_x_at | 2.46E-05 | 2.43E-07 | -5.99264 | 6.819006 | -1.79387 | PLAGL1 |
| 235945_at | 5.08E-04 | 1.22E-05 | -4.86748 | 3.060857 | -1.79074 | SRD5A2 |
| 222088_s_at | 1.10E-02 | 7.08E-04 | -3.61556 | -0.77803 | -1.79036 | SLC2A14 |
| 204607_at | 6.32E-02 | 8.24E-03 | -2.75442 | -3.03486 | -1.78951 | HMGCS2 |
| 228557_at | 4.04E-07 | 1.35E-09 | -7.45254 | 11.81591 | -1.78866 | L3MBTL4 |
| 1557535_at | 3.58E-03 | 1.56E-04 | -4.09894 | 0.642062 | -1.78363 | PALLD |
| 208161_s_at | 6.57E-05 | 8.84E-07 | -5.62636 | 5.577466 | -1.7825 | ABCC3 |
| 227961_at | 4.68E-08 | 8.48E-11 | -8.23799 | 14.47588 | -1.78187 | CTSB |
| 201328_at | 2.85E-05 | 2.98E-07 | -5.93439 | 6.620787 | -1.77918 | ETS2 |
| 204304_s_at | 8.01E-04 | 2.23E-05 | -4.69053 | 2.489803 | -1.7778 | PROM1 |
| 227519_at | 1.84E-02 | 1.42E-03 | -3.38313 | -1.42522 | -1.77044 | PLAC4 |
| 223315_at | 5.89E-05 | 7.61E-07 | -5.66918 | 5.721953 | -1.76325 | NTN4 |
| 231969_at | 5.83E-04 | 1.45E-05 | -4.81671 | 2.896266 | -1.76281 | STOX2 |
| 227642_at | 1.49E-04 | 2.53E-06 | -5.32516 | 4.567642 | -1.7565 | TFCP2L1 |
| 211126_s_at | 2.84E-05 | 2.97E-07 | -5.93579 | 6.625542 | -1.75295 | CSRP2 |
| 206433_s_at | 2.20E-04 | 4.10E-06 | -5.18633 | 4.10659 | -1.74947 | SPOCK3 |
| 220233_at | 1.26E-06 | 5.55E-09 | -7.05446 | 10.4546 | -1.74821 | FBXO17 |
| 222265_at | 6.59E-07 | 2.41E-09 | -7.28892 | 11.25713 | -1.74808 | TNS4 |
| 213931_at | 3.55E-03 | 1.54E-04 | -4.10308 | 0.654609 | -1.74414 | ID2 |
| 202269_x_at | 6.55E-04 | 1.70E-05 | -4.7709 | 2.748251 | -1.74245 | GBP1 |
| 219436_s_at | 4.11E-04 | 9.33E-06 | -4.94724 | 3.320556 | -1.73971 | EMCN |
| 219288_at | 5.02E-02 | 5.85E-03 | -2.88259 | -2.72507 | -1.73965 | C3orf14 |
| 222784_at | 1.66E-04 | 2.91E-06 | -5.28503 | 4.434053 | -1.73964 | SMOC1 |
| 201641_at | 2.17E-04 | 4.03E-06 | -5.19101 | 4.122065 | -1.73905 | BST2 |
| 205083_at | 6.68E-06 | 4.80E-08 | -6.44871 | 8.37761 | -1.73867 | AOX1 |
| 1559296_at | 1.51E-07 | 3.99E-10 | -7.79681 | 12.98709 | -1.73538 | ADAMTS9-AS2 |
| 201753_s_at | 2.23E-03 | 8.45E-05 | -4.2886 | 1.222877 | -1.73452 | ADD3 |
| 208763_s_at | 4.83E-07 | 1.70E-09 | -7.38822 | 11.59639 | -1.73365 | TSC22D3 |
| 202756_s_at | 2.81E-05 | 2.93E-07 | -5.93981 | 6.639225 | -1.73258 | GPC1 |
| 229853_at | 1.46E-04 | 2.47E-06 | -5.3325 | 4.592095 | -1.72699 | KAL1 |
| 226764_at | 1.96E-05 | 1.82E-07 | -6.07372 | 7.095312 | -1.72592 | ZNF827 |
| 227721_at | 1.11E-04 | 1.74E-06 | -5.43251 | 4.926151 | -1.72425 | CPAMD8 |
| 207144_s_at | 2.85E-02 | 2.61E-03 | -3.17245 | -1.98889 | -1.71884 | CITED1 |
| 220416_at | 6.27E-05 | 8.30E-07 | -5.64424 | 5.63778 | -1.71693 | ATP8B4 |
| 204636_at | 6.68E-07 | 2.46E-09 | -7.28381 | 11.23965 | -1.7164 | COL17A1 |
| 200923_at | 3.01E-04 | 6.19E-06 | -5.06686 | 3.712458 | -1.71356 | LGALS3BP |
| 243123_at | 7.12E-05 | 9.77E-07 | -5.59782 | 5.481302 | -1.71235 | RRN3P2 |
| 209466_x_at | 5.04E-03 | 2.45E-04 | -3.95735 | 0.216635 | -1.7118 | PTN |
| 1555691_a_at | 1.64E-02 | 1.22E-03 | -3.43477 | -1.28365 | -1.71067 | KLRC4-KLRK1 |
| 229963_at | 8.78E-02 | 1.38E-02 | -2.55359 | -3.49943 | -1.70965 | BEX5 |
| 207705_s_at | 6.35E-04 | 1.63E-05 | -4.78353 | 2.789031 | -1.70903 | NINL |
| 203510_at | 2.23E-04 | 4.18E-06 | -5.18055 | 4.087453 | -1.70575 | MET |
| 210684_s_at | 4.85E-04 | 1.15E-05 | -4.88653 | 3.122752 | -1.7053 | DLG4 |
| 236785_at | 1.90E-07 | 5.22E-10 | -7.72113 | 12.73025 | -1.70006 | LOC101928461 |
| 200824_at | 1.46E-05 | 1.25E-07 | -6.17926 | 7.455552 | -1.69789 | GSTP1 |
| 232098_at | 2.65E-05 | 2.69E-07 | -5.96336 | 6.719339 | -1.69716 | DST |
| 233317_at | 3.68E-03 | 1.63E-04 | -4.08582 | 0.602351 | -1.69643 | CD9 |
| 209735_at | 1.94E-05 | 1.78E-07 | -6.07951 | 7.115053 | -1.69622 | ABCG2 |
| 207050_at | 1.80E-03 | 6.36E-05 | -4.37521 | 1.491948 | -1.6962 | CACNA2D1 |
| 202283_at | 2.86E-04 | 5.79E-06 | -5.08619 | 3.776072 | -1.69606 | SERPINF1 |
| 1555233_at | 3.67E-03 | 1.62E-04 | -4.08749 | 0.607396 | -1.69593 | RHOJ |
| 224524_s_at | 8.25E-06 | 6.16E-08 | -6.37889 | 8.138419 | -1.69476 | ASB3 |
| 210984_x_at | 1.85E-05 | 1.66E-07 | -6.10011 | 7.185307 | -1.69354 | EGFR |
| 206339_at | 1.44E-02 | 1.02E-03 | -3.49529 | -1.11608 | -1.68746 | CARTPT |
| 225945_at | 2.95E-03 | 1.21E-04 | -4.17731 | 0.880595 | -1.68425 | ZNF655 |
| 226829_at | 1.71E-04 | 3.04E-06 | -5.27313 | 4.394482 | -1.68331 | AFAP1L2 |
| 208964_s_at | 2.14E-05 | 2.05E-07 | -6.04059 | 6.982357 | -1.6833 | FADS1 |
| 1554835_a_at | 1.57E-03 | 5.34E-05 | -4.4281 | 1.657385 | -1.67765 | B3GNT5 |
| 225464_at | 2.04E-05 | 1.93E-07 | -6.05794 | 7.041506 | -1.67721 | FRMD6 |
| 209603_at | 5.66E-03 | 2.86E-04 | -3.90807 | 0.070315 | -1.67605 | GATA3 |
| 217974_at | 5.18E-05 | 6.38E-07 | -5.71927 | 5.891194 | -1.67591 | TM7SF3 |
| 209146_at | 9.49E-05 | 1.43E-06 | -5.49008 | 5.119075 | -1.67554 | MSMO1 |
| 203400_s_at | 2.42E-02 | 2.07E-03 | -3.25316 | -1.77568 | -1.67488 | TF |
| 1568713_a_at | 7.25E-07 | 2.74E-09 | -7.25259 | 11.13288 | -1.67336 | TBC1D1 |
| 227889_at | 2.27E-04 | 4.29E-06 | -5.17324 | 4.063281 | -1.67094 | LPCAT2 |
| 201866_s_at | 2.92E-05 | 3.08E-07 | -5.92558 | 6.590836 | -1.67021 | NR3C1 |
| 1570534_a_at | 1.35E-06 | 6.06E-09 | -7.02986 | 10.37031 | -1.66699 | ZNF483 |
| 206348_s_at | 2.49E-04 | 4.83E-06 | -5.13857 | 3.948726 | -1.66384 | PDK3 |
| 227909_at | 1.32E-03 | 4.26E-05 | -4.49656 | 1.872741 | -1.65987 | LINC00086 |
| 209540_at | 1.01E-02 | 6.29E-04 | -3.65421 | -0.66801 | -1.65953 | IGF1 |
| 231882_at | 7.06E-02 | 9.77E-03 | -2.68932 | -3.18829 | -1.65688 | AP000525.9 |
| 229341_at | 2.21E-05 | 2.13E-07 | -6.02954 | 6.944691 | -1.65565 | TFCP2L1 |
| 208914_at | 1.36E-07 | 3.31E-10 | -7.85026 | 13.16823 | -1.65554 | GGA2 |
| 214063_s_at | 1.77E-02 | 1.34E-03 | -3.402 | -1.37365 | -1.65515 | TF |
| 225265_at | 1.09E-03 | 3.33E-05 | -4.5709 | 2.108051 | -1.65453 | RBMS1 |
| 218589_at | 6.55E-06 | 4.66E-08 | -6.45743 | 8.407479 | -1.65427 | LPAR6 |
| 205728_at | 6.79E-02 | 9.20E-03 | -2.71225 | -3.13455 | -1.6531 | TENM1 |
| 209373_at | 1.50E-03 | 5.02E-05 | -4.44727 | 1.717549 | -1.65244 | MALL |
| 212249_at | 2.00E-07 | 5.53E-10 | -7.70461 | 12.67413 | -1.6475 | PIK3R1 |
| 205236_x_at | 8.60E-04 | 2.44E-05 | -4.66372 | 2.403931 | -1.64669 | SOD3 |
| 1553007_a_at | 5.09E-02 | 5.97E-03 | -2.87484 | -2.74409 | -1.64506 | TENM1 |
| 238066_at | 8.58E-05 | 1.24E-06 | -5.529 | 5.249749 | -1.64225 | RBP7 |
| 32137_at | 1.90E-04 | 3.42E-06 | -5.23853 | 4.279573 | -1.64224 | JAG2 |
| 235044_at | 4.86E-04 | 1.15E-05 | -4.88559 | 3.119716 | -1.64122 | CYYR1 |
| 201169_s_at | 1.41E-02 | 9.92E-04 | -3.50381 | -1.09236 | -1.63918 | BHLHE40 |
| 221884_at | 8.15E-04 | 2.28E-05 | -4.68375 | 2.468071 | -1.63875 | MECOM |
| 227874_at | 3.78E-04 | 8.36E-06 | -4.97915 | 3.424812 | -1.63284 | EMCN |
| 215345_x_at | 8.34E-02 | 1.27E-02 | -2.58598 | -3.42627 | -1.63232 | AK291611 |
| 238673_at | 2.32E-03 | 8.90E-05 | -4.2728 | 1.174036 | -1.6319 | SAMD12 |
| 225288_at | 3.62E-06 | 2.14E-08 | -6.67552 | 9.15523 | -1.6295 | COL27A1 |
| 201170_s_at | 6.06E-02 | 7.70E-03 | -2.77991 | -2.97405 | -1.6292 | BHLHE40 |
| 201012_at | 2.07E-05 | 1.97E-07 | -6.05191 | 7.020948 | -1.62801 | ANXA1 |
| 214340_at | 4.56E-04 | 1.06E-05 | -4.90877 | 3.195129 | -1.62597 | ALOX12P2 |
| 230782_at | 2.43E-03 | 9.37E-05 | -4.2568 | 1.124661 | -1.62551 | SORD |
| 223393_s_at | 4.07E-04 | 9.15E-06 | -4.95277 | 3.338601 | -1.62439 | TSHZ3 |
| 221541_at | 3.93E-04 | 8.76E-06 | -4.9656 | 3.380518 | -1.62429 | CRISPLD2 |
| 239598_s_at | 6.39E-05 | 8.55E-07 | -5.63598 | 5.609889 | -1.62396 | LPCAT2 |
| 212226_s_at | 1.00E-03 | 2.97E-05 | -4.60491 | 2.216188 | -1.62328 | PPAP2B |
| 227461_at | 1.16E-02 | 7.58E-04 | -3.59327 | -0.84119 | -1.62084 | STON2 |
| 235606_at | 5.74E-03 | 2.92E-04 | -3.90206 | 0.052551 | -1.62081 | LINC00883 |
| 230931_at | 3.44E-02 | 3.41E-03 | -3.07871 | -2.23217 | -1.6185 | PLG |
| 220617_s_at | 1.10E-03 | 3.35E-05 | -4.5685 | 2.100433 | -1.61838 | ZNF532 |
| 211607_x_at | 4.37E-05 | 5.08E-07 | -5.78381 | 6.109646 | -1.61676 | EGFR |
| 211737_x_at | 5.82E-03 | 2.98E-04 | -3.89535 | 0.032719 | -1.6149 | PTN |
| 213772_s_at | 1.25E-07 | 2.98E-10 | -7.87978 | 13.26821 | -1.61449 | GGA2 |
| 40837_at | 1.29E-05 | 1.06E-07 | -6.226 | 7.615302 | -1.61015 | TLE2 |
| 228176_at | 4.52E-03 | 2.14E-04 | -4.00056 | 0.345693 | -1.61002 | S1PR3 |
| 228255_at | 4.97E-07 | 1.77E-09 | -7.37543 | 11.55272 | -1.60933 | TMEM237 |
| 1561421_a_at | 1.75E-01 | 4.82E-02 | -2.02659 | -4.58604 | -1.60604 | AK057259 |
| 214676_x_at | 5.82E-02 | 7.24E-03 | -2.80286 | -2.91896 | -1.60528 | MUC3B |
| 224992_s_at | 3.67E-05 | 4.11E-07 | -5.84382 | 6.313129 | -1.60371 | CMIP |
| 201034_at | 2.68E-05 | 2.73E-07 | -5.95936 | 6.705739 | -1.60143 | ADD3 |
| 1558777_at | 5.20E-03 | 2.55E-04 | -3.94458 | 0.178645 | -1.60037 | MKL2 |
| 213006_at | 7.76E-02 | 1.13E-02 | -2.63206 | -3.32101 | -1.59979 | CEBPD |
| 229222_at | 4.75E-03 | 2.28E-04 | -3.98069 | 0.28628 | -1.59957 | ACSS3 |
| 202207_at | 1.60E-03 | 5.50E-05 | -4.4196 | 1.630727 | -1.59881 | ARL4C |
| 202196_s_at | 1.73E-05 | 1.54E-07 | -6.12155 | 7.258505 | -1.59806 | DKK3 |
| 206068_s_at | 1.37E-03 | 4.47E-05 | -4.4822 | 1.827433 | -1.59802 | ACADL |
| 235214_at | 3.82E-08 | 6.38E-11 | -8.31954 | 14.74929 | -1.59718 | LURAP1 |
| 234418_x_at | 1.41E-02 | 9.84E-04 | -3.50663 | -1.08449 | -1.59626 | CD44 |
| 207864_at | 7.42E-04 | 1.99E-05 | -4.7238 | 2.596597 | -1.5962 | SCN7A |
| 212599_at | 1.16E-03 | 3.58E-05 | -4.54858 | 2.037234 | -1.59325 | AUTS2 |
| 201841_s_at | 2.32E-04 | 4.41E-06 | -5.16527 | 4.036919 | -1.5928 | HSPB1 |
| 231672_at | 4.38E-03 | 2.04E-04 | -4.01594 | 0.391792 | -1.59164 | LOC100653086 |
| 205578_at | 1.47E-07 | 3.82E-10 | -7.80957 | 13.03037 | -1.59073 | ROR2 |
| 221957_at | 3.54E-06 | 2.09E-08 | -6.68283 | 9.180306 | -1.59004 | PDK3 |
| 202391_at | 7.27E-03 | 4.04E-04 | -3.79793 | -0.25326 | -1.58981 | BASP1 |
| 230887_at | 1.68E-05 | 1.48E-07 | -6.13276 | 7.296739 | -1.58953 | CDC14B |
| 204990_s_at | 2.59E-05 | 2.59E-07 | -5.9745 | 6.757251 | -1.58713 | ITGB4 |
| 208915_s_at | 7.75E-06 | 5.70E-08 | -6.40065 | 8.212957 | -1.58613 | GGA2 |
| 203065_s_at | 4.09E-03 | 1.87E-04 | -4.04305 | 0.473262 | -1.58547 | CAV1 |
| 228735_s_at | 7.82E-06 | 5.76E-08 | -6.39752 | 8.202232 | -1.58471 | PANK2 |
| 225612_s_at | 6.35E-03 | 3.35E-04 | -3.85815 | -0.07695 | -1.58469 | B3GNT5 |
| 213094_at | 1.74E-03 | 6.12E-05 | -4.38707 | 1.528967 | -1.581 | GPR126 |
| 1557961_s_at | 6.17E-05 | 8.09E-07 | -5.65151 | 5.662281 | -1.57675 | C8orf88 |
| 231711_at | 3.34E-03 | 1.42E-04 | -4.12782 | 0.729732 | -1.57466 | ACPP |
| 201136_at | 1.56E-04 | 2.68E-06 | -5.30899 | 4.513782 | -1.57458 | PLP2 |
| 1567440_at | 1.42E-02 | 9.95E-04 | -3.50274 | -1.09533 | -1.57431 | PSEN1 |
| 228726_at | 1.03E-04 | 1.60E-06 | -5.45772 | 5.010592 | -1.57232 | SERPINB1 |
| 204755_x_at | 2.14E-05 | 2.05E-07 | -6.04066 | 6.982589 | -1.57042 | HLF |
| 226506_at | 3.82E-05 | 4.33E-07 | -5.82908 | 6.263096 | -1.57011 | THSD4 |
| 213827_at | 6.40E-03 | 3.39E-04 | -3.85427 | -0.08834 | -1.56976 | ARHGAP33 |
| 212850_s_at | 3.34E-04 | 7.18E-06 | -5.02347 | 3.570003 | -1.56975 | LRP4 |
| 241614_at | 1.80E-02 | 1.38E-03 | -3.39312 | -1.39794 | -1.56974 | RP11-471B22.2 |
| 207630_s_at | 1.04E-03 | 3.12E-05 | -4.58984 | 2.168228 | -1.56934 | CREM |
| 206434_at | 2.01E-03 | 7.35E-05 | -4.33119 | 1.354886 | -1.56906 | SPOCK3 |
| 205816_at | 2.44E-03 | 9.45E-05 | -4.25421 | 1.116689 | -1.56808 | ITGB8 |
| 221016_s_at | 3.73E-08 | 6.04E-11 | -8.33525 | 14.80189 | -1.56805 | TCF7L1 |
| 243041_s_at | 1.23E-03 | 3.88E-05 | -4.52491 | 1.962279 | -1.56608 | FAM73A |
| 228573_at | 1.43E-03 | 4.74E-05 | -4.46454 | 1.771844 | -1.56508 | ANTXR2 |
| 205158_at | 1.24E-04 | 2.00E-06 | -5.39279 | 4.793313 | -1.56439 | RNASE4 |
| 201667_at | 4.21E-06 | 2.60E-08 | -6.62078 | 8.967498 | -1.56126 | GJA1 |
| 209687_at | 3.66E-03 | 1.61E-04 | -4.08912 | 0.612348 | -1.56105 | CXCL12 |
| 239654_at | 6.31E-03 | 3.32E-04 | -3.86129 | -0.06772 | -1.56025 | CHD9 |
| 242321_at | 2.35E-04 | 4.49E-06 | -5.16009 | 4.019813 | -1.56021 | PTPN14 |
| 225524_at | 8.09E-05 | 1.15E-06 | -5.55093 | 5.323488 | -1.55757 | ANTXR2 |
| 222274_at | 3.65E-02 | 3.69E-03 | -3.05004 | -2.30559 | -1.55721 | ZDHHC8P1 |
| 204038_s_at | 1.09E-04 | 1.70E-06 | -5.43959 | 4.949856 | -1.55628 | LPAR1 |
| 213469_at | 1.39E-04 | 2.31E-06 | -5.35202 | 4.657161 | -1.5552 | PGAP1 |
| 229791_at | 2.21E-04 | 4.14E-06 | -5.18362 | 4.097637 | -1.55463 | LPCAT2 |
| 242912_at | 1.05E-01 | 1.88E-02 | -2.43069 | -3.77055 | -1.55346 | POTEM |
| 238478_at | 2.84E-03 | 1.15E-04 | -4.19387 | 0.931278 | -1.5518 | BNC2 |
| 205413_at | 5.23E-03 | 2.58E-04 | -3.94126 | 0.168772 | -1.55056 | MPPED2 |
| 223434_at | 1.59E-02 | 1.17E-03 | -3.44978 | -1.24226 | -1.54917 | GBP3 |
| 241762_at | 1.97E-02 | 1.55E-03 | -3.35267 | -1.50813 | -1.54668 | FBXO32 |
| 221854_at | 7.81E-02 | 1.14E-02 | -2.62806 | -3.33021 | -1.54566 | PKP1 |
| 228504_at | 2.01E-03 | 7.38E-05 | -4.33003 | 1.351299 | -1.54558 | SCN7A |
| 227099_s_at | 3.19E-02 | 3.05E-03 | -3.11825 | -2.13013 | -1.54536 | C11orf96 |
| 221110_x_at | 1.44E-04 | 2.42E-06 | -5.338 | 4.610414 | -1.54432 | PDE11A |
| 232034_at | 7.21E-03 | 4.00E-04 | -3.80162 | -0.24249 | -1.54411 | LINC00537 |
| 228310_at | 3.68E-03 | 1.63E-04 | -4.08625 | 0.603641 | -1.54238 | ENAH |
| 213348_at | 1.68E-05 | 1.47E-07 | -6.13509 | 7.304709 | -1.54122 | CDKN1C |
| 218824_at | 5.71E-03 | 2.90E-04 | -3.90413 | 0.058674 | -1.53878 | PNMAL1 |
| 212724_at | 9.95E-04 | 2.95E-05 | -4.60714 | 2.223305 | -1.53662 | RND3 |
| 241596_at | 1.01E-07 | 2.24E-10 | -7.96078 | 13.54216 | -1.53528 | NUDT10 |
| 225021_at | 2.95E-03 | 1.22E-04 | -4.17637 | 0.877743 | -1.52814 | ZNF532 |
| 230464_at | 4.63E-03 | 2.20E-04 | -3.99117 | 0.317595 | -1.52795 | S1PR5 |
| 235457_at | 4.12E-05 | 4.74E-07 | -5.80356 | 6.176569 | -1.52741 | MAML2 |
| 205043_at | 6.05E-02 | 7.67E-03 | -2.78125 | -2.97085 | -1.52678 | CFTR |
| 222458_s_at | 3.41E-04 | 7.35E-06 | -5.01681 | 3.548145 | -1.52662 | AKIRIN1 |
| 213397_x_at | 1.33E-05 | 1.10E-07 | -6.21471 | 7.57671 | -1.52508 | RNASE4 |
| 214031_s_at | 4.29E-02 | 4.66E-03 | -2.96588 | -2.51847 | -1.5208 | KRT7 |
| 216291_at | 9.65E-04 | 2.83E-05 | -4.61957 | 2.262908 | -1.51942 | ZNF440 |
| 209170_s_at | 8.27E-05 | 1.19E-06 | -5.54185 | 5.292936 | -1.51804 | GPM6B |
| 237939_at | 3.30E-03 | 1.40E-04 | -4.13234 | 0.743475 | -1.5178 | EPHA5 |
| 208949_s_at | 2.32E-04 | 4.41E-06 | -5.16504 | 4.036184 | -1.51765 | LGALS3 |
| 1554147_s_at | 1.53E-03 | 5.14E-05 | -4.43982 | 1.694164 | -1.51612 | MAATS1 |
| 211488_s_at | 1.64E-03 | 5.66E-05 | -4.41075 | 1.603015 | -1.51509 | ITGB8 |
| 215411_s_at | 2.83E-06 | 1.57E-08 | -6.76295 | 9.455114 | -1.51492 | TRAF3IP2 |
| 228988_at | 1.33E-05 | 1.11E-07 | -6.21426 | 7.575167 | -1.51191 | ZNF711 |
| 215311_at | 2.77E-05 | 2.85E-07 | -5.9471 | 6.664019 | -1.51174 | NTRK3 |
| 221556_at | 5.73E-05 | 7.31E-07 | -5.68046 | 5.760027 | -1.50928 | CDC14B |
| 236950_s_at | 1.48E-02 | 1.06E-03 | -3.48217 | -1.15256 | -1.5063 | LINC00964 |
| 220022_at | 7.00E-03 | 3.84E-04 | -3.81478 | -0.20409 | -1.50615 | ZNF334 |
| 204011_at | 1.09E-05 | 8.72E-08 | -6.28104 | 7.803527 | -1.5036 | SPRY2 |
| 207254_at | 5.13E-04 | 1.24E-05 | -4.86355 | 3.048096 | -1.50144 | SLC15A1 |
| 231887_s_at | 3.69E-04 | 8.13E-06 | -4.98727 | 3.4514 | -1.50144 | PALD1 |
| 201287_s_at | 3.42E-02 | 3.37E-03 | -3.08226 | -2.22303 | -1.49952 | SDC1 |
| 227317_at | 4.92E-04 | 1.17E-05 | -4.88014 | 3.10199 | -1.49947 | LMCD1 |
| 219944_at | 2.10E-05 | 1.99E-07 | -6.04822 | 7.008348 | -1.49895 | CLIP4 |
| 214278_s_at | 1.43E-06 | 6.58E-09 | -7.00679 | 10.29124 | -1.49813 | NDRG2 |
| 231704_at | 1.44E-06 | 6.65E-09 | -7.00398 | 10.2816 | -1.49692 | CYP3A4 |
| 229176_at | 4.94E-03 | 2.39E-04 | -3.96608 | 0.24265 | -1.49282 | ANKH |
| 203811_s_at | 6.12E-04 | 1.55E-05 | -4.79784 | 2.835247 | -1.48915 | DNAJB4 |
| 201625_s_at | 2.94E-03 | 1.21E-04 | -4.17849 | 0.88421 | -1.48906 | INSIG1 |
| 203666_at | 5.59E-03 | 2.81E-04 | -3.91382 | 0.087343 | -1.48901 | CXCL12 |
| 204393_s_at | 2.51E-03 | 9.76E-05 | -4.2445 | 1.08676 | -1.48898 | ACPP |
| 233551_at | 1.09E-03 | 3.32E-05 | -4.57193 | 2.111309 | -1.48792 | LOC642776 |
| 208512_s_at | 1.65E-04 | 2.90E-06 | -5.28665 | 4.439443 | -1.48781 | MLLT4 |
| 1555754_s_at | 1.76E-06 | 8.53E-09 | -6.93383 | 10.04115 | -1.48667 | ATN1 |
| 206030_at | 1.81E-03 | 6.44E-05 | -4.37134 | 1.479878 | -1.48632 | ASPA |
| 222008_at | 7.74E-03 | 4.38E-04 | -3.77195 | -0.32887 | -1.48559 | COL9A1 |
| 207001_x_at | 6.50E-06 | 4.60E-08 | -6.46098 | 8.419657 | -1.48496 | TSC22D3 |
| 230785_at | 7.78E-02 | 1.14E-02 | -2.63042 | -3.32479 | -1.48405 | SALL3 |
| 214995_s_at | 1.13E-03 | 3.46E-05 | -4.55933 | 2.071311 | -1.48396 | APOBEC3F |
| 1561817_at | 2.20E-02 | 1.82E-03 | -3.29757 | -1.6569 | -1.48029 | AX747630 |
| 222599_s_at | 2.23E-07 | 6.42E-10 | -7.66217 | 12.52992 | -1.48019 | NAV2 |
| 222486_s_at | 2.43E-02 | 2.09E-03 | -3.25039 | -1.78306 | -1.4797 | ADAMTS1 |
| 231785_at | 8.39E-10 | 6.45E-13 | -9.66148 | 19.14825 | -1.47931 | CGB7 |
| 206506_s_at | 7.89E-06 | 5.86E-08 | -6.39276 | 8.185923 | -1.47737 | SUPT3H |
| 226827_at | 1.36E-05 | 1.15E-07 | -6.20437 | 7.541358 | -1.47511 | TMEM165 |
| 214051_at | 1.36E-02 | 9.44E-04 | -3.52055 | -1.04564 | -1.4741 | TMSB15A |
| 204589_at | 1.79E-04 | 3.21E-06 | -5.25743 | 4.342309 | -1.4711 | NUAK1 |
| 209542_x_at | 6.44E-03 | 3.42E-04 | -3.85133 | -0.09698 | -1.46693 | IGF1 |
| 201631_s_at | 1.59E-03 | 5.44E-05 | -4.42266 | 1.640341 | -1.46674 | IER3 |
| 204136_at | 4.78E-06 | 3.07E-08 | -6.57411 | 8.80743 | -1.46664 | COL7A1 |
| 210562_at | 1.69E-02 | 1.26E-03 | -3.42299 | -1.31604 | -1.46651 | GREB1 |
| 1553626_a_at | 9.36E-03 | 5.68E-04 | -3.6876 | -0.57244 | -1.46297 | EFCAB13 |
| 1555383_a_at | 2.80E-02 | 2.55E-03 | -3.18125 | -1.96582 | -1.46244 | POF1B |
| 207836_s_at | 7.35E-05 | 1.02E-06 | -5.58693 | 5.444608 | -1.4616 | RBPMS |
| 219511_s_at | 7.96E-04 | 2.21E-05 | -4.69343 | 2.499105 | -1.46129 | SNCAIP |
| 214767_s_at | 1.97E-03 | 7.18E-05 | -4.33848 | 1.377556 | -1.46039 | HSPB6 |
| 205304_s_at | 1.23E-04 | 1.96E-06 | -5.39867 | 4.812949 | -1.45915 | KCNJ8 |
| 201842_s_at | 2.23E-03 | 8.44E-05 | -4.28882 | 1.223544 | -1.45741 | EFEMP1 |
| 222725_s_at | 2.61E-04 | 5.15E-06 | -5.12033 | 3.888545 | -1.45737 | PALMD |
| 225016_at | 5.06E-04 | 1.22E-05 | -4.86922 | 3.066511 | -1.45672 | APCDD1 |
| 63305_at | 1.75E-02 | 1.32E-03 | -3.40696 | -1.36005 | -1.45383 | PKNOX2 |
| 219450_at | 1.40E-02 | 9.74E-04 | -3.50981 | -1.07561 | -1.45198 | C4orf19 |
| 227971_at | 5.08E-02 | 5.96E-03 | -2.87568 | -2.74202 | -1.45159 | NRK |
| 226926_at | 1.36E-08 | 1.82E-11 | -8.68072 | 15.95268 | -1.45131 | DMKN |
| 210544_s_at | 6.71E-05 | 9.06E-07 | -5.61935 | 5.553836 | -1.45063 | ALDH3A2 |
| 212510_at | 9.59E-04 | 2.80E-05 | -4.6225 | 2.27226 | -1.45025 | GPD1L |
| 1560446_at | 8.04E-04 | 2.24E-05 | -4.68932 | 2.48591 | -1.44944 | IPO5P1 |
| 207030_s_at | 8.42E-04 | 2.37E-05 | -4.67272 | 2.432723 | -1.44811 | CSRP2 |
| 206600_s_at | 9.16E-08 | 1.98E-10 | -7.99647 | 13.66273 | -1.44688 | SLC16A5 |
| 225627_s_at | 7.42E-04 | 2.00E-05 | -4.72301 | 2.594074 | -1.44632 | CACHD1 |
| 228107_at | 2.76E-06 | 1.50E-08 | -6.77579 | 9.49915 | -1.44499 | C8orf88 |
| 236023_at | 6.42E-04 | 1.65E-05 | -4.77905 | 2.774553 | -1.44457 | CDK9 |
| 227812_at | 1.60E-01 | 4.07E-02 | -2.10195 | -4.44318 | -1.444 | TNFRSF19 |
| 228407_at | 6.71E-04 | 1.75E-05 | -4.76159 | 2.718251 | -1.44291 | SCUBE3 |
| 204310_s_at | 2.36E-05 | 2.31E-07 | -6.00642 | 6.865924 | -1.44215 | NPR2 |
| 217014_s_at | 2.82E-02 | 2.57E-03 | -3.17855 | -1.9729 | -1.44128 | AZGP1 |
| 213710_s_at | 1.68E-05 | 1.47E-07 | -6.13367 | 7.299845 | -1.44067 | CALM1 |
| 230775_s_at | 7.79E-04 | 2.14E-05 | -4.70242 | 2.527948 | -1.43873 | SPG20 |
| 202206_at | 1.74E-04 | 3.10E-06 | -5.26739 | 4.375401 | -1.43864 | ARL4C |
| 208690_s_at | 2.48E-06 | 1.30E-08 | -6.81475 | 9.632774 | -1.43804 | PDLIM1 |
| 44783_s_at | 3.89E-03 | 1.75E-04 | -4.06418 | 0.536948 | -1.438 | HEY1 |
| 206133_at | 1.95E-03 | 7.06E-05 | -4.3435 | 1.393177 | -1.43634 | XAF1 |
| 210751_s_at | 7.36E-03 | 4.10E-04 | -3.79311 | -0.2673 | -1.43543 | RGN |
| 216085_at | 7.42E-04 | 2.00E-05 | -4.72305 | 2.594188 | -1.43205 | DKFZP434C153 |
| 209168_at | 2.64E-04 | 5.24E-06 | -5.115 | 3.870973 | -1.43022 | GPM6B |
| 1560001_at | 8.79E-05 | 1.30E-06 | -5.51746 | 5.210985 | -1.42717 | LOC100131581 |
| 231291_at | 1.31E-03 | 4.22E-05 | -4.4993 | 1.881368 | -1.42569 | GIPR |
| 240282_at | 1.08E-02 | 6.85E-04 | -3.62645 | -0.74711 | -1.42525 | WDR1 |
| 214925_s_at | 3.64E-03 | 1.60E-04 | -4.09115 | 0.618478 | -1.42398 | SPTAN1 |
| 1553322_s_at | 7.84E-06 | 5.79E-08 | -6.39603 | 8.197144 | -1.4236 | TEAD1 |
| 226625_at | 5.72E-06 | 3.88E-08 | -6.50876 | 8.58339 | -1.42311 | TGFBR3 |
| 1554513_s_at | 1.13E-03 | 3.46E-05 | -4.55938 | 2.071478 | -1.42226 | CEP89 |
| 228456_s_at | 7.46E-04 | 2.01E-05 | -4.72079 | 2.586919 | -1.41989 | CDS2 |
| 201272_at | 8.15E-04 | 2.28E-05 | -4.68409 | 2.469157 | -1.41902 | AKR1B1 |
| 215664_s_at | 4.42E-03 | 2.06E-04 | -4.01181 | 0.379417 | -1.4178 | EPHA5 |
| 207761_s_at | 1.15E-04 | 1.81E-06 | -5.42127 | 4.888523 | -1.4175 | METTL7A |
| 1555347_at | 1.28E-03 | 4.07E-05 | -4.51022 | 1.915848 | -1.41749 | PDXDC1 |
| 211302_s_at | 1.82E-02 | 1.40E-03 | -3.3875 | -1.4133 | -1.41477 | PDE4B |
| 1559400_s_at | 1.25E-02 | 8.44E-04 | -3.55771 | -0.94146 | -1.41428 | PAPPA |
| 208103_s_at | 1.86E-03 | 6.67E-05 | -4.36083 | 1.44711 | -1.41372 | ANP32E |
| 207565_s_at | 4.40E-05 | 5.13E-07 | -5.78105 | 6.100294 | -1.41354 | MR1 |
| 205759_s_at | 1.48E-03 | 4.94E-05 | -4.45165 | 1.731299 | -1.41331 | SULT2B1 |
| 204187_at | 7.49E-03 | 4.20E-04 | -3.78551 | -0.28946 | -1.41244 | GMPR |
| 202861_at | 4.19E-03 | 1.92E-04 | -4.03364 | 0.444977 | -1.41121 | FAH |
| 203780_at | 7.89E-04 | 2.18E-05 | -4.69711 | 2.510896 | -1.40906 | MPZL2 |
| 201599_at | 1.08E-03 | 3.26E-05 | -4.57712 | 2.127805 | -1.40891 | OAT |
| 205803_s_at | 4.71E-05 | 5.59E-07 | -5.7565 | 6.017141 | -1.40804 | TRPC1 |
| 227163_at | 3.04E-02 | 2.85E-03 | -3.14235 | -2.06755 | -1.40507 | GSTO2 |
| 225807_at | 8.68E-07 | 3.51E-09 | -7.18333 | 10.89592 | -1.40506 | AJUBA |
| 235753_at | 3.44E-03 | 1.48E-04 | -4.11533 | 0.691774 | -1.4046 | HOXA7 |
| 230631_s_at | 1.92E-02 | 1.50E-03 | -3.3633 | -1.47924 | -1.40391 | IL10RB-AS1 |
| 1294_at | 1.26E-04 | 2.04E-06 | -5.38758 | 4.775893 | -1.40314 | MIR5193 |
| 1559587_at | 3.99E-04 | 8.94E-06 | -4.95969 | 3.361212 | -1.40278 | SYMPK |
| 243618_s_at | 2.56E-04 | 5.00E-06 | -5.12881 | 3.916522 | -1.40161 | FGFR1OP2 |
| 1569796_s_at | 1.29E-02 | 8.75E-04 | -3.54566 | -0.9753 | -1.40028 | ATRNL1 |
| 213684_s_at | 1.53E-02 | 1.10E-03 | -3.46867 | -1.19 | -1.40009 | PDLIM5 |
| 203934_at | 2.91E-03 | 1.19E-04 | -4.18265 | 0.896941 | -1.39979 | KDR |
| 235489_at | 1.32E-03 | 4.24E-05 | -4.49809 | 1.877564 | -1.39919 | RHOJ |
| 204596_s_at | 2.14E-02 | 1.74E-03 | -3.31272 | -1.61614 | -1.39914 | STC1 |
| 214189_s_at | 5.33E-06 | 3.54E-08 | -6.53474 | 8.672447 | -1.3981 | GGA2 |
| 228445_at | 6.53E-05 | 8.78E-07 | -5.62834 | 5.584132 | -1.39689 | AIFM2 |
| 1567443_x_at | 2.37E-02 | 2.02E-03 | -3.26187 | -1.75245 | -1.39511 | PSEN1 |
| 218675_at | 1.14E-05 | 9.23E-08 | -6.26512 | 7.749068 | -1.39278 | SLC22A17 |
| 229782_at | 4.10E-02 | 4.37E-03 | -2.98952 | -2.45908 | -1.39275 | RMST |
| 216979_at | 9.96E-03 | 6.17E-04 | -3.66098 | -0.64868 | -1.39226 | NR4A3 |
| 223495_at | 5.58E-05 | 7.05E-07 | -5.69063 | 5.794402 | -1.39188 | CCDC8 |
| 204099_at | 1.71E-04 | 3.02E-06 | -5.27437 | 4.398589 | -1.39174 | SMARCD3 |
| 243399_at | 2.59E-05 | 2.59E-07 | -5.97436 | 6.756759 | -1.389 | LTBR |
| 206355_at | 3.07E-05 | 3.27E-07 | -5.90823 | 6.531868 | -1.38895 | GNAL |
| 204326_x_at | 5.33E-02 | 6.37E-03 | -2.85082 | -2.80279 | -1.38876 | MT1X |
| 224901_at | 2.94E-03 | 1.21E-04 | -4.17833 | 0.883729 | -1.38852 | SCD5 |
| 228491_at | 2.73E-02 | 2.46E-03 | -3.19377 | -1.93291 | -1.38848 | KRT19 |
| 232306_at | 3.13E-02 | 2.97E-03 | -3.12733 | -2.1066 | -1.38845 | CDH26 |
| 203217_s_at | 1.65E-03 | 5.69E-05 | -4.40895 | 1.597378 | -1.38751 | ST3GAL5 |
| 203726_s_at | 3.24E-02 | 3.13E-03 | -3.10891 | -2.15433 | -1.38742 | LAMA3 |
| 1552658_a_at | 1.27E-02 | 8.61E-04 | -3.55115 | -0.9599 | -1.38661 | NAV3 |
| 1569044_at | 3.27E-03 | 1.39E-04 | -4.13611 | 0.754957 | -1.38559 | CDC42BPG |
| 235342_at | 1.06E-02 | 6.68E-04 | -3.63486 | -0.72318 | -1.38403 | SPOCK3 |
| 220576_at | 8.09E-05 | 1.15E-06 | -5.55028 | 5.321284 | -1.3834 | PGAP1 |
| 221489_s_at | 8.77E-02 | 1.38E-02 | -2.55455 | -3.49726 | -1.38253 | SPRY4 |
| 226086_at | 7.86E-02 | 1.16E-02 | -2.62365 | -3.34033 | -1.38229 | SYT13 |
| 204029_at | 1.16E-03 | 3.59E-05 | -4.54828 | 2.036284 | -1.38146 | CELSR2 |
| 203324_s_at | 6.19E-05 | 8.13E-07 | -5.65004 | 5.657327 | -1.38133 | CAV2 |
| 204731_at | 1.48E-04 | 2.52E-06 | -5.32666 | 4.572624 | -1.38105 | TGFBR3 |
| 232662_x_at | 9.87E-04 | 2.92E-05 | -4.61 | 2.232396 | -1.38064 | FAM213A |
| 220420_at | 6.84E-05 | 9.29E-07 | -5.61217 | 5.529632 | -1.38058 | LMAN1L |
| 233544_at | 6.49E-04 | 1.68E-05 | -4.77498 | 2.761444 | -1.3796 | GNL1 |
| 212240_s_at | 1.36E-07 | 3.34E-10 | -7.84741 | 13.15857 | -1.37948 | PIK3R1 |
| 222033_s_at | 6.84E-03 | 3.72E-04 | -3.82471 | -0.17502 | -1.37774 | FLT1 |
| 224221_s_at | 6.63E-03 | 3.55E-04 | -3.83967 | -0.13121 | -1.37668 | VAV3 |
| 244508_at | 7.64E-04 | 2.09E-05 | -4.71009 | 2.552555 | -1.37495 | 42985 |
| 215513_at | 5.77E-03 | 2.94E-04 | -3.8995 | 0.044971 | -1.37444 | HYMAI |
| 242809_at | 1.85E-02 | 1.43E-03 | -3.381 | -1.43102 | -1.37296 | IL1RL1 |
| 220119_at | 1.64E-02 | 1.21E-03 | -3.4361 | -1.27999 | -1.37289 | EPB41L4A |
| 223797_at | 1.18E-02 | 7.80E-04 | -3.58372 | -0.86817 | -1.37208 | PRO2852 |
| 240106_at | 4.63E-03 | 2.20E-04 | -3.99157 | 0.318798 | -1.3718 | GNPTAB |
| 224710_at | 3.70E-04 | 8.18E-06 | -4.98565 | 3.44608 | -1.3704 | NARR |
| 230865_at | 9.33E-04 | 2.71E-05 | -4.63255 | 2.304328 | -1.37012 | LIX1 |
| 1553088_a_at | 3.21E-03 | 1.35E-04 | -4.14373 | 0.778151 | -1.36965 | BCL2L11 |
| 211577_s_at | 9.65E-03 | 5.93E-04 | -3.67387 | -0.61179 | -1.36874 | IGF1 |
| 226825_s_at | 1.75E-02 | 1.33E-03 | -3.40642 | -1.36154 | -1.36765 | TMEM165 |
| 206360_s_at | 1.29E-01 | 2.72E-02 | -2.27647 | -4.09588 | -1.36676 | SOCS3 |
| 233663_s_at | 6.43E-02 | 8.43E-03 | -2.7458 | -3.05533 | -1.36475 | CDH26 |
| 209209_s_at | 1.08E-02 | 6.91E-04 | -3.62376 | -0.75474 | -1.36429 | FERMT2 |
| 206011_at | 3.12E-03 | 1.30E-04 | -4.15489 | 0.812148 | -1.36126 | CASP1 |
| 214624_at | 8.60E-05 | 1.25E-06 | -5.52792 | 5.246119 | -1.36088 | UPK1A |
| 218574_s_at | 2.67E-03 | 1.06E-04 | -4.22015 | 1.01189 | -1.36082 | LMCD1 |
| 223313_s_at | 5.62E-02 | 6.86E-03 | -2.82324 | -2.86977 | -1.36081 | MAGED4 |
| 211139_s_at | 1.03E-03 | 3.09E-05 | -4.59323 | 2.179009 | -1.3577 | NAB1 |
| 204574_s_at | 6.77E-03 | 3.66E-04 | -3.8298 | -0.16013 | -1.35726 | MMP19 |
| 1552477_a_at | 3.59E-04 | 7.90E-06 | -4.99588 | 3.479583 | -1.35641 | IRF6 |
| 1554986_a_at | 3.29E-06 | 1.90E-08 | -6.70922 | 9.270818 | -1.35504 | SNX19 |
| 1562234_a_at | 1.65E-02 | 1.22E-03 | -3.43434 | -1.28482 | -1.35409 | NAV3 |
| 218736_s_at | 1.09E-05 | 8.68E-08 | -6.2826 | 7.808856 | -1.35386 | PALMD |
| 200904_at | 1.46E-03 | 4.85E-05 | -4.45722 | 1.748817 | -1.3528 | HLA-E |
| 231783_at | 4.65E-02 | 5.25E-03 | -2.92206 | -2.62768 | -1.35263 | CHRM1 |
| 230085_at | 5.01E-05 | 6.08E-07 | -5.73262 | 5.936328 | -1.35184 | PDK3 |
| 227998_at | 4.29E-08 | 7.45E-11 | -8.27502 | 14.60008 | -1.35155 | S100A16 |
| 1561418_at | 1.48E-05 | 1.27E-07 | -6.17575 | 7.443575 | -1.35066 | LOC101928245 |
| 231577_s_at | 1.22E-02 | 8.14E-04 | -3.56948 | -0.90835 | -1.34895 | GBP1 |
| 1553411_s_at | 6.93E-02 | 9.47E-03 | -2.70134 | -3.16017 | -1.34836 | SALL3 |
| 226863_at | 2.21E-02 | 1.84E-03 | -3.29483 | -1.66426 | -1.3476 | FAM110C |
| 226492_at | 4.99E-04 | 1.19E-05 | -4.87503 | 3.085372 | -1.34711 | SEMA6D |
| 214190_x_at | 7.52E-05 | 1.04E-06 | -5.57952 | 5.419662 | -1.34592 | GGA2 |
| 1559807_at | 8.10E-03 | 4.68E-04 | -3.75051 | -0.39108 | -1.34567 | BC045789 |
| 212558_at | 1.55E-03 | 5.24E-05 | -4.43413 | 1.676307 | -1.34562 | SPRY1 |
| 241601_at | 4.47E-03 | 2.10E-04 | -4.00582 | 0.361471 | -1.34508 | WIPF3 |
| 216361_s_at | 1.55E-06 | 7.28E-09 | -6.97852 | 10.19436 | -1.34298 | KAT6A |
| 206363_at | 2.95E-02 | 2.73E-03 | -3.15655 | -2.03051 | -1.3429 | MAF |
| 244533_at | 1.38E-05 | 1.16E-07 | -6.20076 | 7.52903 | -1.34272 | PTPN14 |
| 228127_at | 2.56E-03 | 9.98E-05 | -4.23752 | 1.065272 | -1.34046 | KCNK3 |
| 224929_at | 1.18E-02 | 7.81E-04 | -3.58344 | -0.86896 | -1.34027 | TMEM173 |
| 227188_at | 7.89E-07 | 3.09E-09 | -7.21949 | 11.01967 | -1.3398 | EVA1C |
| 231259_s_at | 5.96E-03 | 3.08E-04 | -3.88507 | 0.002343 | -1.3389 | CCND2 |
| 201243_s_at | 1.65E-03 | 5.72E-05 | -4.40769 | 1.593446 | -1.33825 | ATP1B1 |
| 222208_s_at | 5.40E-04 | 1.32E-05 | -4.84581 | 2.990525 | -1.33809 | POLR2J4 |
| 204542_at | 4.56E-04 | 1.06E-05 | -4.90883 | 3.195318 | -1.33583 | ST6GALNAC2 |
| 201983_s_at | 4.73E-05 | 5.65E-07 | -5.75379 | 6.007982 | -1.33469 | EGFR |
| 1554003_at | 1.26E-03 | 4.01E-05 | -4.51494 | 1.930781 | -1.33451 | ARHGEF28 |
| 201061_s_at | 1.75E-05 | 1.56E-07 | -6.11752 | 7.244743 | -1.33444 | STOM |
| 203684_s_at | 6.32E-04 | 1.62E-05 | -4.78542 | 2.795111 | -1.33389 | BCL2 |
| 234387_at | 7.85E-04 | 2.16E-05 | -4.69981 | 2.519574 | -1.33306 | COL4A5 |
| 202878_s_at | 3.11E-02 | 2.95E-03 | -3.13005 | -2.09954 | -1.33281 | CYTH1 |
| 210479_s_at | 5.31E-03 | 2.63E-04 | -3.93551 | 0.151675 | -1.33003 | RORA |
| 205128_x_at | 2.57E-03 | 1.01E-04 | -4.23459 | 1.056266 | -1.32816 | PTGS1 |
| 226368_at | 9.54E-05 | 1.43E-06 | -5.48828 | 5.113053 | -1.32804 | CHST11 |
| 224215_s_at | 1.83E-04 | 3.30E-06 | -5.24922 | 4.315068 | -1.32793 | DLL1 |
| 1562761_at | 7.67E-05 | 1.07E-06 | -5.57144 | 5.392477 | -1.32708 | NMRK1 |
| 203921_at | 1.35E-03 | 4.38E-05 | -4.48801 | 1.84576 | -1.3259 | CHST2 |
| 244261_at | 2.60E-03 | 1.02E-04 | -4.23063 | 1.044096 | -1.32545 | IFNLR1 |
| 244498_x_at | 4.06E-05 | 4.64E-07 | -5.80924 | 6.195817 | -1.32518 | UBE2Q2L |
| 207518_at | 3.85E-03 | 1.72E-04 | -4.06857 | 0.550224 | -1.32511 | DGKE |
| 244677_at | 2.96E-02 | 2.75E-03 | -3.15485 | -2.03494 | -1.32482 | MIR6883 |
| 204753_s_at | 3.52E-03 | 1.52E-04 | -4.10632 | 0.664434 | -1.32421 | HLF |
| 205242_at | 7.17E-02 | 1.00E-02 | -2.67896 | -3.21247 | -1.32368 | CXCL13 |
| 1558392_at | 2.04E-04 | 3.74E-06 | -5.21263 | 4.193703 | -1.32336 | SYNE2 |
| 222719_s_at | 1.60E-03 | 5.51E-05 | -4.41889 | 1.628514 | -1.3228 | PDGFC |
| 219834_at | 9.10E-06 | 6.89E-08 | -6.34738 | 8.030537 | -1.32249 | CARF |
| 208926_at | 8.57E-02 | 1.33E-02 | -2.56872 | -3.46535 | -1.32025 | NEU1 |
| 216236_s_at | 2.16E-02 | 1.77E-03 | -3.30811 | -1.62855 | -1.31818 | SLC2A14 |
| 213993_at | 1.37E-02 | 9.50E-04 | -3.5182 | -1.05219 | -1.31748 | SPON1 |
| 204745_x_at | 1.45E-01 | 3.37E-02 | -2.18513 | -4.28046 | -1.31637 | MT1G |
| 206701_x_at | 3.90E-02 | 4.06E-03 | -3.01536 | -2.3938 | -1.31525 | EDNRB |
| 221009_s_at | 8.56E-04 | 2.42E-05 | -4.66584 | 2.410702 | -1.31525 | ANGPTL4 |
| 215813_s_at | 1.12E-02 | 7.27E-04 | -3.60672 | -0.80311 | -1.31162 | PTGS1 |
| 218017_s_at | 4.23E-08 | 7.28E-11 | -8.28175 | 14.62266 | -1.31079 | HGSNAT |
| 205317_s_at | 6.34E-02 | 8.26E-03 | -2.75315 | -3.03788 | -1.31051 | SLC15A2 |
| 237860_at | 1.12E-03 | 3.45E-05 | -4.5602 | 2.074084 | -1.31038 | RBMS3 |
| 209792_s_at | 3.37E-03 | 1.44E-04 | -4.12449 | 0.719621 | -1.30958 | KLK10 |
| 215346_at | 1.24E-04 | 1.99E-06 | -5.3949 | 4.800359 | -1.30903 | CD40 |
| 1558739_at | 9.39E-03 | 5.71E-04 | -3.6859 | -0.57733 | -1.30861 | LOH12CR1 |
| 235898_at | 9.93E-07 | 4.18E-09 | -7.13453 | 10.72885 | -1.30844 | LOC102723845 |
| 241938_at | 3.86E-03 | 1.73E-04 | -4.06679 | 0.544846 | -1.30694 | QKI |
| 218723_s_at | 5.02E-02 | 5.85E-03 | -2.88223 | -2.72596 | -1.30668 | RGCC |
| 211473_s_at | 6.63E-08 | 1.33E-10 | -8.10867 | 14.04109 | -1.30631 | COL4A6 |
| 209309_at | 2.77E-02 | 2.51E-03 | -3.18638 | -1.95236 | -1.3056 | AZGP1 |
| 231083_at | 3.12E-02 | 2.96E-03 | -3.12812 | -2.10454 | -1.3052 | ETV5 |
| 222747_s_at | 1.31E-04 | 2.14E-06 | -5.37371 | 4.729574 | -1.30465 | SCML1 |
| 205431_s_at | 1.03E-01 | 1.80E-02 | -2.44885 | -3.73114 | -1.30445 | BMP5 |
| 202995_s_at | 3.34E-03 | 1.42E-04 | -4.12821 | 0.730921 | -1.30434 | FBLN1 |
| 201403_s_at | 7.97E-04 | 2.21E-05 | -4.69272 | 2.496804 | -1.30386 | MGST3 |
| 216248_s_at | 1.40E-01 | 3.16E-02 | -2.21361 | -4.22357 | -1.30251 | NR4A2 |
| 213061_s_at | 2.83E-03 | 1.15E-04 | -4.19518 | 0.935286 | -1.3006 | NTAN1 |
| 205316_at | 5.84E-02 | 7.28E-03 | -2.80099 | -2.92347 | -1.3003 | SLC15A2 |
| 203281_s_at | 3.06E-04 | 6.32E-06 | -5.06056 | 3.691762 | -1.30016 | MIR5193 |
| 216929_x_at | 1.39E-04 | 2.31E-06 | -5.35141 | 4.655151 | -1.29807 | ABO |
| 232683_s_at | 3.21E-03 | 1.35E-04 | -4.14419 | 0.779539 | -1.29807 | PARP6 |
| 212268_at | 1.25E-03 | 3.94E-05 | -4.52014 | 1.947193 | -1.29793 | SERPINB1 |
| 219654_at | 1.81E-05 | 1.62E-07 | -6.10668 | 7.20775 | -1.29529 | PTPLA |
| 201645_at | 5.34E-03 | 2.66E-04 | -3.93179 | 0.140628 | -1.29379 | TNC |
| 226534_at | 2.94E-02 | 2.73E-03 | -3.15739 | -2.02832 | -1.29329 | KITLG |
| 238716_at | 2.87E-03 | 1.17E-04 | -4.18778 | 0.912624 | -1.29317 | LOC100506990 |
| 224673_at | 2.06E-04 | 3.80E-06 | -5.20862 | 4.180398 | -1.29232 | LENG8 |
| 211663_x_at | 1.16E-02 | 7.65E-04 | -3.58994 | -0.85059 | -1.2914 | PTGDS |
| 235024_at | 3.57E-05 | 3.94E-07 | -5.85572 | 6.353495 | -1.2913 | JADE1 |
| 206852_at | 1.91E-02 | 1.49E-03 | -3.36732 | -1.4683 | -1.29109 | EPHA7 |
| 218796_at | 2.65E-02 | 2.35E-03 | -3.20976 | -1.89077 | -1.29071 | FERMT1 |
| 222162_s_at | 1.21E-01 | 2.39E-02 | -2.33102 | -3.98273 | -1.29069 | ADAMTS1 |
| 240951_at | 1.28E-02 | 8.71E-04 | -3.54713 | -0.97119 | -1.29055 | RORA |
| 213406_at | 3.45E-07 | 1.09E-09 | -7.51269 | 12.02102 | -1.29037 | WSB1 |
| 204260_at | 6.24E-02 | 8.07E-03 | -2.76197 | -3.01689 | -1.2895 | CHGB |
| 209541_at | 4.44E-02 | 4.91E-03 | -2.94695 | -2.56578 | -1.28872 | IGF1 |
| 218012_at | 4.51E-04 | 1.05E-05 | -4.91348 | 3.210455 | -1.28833 | TSPYL2 |
| 229125_at | 1.17E-07 | 2.66E-10 | -7.91261 | 13.37932 | -1.28826 | KANK4 |
| 228317_at | 2.06E-03 | 7.60E-05 | -4.32121 | 1.323908 | -1.28757 | ZFAND5 |
| 205921_s_at | 7.80E-03 | 4.43E-04 | -3.76828 | -0.33954 | -1.2862 | SLC6A6 |
| 224223_s_at | 8.83E-03 | 5.25E-04 | -3.7132 | -0.49884 | -1.28608 | PDE11A |
| 224458_at | 1.37E-03 | 4.49E-05 | -4.48105 | 1.823831 | -1.28573 | TMEM246 |
| 205945_at | 1.83E-02 | 1.41E-03 | -3.38642 | -1.41625 | -1.28328 | IL6R |
| 229991_s_at | 2.43E-03 | 9.37E-05 | -4.25708 | 1.125533 | -1.28307 | SYTL4 |
| 225354_s_at | 2.08E-03 | 7.70E-05 | -4.31693 | 1.310626 | -1.28249 | SH3BGRL2 |
| 223605_at | 2.18E-02 | 1.80E-03 | -3.30218 | -1.64451 | -1.28223 | SLC25A18 |
| 206453_s_at | 1.76E-06 | 8.61E-09 | -6.93113 | 10.03189 | -1.28029 | NDRG2 |
| 211075_s_at | 5.88E-04 | 1.48E-05 | -4.81246 | 2.882522 | -1.28002 | CD47 |
| 217998_at | 5.21E-03 | 2.56E-04 | -3.94316 | 0.174418 | -1.27899 | PHLDA1 |
| 212276_at | 2.96E-04 | 6.03E-06 | -5.07422 | 3.736681 | -1.27891 | LPIN1 |
| 232304_at | 2.22E-02 | 1.85E-03 | -3.29328 | -1.66842 | -1.27841 | AK026714 |
| 208491_s_at | 4.02E-02 | 4.24E-03 | -2.99976 | -2.43326 | -1.27809 | PGM5 |
| 218854_at | 5.44E-04 | 1.33E-05 | -4.84319 | 2.982052 | -1.27779 | DSE |
| 229175_at | 1.87E-05 | 1.69E-07 | -6.09569 | 7.170249 | -1.27377 | SMYD4 |
| 232464_at | 1.11E-02 | 7.13E-04 | -3.6131 | -0.78502 | -1.27347 | TRIM78P |
| 235308_at | 9.34E-05 | 1.39E-06 | -5.49639 | 5.140244 | -1.27313 | ZBTB20 |
| 207455_at | 1.04E-01 | 1.83E-02 | -2.44092 | -3.74838 | -1.2727 | P2RY1 |
| 244441_at | 8.23E-04 | 2.30E-05 | -4.68056 | 2.457849 | -1.27243 | USP31 |
| 225801_at | 2.76E-04 | 5.51E-06 | -5.10063 | 3.823626 | -1.27239 | FBXO32 |
| 227444_at | 9.67E-07 | 4.00E-09 | -7.14697 | 10.77144 | -1.2716 | ARMCX4 |
| 226507_at | 3.29E-03 | 1.40E-04 | -4.13382 | 0.747977 | -1.27024 | PAK1 |
| 231192_at | 1.70E-01 | 4.58E-02 | -2.04988 | -4.54236 | -1.26941 | LPAR3 |
| 200878_at | 5.58E-04 | 1.37E-05 | -4.83342 | 2.950375 | -1.26897 | EPAS1 |
| 215945_s_at | 2.33E-02 | 1.97E-03 | -3.27109 | -1.72785 | -1.26826 | TRIM2 |
| 204719_at | 5.39E-02 | 6.47E-03 | -2.84525 | -2.81637 | -1.26812 | ABCA8 |
| 204686_at | 7.47E-04 | 2.02E-05 | -4.71998 | 2.584341 | -1.26485 | IRS1 |
| 212094_at | 1.00E-03 | 2.98E-05 | -4.60337 | 2.211308 | -1.26433 | PEG10 |
| 209488_s_at | 3.59E-04 | 7.89E-06 | -4.99604 | 3.480116 | -1.26378 | RBPMS |
| 219134_at | 2.39E-02 | 2.04E-03 | -3.25848 | -1.76151 | -1.26349 | ELTD1 |
| 208453_s_at | 9.31E-04 | 2.70E-05 | -4.6334 | 2.307022 | -1.26345 | XPNPEP1 |
| 226682_at | 1.85E-02 | 1.44E-03 | -3.37947 | -1.43522 | -1.26343 | RORA |
| 206923_at | 3.50E-04 | 7.62E-06 | -5.00617 | 3.513262 | -1.26313 | PRKCA |
| 242945_at | 2.60E-03 | 1.02E-04 | -4.23072 | 1.044358 | -1.26268 | FAM20A |
| 228538_at | 1.04E-01 | 1.85E-02 | -2.43605 | -3.75893 | -1.26204 | ZNF662 |
| 242307_at | 6.81E-06 | 4.93E-08 | -6.44141 | 8.352592 | -1.26017 | ZNF789 |
| 208305_at | 2.15E-03 | 8.04E-05 | -4.30366 | 1.269488 | -1.25943 | PGR |
| 224202_at | 9.71E-02 | 1.63E-02 | -2.48783 | -3.64577 | -1.25909 | SUFU |
| 204037_at | 3.96E-04 | 8.84E-06 | -4.96286 | 3.371561 | -1.25792 | LPAR1 |
| 214721_x_at | 1.23E-04 | 1.97E-06 | -5.39726 | 4.808226 | -1.2574 | CDC42EP4 |
| 219064_at | 6.75E-03 | 3.64E-04 | -3.83125 | -0.15589 | -1.25731 | ITIH5 |
| 240797_at | 1.12E-03 | 3.42E-05 | -4.56302 | 2.083047 | -1.25668 | PERP |
| 217997_at | 2.07E-02 | 1.66E-03 | -3.33029 | -1.56873 | -1.25638 | PHLDA1 |
| 204622_x_at | 1.11E-01 | 2.05E-02 | -2.39545 | -3.84637 | -1.25625 | NR4A2 |
| 1554512_a_at | 1.03E-04 | 1.59E-06 | -5.45832 | 5.012604 | -1.25591 | CEP89 |
| 229716_at | 2.37E-05 | 2.32E-07 | -6.00533 | 6.862207 | -1.2541 | UBXN10-AS1 |
| 217897_at | 1.51E-03 | 5.06E-05 | -4.44467 | 1.709377 | -1.25409 | FXYD6 |
| 227623_at | 5.64E-02 | 6.90E-03 | -2.82103 | -2.87513 | -1.25289 | CACNA2D1 |
| 205548_s_at | 2.84E-03 | 1.15E-04 | -4.19383 | 0.931144 | -1.2527 | BTG3 |
| 230087_at | 9.53E-04 | 2.77E-05 | -4.62533 | 2.28128 | -1.25131 | PRIMA1 |
| 225482_at | 6.24E-03 | 3.27E-04 | -3.86567 | -0.05481 | -1.25064 | KIF1A |
| 1557089_at | 8.02E-03 | 4.61E-04 | -3.75577 | -0.37583 | -1.24993 | KIF5C |
| 226623_at | 1.66E-03 | 5.80E-05 | -4.40318 | 1.57932 | -1.24935 | PHYHIPL |
| 242881_x_at | 1.31E-01 | 2.77E-02 | -2.26878 | -4.11165 | -1.24915 | BC017398 |
| 229649_at | 4.49E-03 | 2.11E-04 | -4.00443 | 0.35728 | -1.24875 | NRXN3 |
| 228553_at | 2.07E-06 | 1.05E-08 | -6.8763 | 9.843889 | -1.24685 | ENAH |
| 209184_s_at | 2.03E-03 | 7.47E-05 | -4.32616 | 1.33927 | -1.24668 | IRS2 |
| 207327_at | 8.65E-05 | 1.26E-06 | -5.52514 | 5.23678 | -1.24436 | EYA4 |
| 223217_s_at | 6.96E-02 | 9.55E-03 | -2.69804 | -3.1679 | -1.24423 | NFKBIZ |
| 232139_s_at | 3.49E-04 | 7.57E-06 | -5.0081 | 3.519591 | -1.24395 | KIAA1919 |
| 242218_at | 4.99E-04 | 1.20E-05 | -4.87439 | 3.083306 | -1.24311 | PPARD |
| 203610_s_at | 7.42E-04 | 2.00E-05 | -4.72308 | 2.594287 | -1.24169 | TRIM38 |
| 232847_at | 9.97E-02 | 1.71E-02 | -2.46871 | -3.68777 | -1.24145 | SALL3 |
| 206576_s_at | 3.66E-02 | 3.71E-03 | -3.04812 | -2.31051 | -1.24084 | CEACAM1 |
| 203453_at | 1.51E-01 | 3.63E-02 | -2.15334 | -4.34327 | -1.23984 | SCNN1A |
| 1564796_at | 4.29E-02 | 4.66E-03 | -2.9659 | -2.51841 | -1.23922 | EMP1 |
| 211343_s_at | 3.05E-03 | 1.27E-04 | -4.1636 | 0.838726 | -1.23846 | COL13A1 |
| 224368_s_at | 1.17E-03 | 3.62E-05 | -4.54529 | 2.026811 | -1.23768 | NDRG3 |
| 241789_at | 5.78E-06 | 3.94E-08 | -6.50435 | 8.568287 | -1.23671 | RBMS3 |
| 210639_s_at | 3.44E-05 | 3.74E-07 | -5.87034 | 6.403136 | -1.23666 | ATG5 |
| 208816_x_at | 6.27E-05 | 8.31E-07 | -5.64413 | 5.63738 | -1.23561 | ANXA2P2 |
| 208581_x_at | 7.13E-02 | 9.93E-03 | -2.68294 | -3.2032 | -1.23543 | MT1X |
| 227556_at | 7.80E-03 | 4.44E-04 | -3.76803 | -0.34027 | -1.23523 | NME7 |
| 206461_x_at | 1.20E-01 | 2.36E-02 | -2.33679 | -3.97063 | -1.23502 | MT1H |
| 219230_at | 6.26E-02 | 8.11E-03 | -2.76015 | -3.02122 | -1.23434 | TMEM100 |
| 235813_at | 1.80E-03 | 6.37E-05 | -4.3747 | 1.490363 | -1.23429 | LOC101927027 |
| 244553_at | 2.64E-03 | 1.04E-04 | -4.22339 | 1.021818 | -1.23329 | HOTTIP |
| 211883_x_at | 4.45E-02 | 4.93E-03 | -2.9454 | -2.56964 | -1.23324 | CEACAM1 |
| 236561_at | 4.25E-02 | 4.59E-03 | -2.97166 | -2.50398 | -1.23108 | TGFBR1 |
| 227860_at | 1.43E-03 | 4.71E-05 | -4.46606 | 1.77663 | -1.23068 | CPXM1 |
| 205221_at | 5.14E-02 | 6.05E-03 | -2.87 | -2.75594 | -1.22925 | HGD |
| 232300_at | 1.47E-03 | 4.90E-05 | -4.45454 | 1.740393 | -1.22922 | ADIRF-AS1 |
| 201627_s_at | 2.66E-02 | 2.36E-03 | -3.20765 | -1.89634 | -1.22837 | INSIG1 |
| 203571_s_at | 1.37E-02 | 9.49E-04 | -3.51859 | -1.05111 | -1.22824 | ADIRF |
| 212254_s_at | 1.45E-05 | 1.23E-07 | -6.18484 | 7.474614 | -1.22767 | DST |
| 212996_s_at | 2.95E-03 | 1.21E-04 | -4.17751 | 0.881235 | -1.22635 | URB1 |
| 214508_x_at | 7.80E-03 | 4.44E-04 | -3.76802 | -0.3403 | -1.22525 | CREM |
| 220816_at | 1.78E-01 | 4.98E-02 | -2.01195 | -4.61329 | -1.22518 | LPAR3 |
| 204597_x_at | 1.08E-02 | 6.88E-04 | -3.62509 | -0.75098 | -1.22455 | STC1 |
| 209784_s_at | 1.24E-03 | 3.93E-05 | -4.52096 | 1.94979 | -1.22447 | JAG2 |
| 1552670_a_at | 1.43E-02 | 1.00E-03 | -3.49981 | -1.10349 | -1.21997 | PPP1R3B |
| 229313_at | 5.58E-04 | 1.37E-05 | -4.8337 | 2.951272 | -1.21982 | ANO5 |
| 206408_at | 9.69E-03 | 5.95E-04 | -3.67238 | -0.61607 | -1.21942 | LRRTM2 |
| 216321_s_at | 4.92E-04 | 1.17E-05 | -4.88066 | 3.103688 | -1.2191 | NR3C1 |
| 1556181_at | 2.80E-03 | 1.13E-04 | -4.19983 | 0.949537 | -1.21891 | ANKRD65 |
| 227835_at | 8.27E-02 | 1.26E-02 | -2.59166 | -3.41338 | -1.21857 | LOC389831 |
| 238575_at | 9.72E-03 | 5.98E-04 | -3.67095 | -0.62015 | -1.21797 | OSBPL6 |
| 1554791_a_at | 2.43E-03 | 9.36E-05 | -4.25724 | 1.126014 | -1.2175 | KANSL1L |
| 201425_at | 4.07E-03 | 1.85E-04 | -4.04548 | 0.480585 | -1.2169 | ALDH2 |
| 219340_s_at | 5.82E-03 | 2.99E-04 | -3.8948 | 0.031083 | -1.21609 | CLN8 |
| 211171_s_at | 1.25E-03 | 3.95E-05 | -4.51969 | 1.945778 | -1.21592 | PDE10A |
| 213572_s_at | 9.59E-03 | 5.86E-04 | -3.67759 | -0.60115 | -1.21453 | SERPINB1 |
| 233011_at | 2.61E-03 | 1.03E-04 | -4.22829 | 1.036885 | -1.21419 | ANXA1 |
| 205383_s_at | 8.87E-05 | 1.31E-06 | -5.51378 | 5.198638 | -1.21403 | ZBTB20 |
| 205564_at | 1.47E-01 | 3.47E-02 | -2.17195 | -4.30659 | -1.21203 | PAGE4 |
| 235231_at | 1.10E-04 | 1.72E-06 | -5.43611 | 4.93821 | -1.21198 | ZNF789 |
| 1556069_s_at | 6.95E-03 | 3.80E-04 | -3.81745 | -0.19627 | -1.21185 | HIF3A |
| 217744_s_at | 5.45E-04 | 1.33E-05 | -4.84222 | 2.978906 | -1.2106 | PERP |
| 225481_at | 4.52E-04 | 1.05E-05 | -4.91241 | 3.206997 | -1.21039 | FRMD6 |
| 201626_at | 2.69E-02 | 2.41E-03 | -3.20031 | -1.91568 | -1.21006 | INSIG1 |
| 209615_s_at | 1.21E-02 | 8.03E-04 | -3.574 | -0.8956 | -1.21003 | PAK1 |
| 202831_at | 7.86E-02 | 1.16E-02 | -2.62411 | -3.33928 | -1.20973 | GCC2 |
| 1559957_a_at | 3.39E-02 | 3.33E-03 | -3.08677 | -2.21144 | -1.20863 | LOC642852 |
| 239568_at | 1.85E-06 | 9.30E-09 | -6.90969 | 9.958373 | -1.20782 | PLEKHH2 |
| 226145_s_at | 9.84E-02 | 1.67E-02 | -2.478 | -3.66738 | -1.20584 | FRAS1 |
| 223753_s_at | 9.97E-03 | 6.18E-04 | -3.66045 | -0.65018 | -1.20579 | CFC1 |
| 219229_at | 3.76E-03 | 1.67E-04 | -4.07838 | 0.57984 | -1.20565 | SLCO3A1 |
| 1555210_at | 3.84E-03 | 1.71E-04 | -4.07026 | 0.555331 | -1.20304 | DTWD1 |
| 214604_at | 6.59E-06 | 4.71E-08 | -6.45401 | 8.395766 | -1.20304 | HOXD11 |
| 60815_at | 1.14E-02 | 7.44E-04 | -3.59922 | -0.82435 | -1.20282 | POLR2J4 |
| 201565_s_at | 2.06E-03 | 7.61E-05 | -4.32059 | 1.321984 | -1.20223 | ID2 |
| 208792_s_at | 1.17E-02 | 7.73E-04 | -3.58684 | -0.85935 | -1.2007 | CLU |
| 1569353_at | 1.37E-02 | 9.46E-04 | -3.51966 | -1.04813 | -1.19984 | CCP110 |
| 228188_at | 9.99E-04 | 2.96E-05 | -4.60558 | 2.218345 | -1.19907 | FOSL2 |
| 219759_at | 1.37E-01 | 3.03E-02 | -2.23064 | -4.18926 | -1.19825 | ERAP2 |
| 205443_at | 1.19E-02 | 7.92E-04 | -3.5785 | -0.88289 | -1.19822 | SNAPC1 |
| 213994_s_at | 6.82E-02 | 9.25E-03 | -2.7104 | -3.1389 | -1.19804 | SPON1 |
| 1559174_at | 1.25E-03 | 3.95E-05 | -4.51963 | 1.945579 | -1.1977 | RAB6A |
| 222917_s_at | 1.02E-02 | 6.39E-04 | -3.6495 | -0.68147 | -1.19733 | TBX3 |
| 204429_s_at | 4.52E-05 | 5.31E-07 | -5.77105 | 6.066417 | -1.19584 | SLC2A5 |
| 218667_at | 1.41E-03 | 4.62E-05 | -4.47198 | 1.795268 | -1.19476 | PJA1 |
| 202393_s_at | 1.17E-02 | 7.73E-04 | -3.58676 | -0.85959 | -1.19282 | KLF10 |
| 204284_at | 1.63E-03 | 5.62E-05 | -4.41266 | 1.608994 | -1.19215 | PPP1R3C |
| 228531_at | 1.57E-02 | 1.14E-03 | -3.45698 | -1.22235 | -1.19179 | SAMD9 |
| 201108_s_at | 6.55E-02 | 8.72E-03 | -2.73287 | -3.08596 | -1.19148 | THBS1 |
| 210762_s_at | 6.19E-03 | 3.23E-04 | -3.86953 | -0.04346 | -1.19136 | DLC1 |
| 228819_at | 1.28E-04 | 2.09E-06 | -5.38061 | 4.752614 | -1.19114 | TSPAN18 |
| 204294_at | 1.08E-02 | 6.87E-04 | -3.62534 | -0.75025 | -1.19069 | AMT |
| 213657_s_at | 4.04E-03 | 1.84E-04 | -4.04827 | 0.488985 | -1.19067 | AK055981 |
| 204780_s_at | 4.03E-02 | 4.26E-03 | -2.99878 | -2.43572 | -1.19065 | FAS |
| 204975_at | 7.13E-04 | 1.90E-05 | -4.73794 | 2.642086 | -1.19013 | EMP2 |
| 214753_at | 3.08E-02 | 2.90E-03 | -3.13591 | -2.0843 | -1.18993 | N4BP2L2 |
| 219737_s_at | 8.32E-04 | 2.33E-05 | -4.6768 | 2.445786 | -1.18886 | PCDH9 |
| 213134_x_at | 4.46E-03 | 2.09E-04 | -4.008 | 0.367995 | -1.18867 | BTG3 |
| 226570_at | 2.28E-03 | 8.71E-05 | -4.27932 | 1.194186 | -1.18826 | ATP1B3 |
| 1554830_a_at | 3.34E-04 | 7.16E-06 | -5.02437 | 3.572952 | -1.18779 | STEAP3 |
| 234351_x_at | 9.70E-03 | 5.97E-04 | -3.6716 | -0.6183 | -1.18758 | TRPS1 |
| 233059_at | 1.46E-01 | 3.39E-02 | -2.18245 | -4.28579 | -1.18737 | KCNJ3 |
| 32540_at | 1.50E-04 | 2.54E-06 | -5.32403 | 4.563863 | -1.1873 | PPP3CC |
| 201650_at | 1.44E-01 | 3.32E-02 | -2.19187 | -4.26705 | -1.18648 | KRT19 |
| 220813_at | 1.06E-02 | 6.74E-04 | -3.63171 | -0.73215 | -1.18575 | CYSLTR2 |
| 1554494_at | 8.52E-02 | 1.32E-02 | -2.57287 | -3.45596 | -1.18369 | MTHFSD |
| 1561039_a_at | 4.31E-03 | 2.00E-04 | -4.02203 | 0.41009 | -1.18351 | ZNF81 |
| 210942_s_at | 2.58E-03 | 1.01E-04 | -4.234 | 1.054437 | -1.18286 | ST3GAL6 |
| 209909_s_at | 9.19E-03 | 5.54E-04 | -3.69566 | -0.54929 | -1.18256 | TGFB2 |
| 202003_s_at | 1.37E-03 | 4.49E-05 | -4.48046 | 1.821964 | -1.18209 | ACAA2 |
| 211124_s_at | 1.21E-03 | 3.78E-05 | -4.53261 | 1.986637 | -1.18054 | KITLG |
| 39249_at | 6.93E-03 | 3.78E-04 | -3.81967 | -0.18977 | -1.18038 | AQP3 |
| 238285_at | 9.27E-03 | 5.61E-04 | -3.69192 | -0.56003 | -1.1793 | SOX5 |
| 235228_at | 2.03E-03 | 7.45E-05 | -4.32722 | 1.342572 | -1.17912 | CCDC85A |
| 238669_at | 1.34E-02 | 9.21E-04 | -3.52869 | -1.02287 | -1.17837 | PTGS1 |
| 219194_at | 9.74E-02 | 1.64E-02 | -2.48571 | -3.65045 | -1.17769 | SEMA4G |
| 235897_at | 8.75E-03 | 5.18E-04 | -3.71788 | -0.48536 | -1.17721 | COPZ2 |
| 218839_at | 6.32E-03 | 3.33E-04 | -3.86026 | -0.07074 | -1.17624 | HEY1 |
| 206298_at | 1.18E-04 | 1.86E-06 | -5.41352 | 4.862609 | -1.17616 | ARHGAP22 |
| 222870_s_at | 1.11E-02 | 7.15E-04 | -3.61233 | -0.7872 | -1.17549 | B3GNT2 |
| 1556235_at | 4.46E-06 | 2.79E-08 | -6.60138 | 8.900946 | -1.17493 | LOC101927468 |
| 1559883_s_at | 1.40E-03 | 4.61E-05 | -4.47255 | 1.797039 | -1.17421 | SAMHD1 |
| 241824_at | 7.54E-03 | 4.24E-04 | -3.78247 | -0.2983 | -1.174 | RP11-373D23.2 |
| 242163_at | 4.75E-02 | 5.42E-03 | -2.91069 | -2.65582 | -1.17372 | THRAP3 |
| 212759_s_at | 1.87E-03 | 6.71E-05 | -4.35918 | 1.441979 | -1.17372 | TCF7L2 |
| 220974_x_at | 1.20E-03 | 3.75E-05 | -4.53517 | 1.994756 | -1.17336 | SFXN3 |
| 211018_at | 1.75E-02 | 1.32E-03 | -3.40683 | -1.3604 | -1.1731 | LSS |
| 212242_at | 3.00E-04 | 6.17E-06 | -5.06782 | 3.715628 | -1.17293 | TUBA4A |
| 226800_at | 3.62E-03 | 1.59E-04 | -4.09357 | 0.625794 | -1.17279 | EFCAB7 |
| 207124_s_at | 1.05E-02 | 6.62E-04 | -3.63764 | -0.71525 | -1.17198 | GNB5 |
| 205206_at | 1.71E-02 | 1.28E-03 | -3.41921 | -1.32644 | -1.17174 | KAL1 |
| 215501_s_at | 3.44E-03 | 1.49E-04 | -4.1137 | 0.686835 | -1.17071 | DUSP10 |
| 220306_at | 4.04E-02 | 4.28E-03 | -2.99708 | -2.44003 | -1.16976 | FAM46C |
| 215569_at | 9.84E-07 | 4.12E-09 | -7.13816 | 10.7413 | -1.16857 | GTF2IRD2B |
| 207284_s_at | 3.18E-02 | 3.04E-03 | -3.119 | -2.12819 | -1.16843 | ASPH |
| 204249_s_at | 1.73E-03 | 6.08E-05 | -4.38886 | 1.534573 | -1.16819 | LMO2 |
| 219882_at | 3.69E-02 | 3.74E-03 | -3.04489 | -2.31874 | -1.16738 | TTLL7 |
| 229820_at | 4.02E-02 | 4.24E-03 | -2.99993 | -2.43284 | -1.16672 | LINC00969 |
| 203979_at | 4.87E-02 | 5.62E-03 | -2.89705 | -2.68949 | -1.16652 | CYP27A1 |
| 240435_at | 2.06E-03 | 7.57E-05 | -4.32219 | 1.326959 | -1.16514 | LOC101928635 |
| 219916_s_at | 3.28E-02 | 3.18E-03 | -3.10306 | -2.16943 | -1.16466 | RNF39 |
| 218864_at | 6.93E-03 | 3.78E-04 | -3.81951 | -0.19025 | -1.16325 | TNS1 |
| 237577_at | 3.33E-03 | 1.41E-04 | -4.12982 | 0.735826 | -1.16198 | PCNP |
| 212070_at | 8.86E-04 | 2.52E-05 | -4.65333 | 2.370703 | -1.16125 | GPR56 |
| 1555058_a_at | 2.35E-03 | 8.99E-05 | -4.26969 | 1.16442 | -1.16115 | LPGAT1 |
| 210613_s_at | 2.26E-02 | 1.90E-03 | -3.28386 | -1.69368 | -1.15956 | SYNGR1 |
| 205573_s_at | 1.74E-02 | 1.31E-03 | -3.41088 | -1.3493 | -1.15937 | SNX7 |
| 226960_at | 9.47E-02 | 1.56E-02 | -2.50572 | -3.60624 | -1.15916 | CXCL17 |
| 216887_s_at | 1.84E-02 | 1.42E-03 | -3.38409 | -1.4226 | -1.15899 | LDB3 |
| 223832_s_at | 1.51E-02 | 1.09E-03 | -3.47264 | -1.17901 | -1.15854 | CAPNS2 |
| 213355_at | 1.39E-03 | 4.55E-05 | -4.47682 | 1.810495 | -1.157 | ST3GAL6 |
| 235352_at | 9.05E-04 | 2.60E-05 | -4.64416 | 2.341385 | -1.15692 | MR1 |
| 219103_at | 2.67E-02 | 2.39E-03 | -3.20397 | -1.90605 | -1.15583 | ASAP3 |
| 241600_at | 5.34E-02 | 6.39E-03 | -2.84978 | -2.80533 | -1.15572 | WIPF3 |
| 227434_at | 4.49E-03 | 2.12E-04 | -4.00376 | 0.355273 | -1.15569 | WBSCR17 |
| 203066_at | 1.34E-02 | 9.22E-04 | -3.52822 | -1.02419 | -1.15497 | CHST15 |
| 33322_i_at | 3.22E-02 | 3.09E-03 | -3.11321 | -2.14319 | -1.15423 | SFN |
| 202554_s_at | 1.29E-02 | 8.82E-04 | -3.54291 | -0.98303 | -1.15399 | GSTM3 |
| 228568_at | 6.34E-03 | 3.35E-04 | -3.8584 | -0.07621 | -1.15331 | MYZAP |
| 216350_s_at | 2.26E-03 | 8.60E-05 | -4.2833 | 1.206482 | -1.15317 | ZNF10 |
| 238513_at | 1.27E-02 | 8.57E-04 | -3.55271 | -0.95552 | -1.15231 | PRRG4 |
| 205303_at | 2.11E-02 | 1.71E-03 | -3.3194 | -1.59812 | -1.15213 | KCNJ8 |
| 208881_x_at | 1.30E-03 | 4.17E-05 | -4.5033 | 1.894006 | -1.15143 | IDI1 |
| 224393_s_at | 4.03E-02 | 4.25E-03 | -2.99909 | -2.43494 | -1.15055 | CECR6 |
| 1558604_a_at | 4.68E-04 | 1.10E-05 | -4.89948 | 3.164887 | -1.15013 | SSBP2 |
| 1562367_at | 1.35E-04 | 2.22E-06 | -5.36283 | 4.693235 | -1.14718 | C15orf54 |
| 204368_at | 6.63E-03 | 3.55E-04 | -3.83957 | -0.13149 | -1.1468 | SLCO2A1 |
| 224013_s_at | 8.17E-04 | 2.28E-05 | -4.68301 | 2.465687 | -1.14636 | SOX7 |
| 222730_s_at | 6.85E-04 | 1.81E-05 | -4.7528 | 2.689927 | -1.14555 | ZDHHC2 |
| 227748_at | 5.96E-03 | 3.07E-04 | -3.88552 | 0.003671 | -1.1454 | RBMXL1 |
| 219670_at | 4.77E-05 | 5.71E-07 | -5.75053 | 5.996952 | -1.14432 | BEND5 |
| 239883_s_at | 6.19E-03 | 3.24E-04 | -3.86908 | -0.04476 | -1.14421 | ANO4 |
| 235867_at | 4.59E-02 | 5.16E-03 | -2.92851 | -2.61167 | -1.14265 | GSTM3 |
| 228325_at | 1.00E-01 | 1.72E-02 | -2.46626 | -3.69313 | -1.14217 | SPIDR |
| 203585_at | 3.20E-02 | 3.08E-03 | -3.11479 | -2.13911 | -1.14212 | ZNF185 |
| 244057_s_at | 1.94E-02 | 1.53E-03 | -3.35847 | -1.49236 | -1.14209 | VSTM4 |
| 238784_at | 1.23E-01 | 2.46E-02 | -2.31895 | -4.00795 | -1.14182 | DPY19L2 |
| 225611_at | 5.04E-03 | 2.45E-04 | -3.95751 | 0.217118 | -1.14076 | MAST4 |
| 220701_at | 1.76E-02 | 1.33E-03 | -3.40479 | -1.366 | -1.14066 | LINC00216 |
| 208791_at | 2.39E-02 | 2.04E-03 | -3.25817 | -1.76231 | -1.1397 | CLU |
| 218062_x_at | 2.26E-05 | 2.19E-07 | -6.02162 | 6.917697 | -1.13961 | CDC42EP4 |
| 210880_s_at | 1.43E-04 | 2.41E-06 | -5.33986 | 4.616623 | -1.13961 | EFS |
| 205051_s_at | 4.37E-03 | 2.03E-04 | -4.01757 | 0.396686 | -1.13957 | KIT |
| 203186_s_at | 2.94E-03 | 1.21E-04 | -4.17888 | 0.88541 | -1.13949 | S100A4 |
| 209465_x_at | 5.05E-02 | 5.90E-03 | -2.8793 | -2.73316 | -1.13933 | PTN |
| 227074_at | 1.01E-01 | 1.74E-02 | -2.46104 | -3.70456 | -1.13897 | LOC100131564 |
| 209436_at | 2.18E-02 | 1.80E-03 | -3.30168 | -1.64585 | -1.13862 | SPON1 |
| 239757_at | 3.35E-04 | 7.20E-06 | -5.02294 | 3.568256 | -1.13837 | ZFAND6 |
| 239336_at | 7.86E-02 | 1.16E-02 | -2.6241 | -3.33931 | -1.1379 | THBS1 |
| 213950_s_at | 3.31E-04 | 7.08E-06 | -5.02781 | 3.58423 | -1.1376 | PPP3CC |
| 229764_at | 6.37E-03 | 3.36E-04 | -3.85684 | -0.08078 | -1.13734 | TPRG1 |
| 209185_s_at | 2.09E-02 | 1.68E-03 | -3.32599 | -1.58034 | -1.13709 | IRS2 |
| 203562_at | 6.19E-05 | 8.15E-07 | -5.64965 | 5.656017 | -1.13709 | FEZ1 |
| 1558135_at | 3.61E-03 | 1.58E-04 | -4.09497 | 0.630047 | -1.13683 | TAF11 |
| 214734_at | 3.90E-04 | 8.67E-06 | -4.96861 | 3.390374 | -1.13536 | EXPH5 |
| 223748_at | 2.36E-02 | 2.00E-03 | -3.26513 | -1.74376 | -1.13503 | SLC4A11 |
| 219639_x_at | 2.60E-02 | 2.29E-03 | -3.21912 | -1.86602 | -1.13443 | PARP6 |
| 204085_s_at | 2.44E-03 | 9.45E-05 | -4.2542 | 1.116635 | -1.13442 | CLN5 |
| 228215_at | 5.08E-05 | 6.20E-07 | -5.72701 | 5.917371 | -1.13393 | ADD3-AS1 |
| 210426_x_at | 3.41E-03 | 1.47E-04 | -4.1185 | 0.701406 | -1.13365 | RORA |
| 227742_at | 2.30E-02 | 1.94E-03 | -3.2758 | -1.71525 | -1.13351 | CLIC6 |
| 1557418_at | 6.32E-03 | 3.33E-04 | -3.85969 | -0.0724 | -1.13257 | ACSL4 |
| 219936_s_at | 2.28E-03 | 8.68E-05 | -4.28054 | 1.197962 | -1.1324 | GPR87 |
| 205823_at | 1.52E-05 | 1.32E-07 | -6.16408 | 7.403705 | -1.13203 | RGS12 |
| 211919_s_at | 1.60E-01 | 4.08E-02 | -2.10082 | -4.44534 | -1.13167 | CXCR4 |
| 200952_s_at | 5.01E-04 | 1.20E-05 | -4.8725 | 3.077157 | -1.13097 | CCND2 |
| 231941_s_at | 2.96E-02 | 2.75E-03 | -3.15497 | -2.03463 | -1.13082 | MUC20 |
| 235277_at | 7.87E-04 | 2.17E-05 | -4.69826 | 2.514591 | -1.13064 | AMOTL1 |
| 215388_s_at | 9.65E-03 | 5.92E-04 | -3.67419 | -0.61087 | -1.12983 | CFH |
| 202735_at | 3.34E-03 | 1.43E-04 | -4.12739 | 0.728423 | -1.12979 | EBP |
| 205404_at | 1.33E-02 | 9.16E-04 | -3.53041 | -1.01806 | -1.12902 | HSD11B1 |
| 231643_s_at | 3.06E-04 | 6.34E-06 | -5.05978 | 3.689193 | -1.12893 | CMIP |
| 218285_s_at | 6.75E-03 | 3.64E-04 | -3.83124 | -0.1559 | -1.12885 | BDH2 |
| 226878_at | 6.40E-03 | 3.39E-04 | -3.85453 | -0.08758 | -1.12778 | HLA-DOA |
| 205934_at | 2.40E-02 | 2.05E-03 | -3.25711 | -1.76516 | -1.1273 | PLCL1 |
| 203770_s_at | 9.63E-04 | 2.82E-05 | -4.62049 | 2.265859 | -1.12725 | STS |
| 235105_at | 1.74E-02 | 1.31E-03 | -3.40962 | -1.35277 | -1.12678 | MED28 |
| 226622_at | 9.24E-03 | 5.59E-04 | -3.6931 | -0.55664 | -1.12676 | MUC20 |
| 228108_at | 7.10E-03 | 3.92E-04 | -3.80821 | -0.22326 | -1.1267 | PPM1L |
| 33323_r_at | 4.61E-02 | 5.18E-03 | -2.9269 | -2.61567 | -1.12649 | SFN |
| 226811_at | 1.31E-02 | 8.97E-04 | -3.53731 | -0.99874 | -1.12646 | FAM46C |
| 205251_at | 4.48E-03 | 2.11E-04 | -4.00528 | 0.359826 | -1.12595 | PER2 |
| 226899_at | 2.13E-02 | 1.73E-03 | -3.31511 | -1.60969 | -1.12537 | UNC5B |
| 209167_at | 4.58E-03 | 2.18E-04 | -3.99514 | 0.329487 | -1.12474 | GPM6B |
| 218298_s_at | 2.24E-02 | 1.87E-03 | -3.28836 | -1.68162 | -1.12425 | C14orf159 |
| 226462_at | 1.13E-01 | 2.14E-02 | -2.37789 | -3.88381 | -1.12394 | STXBP6 |
| 1556182_x_at | 3.40E-03 | 1.46E-04 | -4.12034 | 0.706987 | -1.12361 | ANKRD65 |
| 201397_at | 4.84E-06 | 3.13E-08 | -6.56868 | 8.78883 | -1.12325 | PHGDH |
| 232174_at | 1.34E-02 | 9.23E-04 | -3.52785 | -1.02521 | -1.12082 | AK025288 |
| 232113_at | 9.60E-02 | 1.60E-02 | -2.49666 | -3.62629 | -1.12001 | AK021804 |
| 215561_s_at | 1.74E-02 | 1.31E-03 | -3.41051 | -1.35032 | -1.11926 | IL1R1 |
| 216042_at | 3.74E-02 | 3.82E-03 | -3.03741 | -2.33781 | -1.1186 | TNFRSF25 |
| 212253_x_at | 7.78E-03 | 4.41E-04 | -3.76975 | -0.33526 | -1.11819 | DST |
| 215133_s_at | 4.01E-02 | 4.22E-03 | -3.00151 | -2.42882 | -1.11816 | FAM153A |
| 224940_s_at | 4.62E-02 | 5.21E-03 | -2.92518 | -2.61993 | -1.11812 | PAPPA |
| 202695_s_at | 3.72E-02 | 3.79E-03 | -3.04096 | -2.32876 | -1.11777 | STK17A |
| 218143_s_at | 6.35E-02 | 8.28E-03 | -2.7523 | -3.0399 | -1.11755 | SCAMP2 |
| 209201_x_at | 1.38E-01 | 3.05E-02 | -2.22788 | -4.19484 | -1.11747 | CXCR4 |
| 216174_at | 7.72E-03 | 4.36E-04 | -3.77325 | -0.32511 | -1.11668 | HCRP1 |
| 236255_at | 2.00E-05 | 1.87E-07 | -6.06605 | 7.069146 | -1.11642 | PLEKHG4B |
| 203394_s_at | 1.19E-01 | 2.34E-02 | -2.34029 | -3.96329 | -1.11586 | HES1 |
| 65585_at | 9.01E-03 | 5.41E-04 | -3.70376 | -0.526 | -1.11422 | FAM86B1 |
| 211922_s_at | 9.85E-04 | 2.91E-05 | -4.61117 | 2.236154 | -1.11308 | CAT |
| 204615_x_at | 1.86E-03 | 6.65E-05 | -4.36186 | 1.450325 | -1.11141 | IDI1 |
| 242752_at | 1.42E-02 | 9.97E-04 | -3.50201 | -1.09736 | -1.11106 | PPM1K |
| 207332_s_at | 1.93E-02 | 1.51E-03 | -3.36193 | -1.48296 | -1.11078 | TFRC |
| 212420_at | 2.05E-04 | 3.78E-06 | -5.2098 | 4.184294 | -1.11065 | ELF1 |
| 224435_at | 3.26E-03 | 1.38E-04 | -4.13798 | 0.760649 | -1.10989 | FAM213A |
| 213822_s_at | 7.24E-02 | 1.02E-02 | -2.67413 | -3.22372 | -1.10981 | UBE3B |
| 213001_at | 3.08E-02 | 2.91E-03 | -3.13441 | -2.0882 | -1.10961 | ANGPTL2 |
| 235471_at | 1.36E-02 | 9.38E-04 | -3.52259 | -1.03993 | -1.10931 | VSTM4 |
| 214453_s_at | 1.20E-01 | 2.38E-02 | -2.33275 | -3.97911 | -1.109 | IFI44 |
| 230369_at | 1.54E-03 | 5.20E-05 | -4.43623 | 1.682874 | -1.10858 | GPR161 |
| 205007_s_at | 2.34E-04 | 4.48E-06 | -5.16087 | 4.022383 | -1.1079 | CIB2 |
| 232311_at | 7.84E-03 | 4.46E-04 | -3.76629 | -0.34531 | -1.10687 | B2M |
| 231867_at | 6.43E-02 | 8.43E-03 | -2.74573 | -3.0555 | -1.10607 | TENM2 |
| 203015_s_at | 4.92E-04 | 1.17E-05 | -4.88008 | 3.101783 | -1.10597 | SSX2IP |
| 228942_s_at | 4.31E-03 | 1.99E-04 | -4.02311 | 0.413332 | -1.1055 | DAB2IP |
| 238463_at | 1.91E-05 | 1.73E-07 | -6.0877 | 7.14298 | -1.10513 | LOC100506834 |
| 243456_at | 3.23E-02 | 3.12E-03 | -3.11009 | -2.15128 | -1.1045 | ZNF214 |
| 1555600_s_at | 4.19E-02 | 4.49E-03 | -2.97903 | -2.48548 | -1.10421 | APOL4 |
| 227410_at | 4.11E-02 | 4.38E-03 | -2.98857 | -2.46147 | -1.10353 | FAM43A |
| 225079_at | 9.30E-04 | 2.69E-05 | -4.63387 | 2.30854 | -1.10288 | EMP2 |
| 210764_s_at | 1.69E-01 | 4.55E-02 | -2.05218 | -4.53801 | -1.10175 | CYR61 |
| 210405_x_at | 1.52E-02 | 1.10E-03 | -3.46995 | -1.18645 | -1.10125 | TNFRSF10B |
| 239321_at | 1.63E-02 | 1.20E-03 | -3.4395 | -1.27062 | -1.10101 | LOC441454 |
| 208092_s_at | 2.00E-02 | 1.58E-03 | -3.34641 | -1.52511 | -1.10039 | FAM49A |
| 209016_s_at | 4.29E-02 | 4.65E-03 | -2.96628 | -2.51746 | -1.09965 | KRT7 |
| 226252_at | 2.69E-06 | 1.45E-08 | -6.7849 | 9.530385 | -1.09896 | ZBTB20 |
| 230130_at | 2.95E-02 | 2.73E-03 | -3.1565 | -2.03064 | -1.09855 | SLIT2 |
| 233814_at | 7.31E-02 | 1.03E-02 | -2.6672 | -3.23981 | -1.09821 | EFNA5 |
| 228933_at | 7.27E-03 | 4.04E-04 | -3.79776 | -0.25377 | -1.0976 | NHS |
| 202341_s_at | 1.66E-02 | 1.23E-03 | -3.43155 | -1.2925 | -1.09759 | TRIM2 |
| 225778_at | 2.77E-03 | 1.11E-04 | -4.20474 | 0.964588 | -1.09729 | RBMS2 |
| 232392_at | 5.08E-03 | 2.48E-04 | -3.95427 | 0.20747 | -1.09688 | SRSF3 |
| 1559078_at | 1.26E-04 | 2.05E-06 | -5.38607 | 4.770858 | -1.09668 | AL833181 |
| 233324_at | 1.01E-02 | 6.31E-04 | -3.65351 | -0.67002 | -1.09591 | TRERF1 |
| 217644_s_at | 4.76E-04 | 1.12E-05 | -4.89297 | 3.143691 | -1.09582 | SOS2 |
| 201843_s_at | 2.44E-02 | 2.10E-03 | -3.24946 | -1.78551 | -1.09438 | EFEMP1 |
| 221031_s_at | 8.74E-02 | 1.37E-02 | -2.55665 | -3.49254 | -1.09427 | APOLD1 |
| 224796_at | 2.87E-04 | 5.80E-06 | -5.08565 | 3.774283 | -1.09377 | ASAP1 |
| 202157_s_at | 8.75E-03 | 5.18E-04 | -3.71764 | -0.48605 | -1.09303 | CELF2 |
| 219247_s_at | 2.60E-04 | 5.14E-06 | -5.12108 | 3.891034 | -1.09227 | ZDHHC14 |
| 205752_s_at | 2.69E-02 | 2.41E-03 | -3.20122 | -1.91328 | -1.09212 | GSTM5 |
| 202792_s_at | 3.16E-04 | 6.59E-06 | -5.04879 | 3.653078 | -1.09199 | PPP6R2 |
| 235561_at | 1.38E-02 | 9.55E-04 | -3.51666 | -1.05651 | -1.0916 | TXNL1 |
| 220120_s_at | 1.31E-01 | 2.79E-02 | -2.26555 | -4.11827 | -1.09122 | EPB41L4A |
| 225959_s_at | 1.59E-03 | 5.45E-05 | -4.422 | 1.63826 | -1.09105 | ZNRF1 |
| 209786_at | 1.05E-04 | 1.62E-06 | -5.45261 | 4.993467 | -1.09014 | HMGN4 |
| 223218_s_at | 8.79E-02 | 1.39E-02 | -2.5531 | -3.50054 | -1.09004 | NFKBIZ |
| 201368_at | 3.50E-04 | 7.60E-06 | -5.00685 | 3.515504 | -1.08981 | ZFP36L2 |
| 203567_s_at | 1.03E-02 | 6.48E-04 | -3.64453 | -0.69564 | -1.08852 | TRIM38 |
| 230475_at | 9.42E-06 | 7.20E-08 | -6.33511 | 7.988532 | -1.08779 | C15orf59 |
| 228046_at | 7.86E-04 | 2.17E-05 | -4.69882 | 2.516394 | -1.08765 | ZNF827 |
| 212203_x_at | 3.68E-03 | 1.62E-04 | -4.08669 | 0.604988 | -1.08755 | IFITM3 |
| 206033_s_at | 1.22E-02 | 8.18E-04 | -3.56815 | -0.91207 | -1.08666 | DSC3 |
| 1554717_a_at | 3.78E-03 | 1.68E-04 | -4.07635 | 0.573714 | -1.0858 | PDE4D |
| 215194_at | 2.24E-02 | 1.87E-03 | -3.28939 | -1.67885 | -1.08567 | PRKCA |
| 232704_s_at | 6.83E-03 | 3.71E-04 | -3.82589 | -0.17157 | -1.0839 | LRRFIP2 |
| 214696_at | 8.91E-02 | 1.41E-02 | -2.54555 | -3.51748 | -1.08383 | MIR22 |
| 200879_s_at | 2.39E-02 | 2.04E-03 | -3.25879 | -1.76068 | -1.08357 | EPAS1 |
| 215543_s_at | 8.11E-04 | 2.26E-05 | -4.68572 | 2.474391 | -1.08198 | LARGE |
| 209363_s_at | 3.02E-04 | 6.23E-06 | -5.06509 | 3.706648 | -1.08172 | LOC101928625 |
| 226948_at | 9.14E-04 | 2.64E-05 | -4.64005 | 2.328277 | -1.08157 | RHBDD1 |
| 235761_at | 9.37E-02 | 1.53E-02 | -2.51377 | -3.5884 | -1.08149 | CTA-445C9.15 |
| 1556204_a_at | 3.23E-03 | 1.36E-04 | -4.14095 | 0.769679 | -1.08135 | ZNF814 |
| 204793_at | 2.60E-02 | 2.29E-03 | -3.21896 | -1.86645 | -1.0809 | GPRASP1 |
| 205421_at | 1.03E-01 | 1.81E-02 | -2.44509 | -3.7393 | -1.08084 | SLC22A3 |
| 213800_at | 1.19E-02 | 7.90E-04 | -3.57949 | -0.88012 | -1.07981 | CFH |
| 205569_at | 2.61E-03 | 1.03E-04 | -4.22918 | 1.039616 | -1.07912 | LAMP3 |
| 210829_s_at | 9.28E-04 | 2.69E-05 | -4.63469 | 2.311156 | -1.07901 | SSBP2 |
| 230158_at | 8.94E-02 | 1.42E-02 | -2.54262 | -3.52405 | -1.07826 | DPY19L2 |
| 235742_at | 5.08E-03 | 2.47E-04 | -3.95488 | 0.209301 | -1.07776 | RHOC |
| 225613_at | 4.72E-03 | 2.26E-04 | -3.98364 | 0.295088 | -1.07771 | MAST4 |
| 1560074_at | 3.39E-03 | 1.45E-04 | -4.12102 | 0.709057 | -1.07751 | PRKCA |
| 241873_at | 9.39E-03 | 5.72E-04 | -3.68573 | -0.57781 | -1.07698 | RP11-710C12.1 |
| 228584_at | 4.92E-03 | 2.37E-04 | -3.96781 | 0.247815 | -1.07671 | SGCB |
| 222716_s_at | 7.97E-04 | 2.22E-05 | -4.69206 | 2.494688 | -1.07669 | SNX24 |
| 236359_at | 1.45E-02 | 1.03E-03 | -3.49239 | -1.12415 | -1.07665 | SCN4B |
| 219550_at | 1.37E-03 | 4.49E-05 | -4.4809 | 1.823353 | -1.07613 | ROBO3 |
| 204311_at | 2.57E-03 | 1.01E-04 | -4.23495 | 1.057371 | -1.07559 | ATP1B2 |
| 206765_at | 9.53E-02 | 1.58E-02 | -2.50103 | -3.61663 | -1.07463 | KCNJ2 |
| 200839_s_at | 1.10E-05 | 8.84E-08 | -6.27715 | 7.790218 | -1.07421 | CTSB |
| 227850_x_at | 1.27E-01 | 2.65E-02 | -2.28881 | -4.07047 | -1.07359 | CDC42EP5 |
| 202498_s_at | 1.02E-01 | 1.77E-02 | -2.45458 | -3.71865 | -1.07318 | SLC2A3 |
| 244111_at | 1.46E-05 | 1.25E-07 | -6.18039 | 7.459418 | -1.07312 | KRT222 |
| 224517_at | 1.29E-02 | 8.74E-04 | -3.54587 | -0.97472 | -1.07311 | POLR2J4 |
| 227198_at | 1.52E-01 | 3.67E-02 | -2.1485 | -4.35276 | -1.0725 | AFF3 |
| 200838_at | 2.90E-04 | 5.90E-06 | -5.08097 | 3.758897 | -1.07098 | CTSB |
| 210658_s_at | 2.65E-08 | 3.97E-11 | -8.4558 | 15.20478 | -1.07091 | GGA2 |
| 217173_s_at | 1.09E-01 | 2.01E-02 | -2.4039 | -3.82826 | -1.06923 | LDLR |
| 220835_s_at | 5.76E-05 | 7.37E-07 | -5.678 | 5.751713 | -1.06893 | ZNF407 |
| 1555281_x_at | 1.32E-04 | 2.17E-06 | -5.37009 | 4.717492 | -1.0689 | ARMC8 |
| 223842_s_at | 1.45E-02 | 1.03E-03 | -3.49191 | -1.12548 | -1.06789 | SCARA3 |
| 238615_at | 1.27E-03 | 4.02E-05 | -4.51399 | 1.927758 | -1.06762 | ERLIN2 |
| 215391_at | 2.46E-03 | 9.52E-05 | -4.25209 | 1.110131 | -1.0676 | MAP1A |
| 230068_s_at | 9.89E-03 | 6.11E-04 | -3.66413 | -0.63967 | -1.06708 | PEG3-AS1 |
| 203706_s_at | 1.54E-02 | 1.11E-03 | -3.46507 | -1.19996 | -1.06653 | FZD7 |
| 240715_at | 6.96E-02 | 9.54E-03 | -2.69839 | -3.16709 | -1.06622 | TBX5 |
| 207978_s_at | 1.58E-01 | 3.95E-02 | -2.11557 | -4.41689 | -1.06587 | NR4A3 |
| 207698_at | 3.22E-04 | 6.77E-06 | -5.0409 | 3.62719 | -1.06559 | C6orf123 |
| 222885_at | 6.71E-02 | 9.05E-03 | -2.71878 | -3.11919 | -1.06527 | EMCN |
| 230986_at | 2.62E-05 | 2.64E-07 | -5.96887 | 6.738079 | -1.06527 | KLF8 |
| 213547_at | 8.53E-03 | 5.01E-04 | -3.72879 | -0.45387 | -1.06526 | CAND2 |
| 239688_at | 3.04E-02 | 2.85E-03 | -3.14212 | -2.06813 | -1.06501 | SMC1A |
| 221261_x_at | 1.59E-01 | 3.98E-02 | -2.11185 | -4.42408 | -1.0649 | MAGED4 |
| 225262_at | 2.11E-02 | 1.71E-03 | -3.31908 | -1.599 | -1.06402 | FOSL2 |
| 236513_at | 2.64E-02 | 2.33E-03 | -3.21206 | -1.88469 | -1.06389 | PRELID2 |
| 201865_x_at | 1.35E-04 | 2.23E-06 | -5.36235 | 4.691659 | -1.06246 | NR3C1 |
| 227550_at | 1.25E-01 | 2.56E-02 | -2.30302 | -4.04108 | -1.06101 | GFRA1 |
| 208146_s_at | 6.74E-03 | 3.63E-04 | -3.83204 | -0.15357 | -1.06092 | CPVL |
| 230891_at | 1.35E-02 | 9.30E-04 | -3.52525 | -1.03251 | -1.06062 | TUBE1 |
| 1561705_at | 1.40E-04 | 2.33E-06 | -5.349 | 4.647085 | -1.06028 | BC037861 |
| 213372_at | 8.05E-03 | 4.64E-04 | -3.75359 | -0.38214 | -1.05949 | PAQR3 |
| 241627_x_at | 2.86E-03 | 1.16E-04 | -4.19068 | 0.921512 | -1.05909 | ARHGEF40 |
| 1565898_at | 7.50E-02 | 1.08E-02 | -2.65236 | -3.27422 | -1.05852 | METTL15 |
| 228080_at | 5.60E-02 | 6.82E-03 | -2.82521 | -2.865 | -1.05823 | LAYN |
| 212099_at | 2.19E-03 | 8.26E-05 | -4.29543 | 1.243999 | -1.05823 | RHOB |
| 217728_at | 2.72E-03 | 1.08E-04 | -4.21268 | 0.988953 | -1.05725 | S100A6 |
| 210912_x_at | 4.23E-02 | 4.56E-03 | -2.97349 | -2.49938 | -1.05681 | GSTM4 |
| 210718_s_at | 6.07E-02 | 7.73E-03 | -2.77852 | -2.97738 | -1.05681 | ARL17A |
| 212665_at | 7.66E-02 | 1.11E-02 | -2.64 | -3.30274 | -1.05678 | TIPARP |
| 226370_at | 5.27E-03 | 2.60E-04 | -3.9384 | 0.160256 | -1.05637 | KLHL15 |
| 235317_at | 5.87E-05 | 7.56E-07 | -5.6707 | 5.727079 | -1.05579 | LOC284454 |
| 215719_x_at | 4.00E-02 | 4.20E-03 | -3.00319 | -2.42459 | -1.05571 | FAS |
| 242465_at | 6.48E-02 | 8.54E-03 | -2.74069 | -3.06744 | -1.05517 | LOC100505592 |
| 242979_at | 3.04E-04 | 6.28E-06 | -5.0627 | 3.698799 | -1.0551 | IRS1 |
| 1555749_at | 1.93E-02 | 1.51E-03 | -3.36207 | -1.48258 | -1.05498 | SF1 |
| 217642_at | 4.28E-04 | 9.80E-06 | -4.9328 | 3.273434 | -1.05485 | RNF40 |
| 208735_s_at | 1.00E-03 | 2.98E-05 | -4.60399 | 2.213277 | -1.05477 | CTDSP2 |
| 207693_at | 1.73E-02 | 1.30E-03 | -3.41321 | -1.3429 | -1.05356 | CACNB4 |
| 238409_x_at | 1.01E-01 | 1.75E-02 | -2.46047 | -3.70579 | -1.05325 | OXR1 |
| 224480_s_at | 7.51E-02 | 1.08E-02 | -2.65108 | -3.27717 | -1.05258 | AGPAT9 |
| 1553956_at | 6.90E-03 | 3.76E-04 | -3.8214 | -0.1847 | -1.05233 | TMEM237 |
| 228128_x_at | 2.82E-02 | 2.58E-03 | -3.17667 | -1.97785 | -1.05149 | PAPPA |
| 229518_at | 3.44E-03 | 1.48E-04 | -4.11564 | 0.69273 | -1.05147 | FAM46B |
| 231211_s_at | 1.70E-01 | 4.59E-02 | -2.04814 | -4.54563 | -1.05136 | YIF1B |
| 232521_at | 5.75E-03 | 2.93E-04 | -3.90111 | 0.049744 | -1.05132 | PCSK7 |
| 242062_at | 4.80E-03 | 2.30E-04 | -3.97746 | 0.276618 | -1.05088 | SAMD8 |
| 45297_at | 2.96E-04 | 6.04E-06 | -5.0738 | 3.735301 | -1.04988 | EHD2 |
| 215415_s_at | 1.08E-02 | 6.93E-04 | -3.62276 | -0.75759 | -1.0498 | LYST |
| 237058_x_at | 5.76E-03 | 2.94E-04 | -3.90031 | 0.047374 | -1.0496 | SLC6A13 |
| 223392_s_at | 5.16E-05 | 6.32E-07 | -5.72178 | 5.899695 | -1.04959 | TSHZ3 |
| 204028_s_at | 6.59E-06 | 4.71E-08 | -6.45454 | 8.397562 | -1.04891 | RABGAP1 |
| 202274_at | 2.55E-02 | 2.22E-03 | -3.22904 | -1.83976 | -1.04874 | ACTG2 |
| 1559513_a_at | 5.26E-05 | 6.49E-07 | -5.71418 | 5.873993 | -1.04843 | FANCC |
| 213590_at | 4.74E-06 | 3.03E-08 | -6.5783 | 8.821817 | -1.04695 | SLC16A5 |
| 205961_s_at | 9.95E-04 | 2.95E-05 | -4.60687 | 2.222436 | -1.04544 | PSIP1 |
| 242985_x_at | 1.15E-03 | 3.55E-05 | -4.55172 | 2.047194 | -1.04513 | RNF180 |
| 212501_at | 2.51E-03 | 9.76E-05 | -4.24452 | 1.086811 | -1.0445 | CEBPB |
| 240050_s_at | 3.62E-02 | 3.65E-03 | -3.05395 | -2.29561 | -1.04351 | PPM1K |
| 202748_at | 1.66E-03 | 5.74E-05 | -4.40625 | 1.588935 | -1.04303 | GBP2 |
| 209723_at | 3.59E-03 | 1.57E-04 | -4.09811 | 0.639562 | -1.04275 | SERPINB9 |
| 208892_s_at | 3.46E-02 | 3.43E-03 | -3.07665 | -2.23746 | -1.04263 | DUSP6 |
| 206675_s_at | 6.34E-05 | 8.45E-07 | -5.63932 | 5.621154 | -1.04231 | SKIL |
| 234967_at | 7.86E-02 | 1.16E-02 | -2.624 | -3.33954 | -1.0422 | IL6ST |
| 218364_at | 3.11E-04 | 6.47E-06 | -5.05393 | 3.669976 | -1.04215 | LRRFIP2 |
| 223093_at | 5.31E-02 | 6.33E-03 | -2.85349 | -2.79628 | -1.04186 | ANKH |
| 220051_at | 7.45E-03 | 4.16E-04 | -3.78856 | -0.28056 | -1.04182 | PRSS21 |
| 207000_s_at | 7.48E-03 | 4.19E-04 | -3.78606 | -0.28783 | -1.04168 | PPP3CC |
| 215195_at | 5.11E-03 | 2.49E-04 | -3.95196 | 0.200594 | -1.0416 | PRKCA |
| 235505_s_at | 1.72E-02 | 1.29E-03 | -3.41576 | -1.33592 | -1.04103 | LRPAP1 |
| 244779_at | 2.65E-03 | 1.05E-04 | -4.22322 | 1.021295 | -1.04072 | ZDHHC2 |
| 201060_x_at | 1.07E-05 | 8.45E-08 | -6.29005 | 7.834338 | -1.04044 | STOM |
| 229288_at | 4.69E-02 | 5.31E-03 | -2.91797 | -2.63782 | -1.04004 | EPHA7 |
| 208498_s_at | 5.69E-02 | 6.99E-03 | -2.81622 | -2.88675 | -1.03962 | ACTG1P4 |
| 244401_at | 8.06E-03 | 4.64E-04 | -3.75324 | -0.38316 | -1.03871 | LCA5 |
| 1556672_a_at | 6.38E-02 | 8.34E-03 | -2.74952 | -3.0465 | -1.03801 | RBM6 |
| 1560109_s_at | 1.57E-01 | 3.89E-02 | -2.12204 | -4.40435 | -1.03725 | AK055458 |
| 239725_at | 8.96E-05 | 1.33E-06 | -5.51029 | 5.186906 | -1.03691 | PGAP1 |
| 205017_s_at | 7.54E-03 | 4.24E-04 | -3.78278 | -0.2974 | -1.03665 | MBNL2 |
| 216856_s_at | 2.04E-02 | 1.62E-03 | -3.33723 | -1.54997 | -1.03648 | DLEU2L |
| 221870_at | 5.14E-03 | 2.52E-04 | -3.9492 | 0.192396 | -1.03646 | EHD2 |
| 37590_g_at | 3.26E-03 | 1.38E-04 | -4.13747 | 0.759085 | -1.03508 | AK055981 |
| 203685_at | 4.91E-02 | 5.67E-03 | -2.89383 | -2.69742 | -1.03481 | BCL2 |
| 204036_at | 8.05E-03 | 4.63E-04 | -3.75404 | -0.38085 | -1.03478 | LPAR1 |
| 242800_at | 3.23E-03 | 1.36E-04 | -4.1415 | 0.771343 | -1.03421 | NHS |
| 201678_s_at | 1.07E-03 | 3.23E-05 | -4.58003 | 2.137045 | -1.03296 | HMCES |
| 240703_s_at | 6.32E-04 | 1.62E-05 | -4.7856 | 2.795691 | -1.03277 | HERC1 |
| 225363_at | 1.24E-02 | 8.34E-04 | -3.56136 | -0.93119 | -1.03238 | PTEN |
| 241812_at | 1.12E-03 | 3.44E-05 | -4.56053 | 2.07514 | -1.03227 | SPATS2L |
| 1568647_at | 9.81E-02 | 1.66E-02 | -2.48074 | -3.66138 | -1.0322 | LOC100505851 |
| 226425_at | 2.90E-03 | 1.19E-04 | -4.18404 | 0.901194 | -1.0318 | CLIP4 |
| 235570_at | 7.23E-03 | 4.01E-04 | -3.80068 | -0.24526 | -1.03152 | RBMS3 |
| 228667_at | 8.29E-03 | 4.83E-04 | -3.74023 | -0.42081 | -1.03147 | AGPAT4 |
| 217630_at | 4.88E-02 | 5.63E-03 | -2.89643 | -2.69102 | -1.03011 | ANGEL2 |
| 211367_s_at | 1.06E-02 | 6.74E-04 | -3.63191 | -0.73157 | -1.02986 | CASP1 |
| 222124_at | 4.55E-04 | 1.06E-05 | -4.90972 | 3.198229 | -1.02964 | HIF3A |
| 213194_at | 3.04E-02 | 2.85E-03 | -3.1422 | -2.06792 | -1.02958 | ROBO1 |
| 53991_at | 1.36E-03 | 4.42E-05 | -4.48531 | 1.837247 | -1.02824 | DENND2A |
| 218487_at | 1.26E-04 | 2.04E-06 | -5.38725 | 4.774793 | -1.02819 | ALAD |
| 224367_at | 6.80E-03 | 3.69E-04 | -3.82739 | -0.16719 | -1.02815 | BEX2 |
| 222722_at | 4.66E-02 | 5.28E-03 | -2.92055 | -2.63141 | -1.02801 | OGN |
| 1552478_a_at | 1.87E-05 | 1.69E-07 | -6.09451 | 7.166216 | -1.0271 | IRF6 |
| 1553590_at | 1.66E-02 | 1.23E-03 | -3.43088 | -1.29435 | -1.02665 | FAM27E2 |
| 206540_at | 1.18E-02 | 7.78E-04 | -3.58466 | -0.86552 | -1.02639 | GLB1L |
| 1555630_a_at | 2.53E-03 | 9.84E-05 | -4.24196 | 1.078933 | -1.02613 | NARR |
| 1563595_at | 3.20E-02 | 3.07E-03 | -3.11599 | -2.13601 | -1.02593 | SRGAP3 |
| 1556739_at | 3.53E-02 | 3.53E-03 | -3.06613 | -2.26445 | -1.02573 | GOLGA8I |
| 217226_s_at | 1.30E-03 | 4.16E-05 | -4.50396 | 1.896083 | -1.02544 | SFXN3 |
| 225975_at | 2.22E-02 | 1.85E-03 | -3.29316 | -1.66874 | -1.02538 | PCDH18 |
| 207275_s_at | 4.32E-02 | 4.72E-03 | -2.96159 | -2.52921 | -1.02531 | ACSL1 |
| 205067_at | 5.43E-02 | 6.54E-03 | -2.84131 | -2.82594 | -1.02499 | IL1B |
| 232288_at | 3.79E-03 | 1.69E-04 | -4.07503 | 0.569715 | -1.02483 | LOC102724985 |
| 213371_at | 3.22E-02 | 3.10E-03 | -3.11256 | -2.14487 | -1.02465 | LDB3 |
| 218729_at | 3.15E-02 | 3.00E-03 | -3.12359 | -2.1163 | -1.02435 | LXN |
| 1566968_at | 2.12E-02 | 1.72E-03 | -3.3167 | -1.60542 | -1.02409 | SPRY4-IT1 |
| 203344_s_at | 3.55E-02 | 3.55E-03 | -3.06355 | -2.27106 | -1.02373 | RBBP8 |
| 214791_at | 6.91E-04 | 1.83E-05 | -4.74914 | 2.678141 | -1.02347 | SP140L |
| 233766_at | 6.41E-02 | 8.39E-03 | -2.74744 | -3.05143 | -1.02257 | SOBP |
| 223394_at | 7.29E-02 | 1.03E-02 | -2.66908 | -3.23546 | -1.02251 | SERTAD1 |
| 239252_at | 3.02E-02 | 2.82E-03 | -3.14511 | -2.06035 | -1.0224 | COX7B |
| 1559265_at | 2.77E-05 | 2.86E-07 | -5.94666 | 6.662514 | -1.02238 | SKIDA1 |
| 1555199_at | 1.62E-04 | 2.81E-06 | -5.29582 | 4.469937 | -1.02232 | GOSR1 |
| 209569_x_at | 1.85E-02 | 1.43E-03 | -3.38128 | -1.43026 | -1.02187 | NSG1 |
| 240637_at | 4.98E-03 | 2.41E-04 | -3.96332 | 0.234437 | -1.02169 | WDR41 |
| 238725_at | 1.66E-01 | 4.37E-02 | -2.07096 | -4.50246 | -1.02132 | IRF1 |
| 242918_at | 7.04E-04 | 1.87E-05 | -4.74223 | 2.655873 | -1.02024 | NASP |
| 1558693_s_at | 4.25E-02 | 4.59E-03 | -2.97097 | -2.50571 | -1.02006 | C1orf85 |
| 1555372_at | 5.31E-02 | 6.34E-03 | -2.85278 | -2.79802 | -1.01942 | BCL2L11 |
| 1563321_s_at | 3.31E-02 | 3.23E-03 | -3.09715 | -2.1847 | -1.01831 | MLLT10 |
| 202794_at | 6.58E-02 | 8.78E-03 | -2.72995 | -3.09285 | -1.01802 | INPP1 |
| 228959_at | 5.79E-04 | 1.44E-05 | -4.81981 | 2.906316 | -1.01769 | PDK3 |
| 209362_at | 8.66E-04 | 2.46E-05 | -4.6612 | 2.39586 | -1.01681 | LOC101928625 |
| 208790_s_at | 1.61E-04 | 2.78E-06 | -5.29864 | 4.479328 | -1.01643 | PTRF |
| 223044_at | 1.87E-02 | 1.46E-03 | -3.37455 | -1.44862 | -1.01639 | SLC40A1 |
| 226423_at | 2.18E-03 | 8.21E-05 | -4.29729 | 1.249748 | -1.01527 | PAQR8 |
| 212859_x_at | 1.64E-01 | 4.28E-02 | -2.07991 | -4.48542 | -1.01452 | MT1E |
| 227113_at | 2.68E-02 | 2.40E-03 | -3.20216 | -1.91081 | -1.01373 | ADHFE1 |
| 207227_x_at | 1.20E-01 | 2.35E-02 | -2.33848 | -3.96708 | -1.01344 | RFPL2 |
| 214013_s_at | 3.22E-04 | 6.78E-06 | -5.04042 | 3.625606 | -1.01297 | TBC1D1 |
| 211671_s_at | 9.82E-04 | 2.89E-05 | -4.61269 | 2.24097 | -1.01279 | NR3C1 |
| 204433_s_at | 3.66E-03 | 1.61E-04 | -4.08911 | 0.612308 | -1.01255 | SPATA2 |
| 213055_at | 8.44E-04 | 2.38E-05 | -4.67118 | 2.427814 | -1.01247 | CD47 |
| 214033_at | 5.23E-03 | 2.58E-04 | -3.94139 | 0.169165 | -1.01153 | ABCC6 |
| 204924_at | 9.60E-02 | 1.60E-02 | -2.4964 | -3.62686 | -1.01002 | TLR2 |
| 217489_s_at | 3.46E-02 | 3.42E-03 | -3.07686 | -2.23691 | -1.00941 | IL6R |
| 206816_s_at | 1.58E-03 | 5.40E-05 | -4.42506 | 1.647852 | -1.00898 | SPAG8 |
| 229994_at | 4.76E-04 | 1.12E-05 | -4.89369 | 3.146052 | -1.00843 | NFIA |
| 232230_at | 1.19E-01 | 2.32E-02 | -2.3434 | -3.95675 | -1.00787 | LINC00263 |
| 212926_at | 3.68E-03 | 1.63E-04 | -4.0865 | 0.604414 | -1.0078 | SMC5 |
| 206465_at | 3.22E-03 | 1.36E-04 | -4.14259 | 0.774669 | -1.00749 | ACSBG1 |
| 1555444_a_at | 9.61E-02 | 1.60E-02 | -2.49594 | -3.62788 | -1.00746 | PPP1R12B |
| 233599_at | 1.30E-03 | 4.16E-05 | -4.50372 | 1.895336 | -1.00704 | LOC728061 |
| 213659_at | 6.95E-03 | 3.80E-04 | -3.81765 | -0.19569 | -1.00692 | ZNF75D |
| 238613_at | 2.73E-02 | 2.46E-03 | -3.19375 | -1.93297 | -1.00665 | ZAK |
| 228555_at | 2.66E-02 | 2.37E-03 | -3.20689 | -1.89835 | -1.00619 | CAMK2D |
| 235368_at | 1.85E-02 | 1.43E-03 | -3.38029 | -1.43298 | -1.00617 | ADAMTS5 |
| 209368_at | 6.16E-02 | 7.91E-03 | -2.76994 | -2.99788 | -1.00576 | EPHX2 |
| 227314_at | 2.74E-02 | 2.47E-03 | -3.19215 | -1.93717 | -1.00563 | ITGA2 |
| 208868_s_at | 1.59E-03 | 5.46E-05 | -4.42158 | 1.636944 | -1.00549 | GABARAPL1 |
| 203603_s_at | 1.74E-03 | 6.12E-05 | -4.38707 | 1.528985 | -1.00545 | ZEB2 |
| 202053_s_at | 1.46E-04 | 2.48E-06 | -5.33185 | 4.589931 | -1.00515 | ALDH3A2 |
| 210635_s_at | 7.51E-02 | 1.08E-02 | -2.6512 | -3.2769 | -1.00392 | KLHL20 |
| 202499_s_at | 1.64E-01 | 4.27E-02 | -2.08084 | -4.48364 | -1.00366 | SLC2A3 |
| 228723_at | 1.22E-02 | 8.18E-04 | -3.56797 | -0.91258 | -1.0031 | NPTN-IT1 |
| 229128_s_at | 5.36E-03 | 2.67E-04 | -3.93052 | 0.136866 | -1.00259 | ANP32E |
| 206356_s_at | 2.18E-04 | 4.06E-06 | -5.18913 | 4.115846 | -1.00246 | GNAL |
| 242123_at | 3.16E-04 | 6.61E-06 | -5.04784 | 3.649969 | -1.00207 | PAQR7 |
| 242137_at | 4.46E-03 | 2.09E-04 | -4.007 | 0.364999 | -1.00194 | RBMS3 |
| 237504_at | 7.72E-03 | 4.36E-04 | -3.77343 | -0.32457 | -1.00181 | INTS10 |
| 228006_at | 3.27E-03 | 1.39E-04 | -4.13605 | 0.75477 | -1.00174 | PTEN |
| 218880_at | 6.72E-02 | 9.07E-03 | -2.71782 | -3.12146 | -1.00168 | FOSL2 |
| 208965_s_at | 1.01E-02 | 6.32E-04 | -3.65265 | -0.67247 | -1.0012 | IFI16 |
| 226996_at | 3.26E-03 | 1.38E-04 | 4.137108 | 0.757984 | 1.000487 | LCLAT1 |
| 230093_at | 1.20E-01 | 2.36E-02 | 2.336308 | -3.97165 | 1.000541 | RSPH1 |
| 244334_at | 1.71E-01 | 4.61E-02 | 2.046638 | -4.54847 | 1.000872 | TRAM1L1 |
| 229363_at | 1.11E-01 | 2.06E-02 | 2.392775 | -3.85208 | 1.001089 | LINC00920 |
| 213610_s_at | 6.68E-04 | 1.74E-05 | 4.763272 | 2.72367 | 1.001229 | KLHL23 |
| 223031_s_at | 5.11E-04 | 1.23E-05 | 4.865137 | 3.053251 | 1.001524 | TRAF7 |
| 218676_s_at | 3.95E-02 | 4.14E-03 | 3.008499 | -2.41117 | 1.00177 | PCTP |
| 222993_at | 2.12E-03 | 7.92E-05 | 4.308343 | 1.284004 | 1.002251 | MRPL37 |
| 240592_at | 3.87E-03 | 1.73E-04 | 4.066191 | 0.543034 | 1.002761 | LCORL |
| 218906_x_at | 2.49E-04 | 4.85E-06 | 5.137938 | 3.946644 | 1.003685 | KLC2 |
| 213385_at | 1.62E-01 | 4.15E-02 | 2.093732 | -4.45897 | 1.005248 | CHN2 |
| 226452_at | 1.08E-03 | 3.26E-05 | 4.577024 | 2.1275 | 1.005453 | PDK1 |
| 220187_at | 4.44E-02 | 4.90E-03 | 2.947656 | -2.56401 | 1.005757 | STEAP4 |
| 221782_at | 3.20E-04 | 6.70E-06 | 5.043659 | 3.636242 | 1.006172 | DNAJC10 |
| 218670_at | 1.19E-03 | 3.71E-05 | 4.537823 | 2.003159 | 1.006277 | PUS1 |
| 225867_at | 1.00E-04 | 1.53E-06 | 5.469052 | 5.048561 | 1.006312 | VASN |
| 213871_s_at | 5.48E-04 | 1.35E-05 | 4.839734 | 2.970842 | 1.006915 | DNPH1 |
| 224857_s_at | 2.57E-04 | 5.04E-06 | 5.126424 | 3.908647 | 1.007984 | POLR1D |
| 242274_x_at | 1.22E-02 | 8.15E-04 | 3.569203 | -0.90911 | 1.009412 | SLC25A42 |
| 229050_s_at | 9.70E-03 | 5.97E-04 | 3.671569 | -0.61839 | 1.009597 | SNHG7 |
| 228115_at | 4.31E-02 | 4.69E-03 | 2.963178 | -2.52523 | 1.010185 | GAREM |
| 215511_at | 9.30E-03 | 5.63E-04 | 3.690699 | -0.56355 | 1.010439 | TCF20 |
| 228832_at | 9.14E-03 | 5.51E-04 | 3.697859 | -0.54298 | 1.010532 | FLJ20021 |
| 219664_s_at | 2.00E-04 | 3.66E-06 | 5.218868 | 4.214364 | 1.010852 | DECR2 |
| 235145_at | 1.44E-03 | 4.76E-05 | 4.462928 | 1.76677 | 1.011438 | ZBTB7B |
| 206553_at | 5.04E-02 | 5.88E-03 | 2.880846 | -2.72935 | 1.011834 | OAS2 |
| 228124_at | 1.25E-01 | 2.56E-02 | 2.301911 | -4.04337 | 1.013609 | ABHD12 |
| 205938_at | 8.46E-02 | 1.30E-02 | 2.57742 | -3.44568 | 1.013945 | PPM1E |
| 202812_at | 1.73E-02 | 1.30E-03 | 3.412119 | -1.3459 | 1.014387 | TARBP1 |
| 212511_at | 1.92E-03 | 6.94E-05 | 4.34861 | 1.409064 | 1.014727 | PICALM |
| 200085_s_at | 1.83E-04 | 3.28E-06 | 5.250347 | 4.318798 | 1.016068 | TCEB2 |
| 203362_s_at | 7.81E-02 | 1.14E-02 | 2.628467 | -3.32928 | 1.016487 | MAD2L1 |
| 241604_at | 5.13E-02 | 6.02E-03 | 2.871637 | -2.75193 | 1.016607 | ATP11A |
| 236738_at | 7.04E-03 | 3.87E-04 | 3.811653 | -0.21321 | 1.016914 | C3orf80 |
| 218756_s_at | 3.01E-03 | 1.25E-04 | 4.168785 | 0.85456 | 1.017473 | DHRS11 |
| 201839_s_at | 2.53E-06 | 1.34E-08 | 6.807106 | 9.606568 | 1.018234 | EPCAM |
| 230851_x_at | 1.59E-04 | 2.74E-06 | 5.302155 | 4.49102 | 1.018238 | C16orf13 |
| 205449_at | 4.20E-05 | 4.84E-07 | 5.797247 | 6.155176 | 1.018743 | SAC3D1 |
| 206482_at | 5.09E-02 | 5.96E-03 | 2.875414 | -2.74268 | 1.018879 | PTK6 |
| 238623_at | 2.47E-02 | 2.13E-03 | 3.244341 | -1.79914 | 1.019724 | RP3-428L16.2 |
| 218865_at | 1.07E-02 | 6.78E-04 | 3.629848 | -0.73744 | 1.020101 | 42795 |
| 223678_s_at | 8.00E-02 | 1.19E-02 | 2.611602 | -3.36792 | 1.020266 | SFTPA1 |
| 205566_at | 4.88E-02 | 5.63E-03 | 2.896835 | -2.69002 | 1.020314 | ABHD2 |
| 216958_s_at | 2.09E-03 | 7.76E-05 | 4.314871 | 1.304243 | 1.021592 | IVD |
| 235497_at | 9.82E-02 | 1.66E-02 | 2.479816 | -3.6634 | 1.022879 | LINC01128 |
| 211214_s_at | 9.36E-02 | 1.53E-02 | 2.51438 | -3.58704 | 1.023329 | DAPK1 |
| 231815_at | 1.22E-03 | 3.84E-05 | 4.527841 | 1.971563 | 1.025171 | PHF12 |
| 205225_at | 1.38E-01 | 3.08E-02 | 2.223583 | -4.2035 | 1.027189 | ESR1 |
| 235746_s_at | 9.71E-02 | 1.63E-02 | 2.487699 | -3.64606 | 1.028118 | PLA2R1 |
| 227804_at | 1.23E-02 | 8.25E-04 | 3.565302 | -0.9201 | 1.028749 | TLCD1 |
| 226063_at | 7.79E-05 | 1.10E-06 | 5.565228 | 5.371568 | 1.029008 | VAV2 |
| 227580_s_at | 1.15E-02 | 7.54E-04 | 3.5947 | -0.83714 | 1.031521 | TECPR1 |
| 229488_at | 2.59E-03 | 1.02E-04 | 4.232147 | 1.048752 | 1.033181 | OTUD7B |
| 230639_at | 1.08E-02 | 6.88E-04 | 3.62508 | -0.751 | 1.033428 | ICA1 |
| 220432_s_at | 3.67E-02 | 3.71E-03 | 3.047973 | -2.31087 | 1.033688 | CYP39A1 |
| 218512_at | 4.51E-04 | 1.05E-05 | 4.913343 | 3.210023 | 1.033788 | WDR12 |
| 207391_s_at | 5.12E-03 | 2.50E-04 | 3.951291 | 0.198608 | 1.034294 | PIP5K1A |
| 209785_s_at | 1.10E-01 | 2.03E-02 | 2.398771 | -3.83925 | 1.034621 | PLA2G4C |
| 223895_s_at | 6.66E-03 | 3.58E-04 | 3.837347 | -0.13801 | 1.035163 | EPN3 |
| 225987_at | 2.15E-02 | 1.76E-03 | 3.310543 | -1.622 | 1.03532 | STEAP4 |
| 219178_at | 6.34E-05 | 8.44E-07 | 5.639626 | 5.622205 | 1.035325 | QTRTD1 |
| 224753_at | 2.59E-02 | 2.27E-03 | 3.221795 | -1.85894 | 1.035464 | CDCA5 |
| 212539_at | 1.62E-05 | 1.41E-07 | 6.145587 | 7.340551 | 1.036988 | CHD1L |
| 218647_s_at | 1.81E-03 | 6.45E-05 | 4.371062 | 1.47901 | 1.038951 | YRDC |
| 213059_at | 5.16E-02 | 6.09E-03 | 2.867852 | -2.7612 | 1.039134 | CREB3L1 |
| 221269_s_at | 1.42E-04 | 2.37E-06 | 5.344598 | 4.632423 | 1.039757 | SH3BGRL3 |
| 218888_s_at | 2.69E-02 | 2.41E-03 | 3.201034 | -1.91378 | 1.040069 | NETO2 |
| 225590_at | 5.47E-02 | 6.61E-03 | 2.837268 | -2.83576 | 1.040325 | SH3RF1 |
| 222883_at | 6.83E-05 | 9.27E-07 | 5.612974 | 5.532344 | 1.040967 | COA7 |
| 212344_at | 3.64E-02 | 3.68E-03 | 3.051469 | -2.30195 | 1.041247 | SULF1 |
| 206429_at | 5.79E-02 | 7.19E-03 | 2.805906 | -2.91163 | 1.041333 | F2RL1 |
| 227988_s_at | 6.64E-03 | 3.56E-04 | 3.838348 | -0.13508 | 1.041461 | VPS13A |
| 229899_s_at | 2.27E-02 | 1.90E-03 | 3.282663 | -1.69687 | 1.041761 | ZFAS1 |
| 212056_at | 1.23E-02 | 8.22E-04 | 3.566181 | -0.91762 | 1.042135 | GSE1 |
| 214776_x_at | 6.79E-04 | 1.78E-05 | 4.756454 | 2.701695 | 1.042246 | XYLB |
| 219133_at | 2.30E-02 | 1.93E-03 | 3.277191 | -1.71152 | 1.042483 | OXSM |
| 203926_x_at | 1.46E-05 | 1.24E-07 | 6.180955 | 7.461348 | 1.04259 | ATP5D |
| 238199_x_at | 2.79E-06 | 1.53E-08 | 6.769097 | 9.476198 | 1.045155 | COX3 |
| 226549_at | 3.56E-04 | 7.78E-06 | 5.000265 | 3.493936 | 1.045741 | SBK1 |
| 211823_s_at | 1.94E-02 | 1.53E-03 | 3.358725 | -1.49168 | 1.046729 | PXN |
| 213912_at | 3.19E-02 | 3.06E-03 | 3.117383 | -2.13239 | 1.047199 | TBC1D30 |
| 1554408_a_at | 1.08E-03 | 3.25E-05 | 4.577754 | 2.129821 | 1.04737 | TK1 |
| 204432_at | 1.70E-05 | 1.50E-07 | 6.128971 | 7.283821 | 1.047378 | SOX12 |
| 35147_at | 3.61E-02 | 3.64E-03 | 3.055445 | -2.29179 | 1.047632 | MCF2L |
| 202264_s_at | 1.14E-05 | 9.23E-08 | 6.265156 | 7.749179 | 1.047772 | TOMM40 |
| 212687_at | 1.31E-02 | 8.94E-04 | 3.538319 | -0.9959 | 1.047871 | LIMS1 |
| 235315_at | 7.91E-02 | 1.17E-02 | 2.619054 | -3.35087 | 1.047963 | TSC22D1 |
| 222480_at | 2.09E-02 | 1.68E-03 | 3.324752 | -1.58369 | 1.048343 | UBE2Q1 |
| 232914_s_at | 9.86E-02 | 1.68E-02 | 2.476365 | -3.67098 | 1.0486 | SYTL2 |
| 218491_s_at | 3.48E-02 | 3.46E-03 | 3.073256 | -2.24617 | 1.049653 | THYN1 |
| 204025_s_at | 3.94E-03 | 1.77E-04 | 4.058996 | 0.521323 | 1.050294 | PDCD2 |
| 242098_at | 8.27E-03 | 4.82E-04 | 3.740974 | -0.41867 | 1.050465 | KIAA1244 |
| 224874_at | 2.98E-02 | 2.78E-03 | 3.150703 | -2.04577 | 1.050517 | POLR1D |
| 203431_s_at | 6.49E-03 | 3.46E-04 | 3.848212 | -0.10613 | 1.050983 | ARHGAP32 |
| 210130_s_at | 1.14E-02 | 7.42E-04 | 3.600314 | -0.82125 | 1.05377 | TM7SF2 |
| 228003_at | 1.15E-03 | 3.53E-05 | 4.552884 | 2.05088 | 1.053808 | RAB30 |
| 202613_at | 8.26E-03 | 4.81E-04 | 3.741502 | -0.41714 | 1.053934 | CTPS1 |
| 1555039_a_at | 2.90E-03 | 1.18E-04 | 4.184883 | 0.903772 | 1.053937 | ABCC4 |
| 227045_at | 1.32E-02 | 9.03E-04 | 3.535318 | -1.00431 | 1.054662 | ZNF614 |
| 201614_s_at | 9.89E-03 | 6.10E-04 | 3.664532 | -0.63852 | 1.054839 | RUVBL1 |
| 235928_at | 8.93E-02 | 1.42E-02 | 2.543339 | -3.52243 | 1.054984 | ZNF503-AS2 |
| 1552834_at | 6.57E-02 | 8.76E-03 | 2.731037 | -3.09028 | 1.055941 | B3GNT6 |
| 209567_at | 1.44E-05 | 1.22E-07 | 6.187019 | 7.482067 | 1.056228 | RRS1 |
| 1554677_s_at | 7.54E-04 | 2.05E-05 | 4.715627 | 2.570342 | 1.056356 | CMTM4 |
| 225174_at | 9.58E-04 | 2.79E-05 | 4.623037 | 2.273969 | 1.057081 | DNAJC10 |
| 211715_s_at | 5.06E-04 | 1.22E-05 | 4.869354 | 3.066948 | 1.057316 | BDH1 |
| 236308_at | 1.46E-01 | 3.39E-02 | 2.18201 | -4.28666 | 1.057486 | VSTM2A |
| 203700_s_at | 1.08E-01 | 1.97E-02 | 2.411004 | -3.813 | 1.058102 | DIO2 |
| 222212_s_at | 9.06E-03 | 5.45E-04 | 3.70147 | -0.5326 | 1.060797 | CERS2 |
| 223296_at | 1.05E-02 | 6.60E-04 | 3.638675 | -0.71231 | 1.060851 | SLC25A33 |
| 1565756_a_at | 8.11E-02 | 1.22E-02 | 2.60321 | -3.38708 | 1.06169 | METAP1D |
| 39549_at | 1.07E-02 | 6.78E-04 | 3.629784 | -0.73762 | 1.061771 | NPAS2 |
| 212186_at | 3.66E-06 | 2.17E-08 | 6.672243 | 9.14399 | 1.062093 | ACACA |
| 228081_at | 2.84E-03 | 1.15E-04 | 4.194234 | 0.932397 | 1.062844 | CCNG2 |
| 228209_at | 4.64E-03 | 2.21E-04 | 3.989625 | 0.312976 | 1.063662 | LHX4-AS1 |
| 214193_s_at | 2.80E-04 | 5.61E-06 | 5.095674 | 3.807291 | 1.063892 | DIEXF |
| 235065_at | 5.25E-03 | 2.59E-04 | 3.939755 | 0.164298 | 1.063936 | GAREM |
| 223204_at | 1.49E-02 | 1.07E-03 | 3.479236 | -1.1607 | 1.064552 | FAM198B |
| 239670_at | 1.67E-04 | 2.94E-06 | 5.282266 | 4.424853 | 1.064984 | WNK2 |
| 201262_s_at | 1.15E-01 | 2.20E-02 | 2.365373 | -3.91039 | 1.065195 | BGN |
| 242462_at | 3.25E-04 | 6.90E-06 | 5.035418 | 3.609187 | 1.065374 | LINC00665 |
| 244749_at | 1.98E-02 | 1.56E-03 | 3.350369 | -1.51436 | 1.068471 | LOC101927204 |
| 212692_s_at | 5.17E-05 | 6.34E-07 | 5.720769 | 5.896263 | 1.069858 | LRBA |
| 217430_x_at | 1.60E-04 | 2.76E-06 | 5.300053 | 4.484023 | 1.070012 | COL1A1 |
| 213506_at | 9.27E-02 | 1.50E-02 | 2.520464 | -3.57351 | 1.070794 | F2RL1 |
| 204695_at | 2.63E-03 | 1.04E-04 | 4.226032 | 1.029953 | 1.071032 | CDC25A |
| 225097_at | 8.02E-03 | 4.61E-04 | 3.755545 | -0.37648 | 1.07143 | HIPK2 |
| 202138_x_at | 1.06E-05 | 8.39E-08 | 6.292102 | 7.841359 | 1.071522 | AIMP2 |
| 1552367_a_at | 1.56E-01 | 3.87E-02 | 2.125167 | -4.39828 | 1.071621 | SCIN |
| 204087_s_at | 3.52E-04 | 7.67E-06 | 5.004531 | 3.50791 | 1.073287 | SLC5A6 |
| 237745_at | 1.74E-02 | 1.31E-03 | 3.410221 | -1.35111 | 1.074212 | TSC22D1-AS1 |
| 212292_at | 9.56E-03 | 5.84E-04 | 3.678463 | -0.59864 | 1.074567 | SLC7A1 |
| 227698_s_at | 1.27E-03 | 4.04E-05 | 4.512762 | 1.923887 | 1.074654 | RAB40C |
| 231826_at | 3.15E-03 | 1.32E-04 | 4.150911 | 0.800027 | 1.075477 | RALGAPA2 |
| 231321_s_at | 9.28E-02 | 1.51E-02 | 2.519231 | -3.57625 | 1.075498 | ACER3 |
| 201764_at | 5.29E-03 | 2.62E-04 | 3.936707 | 0.155243 | 1.076225 | TMEM106C |
| 217979_at | 1.31E-04 | 2.14E-06 | 5.37343 | 4.728631 | 1.077304 | TSPAN13 |
| 219749_at | 2.75E-03 | 1.09E-04 | 4.209585 | 0.979451 | 1.077525 | SH2D4A |
| 228051_at | 8.44E-04 | 2.37E-05 | 4.671483 | 2.428772 | 1.077759 | KIAA1244 |
| 203397_s_at | 2.14E-02 | 1.75E-03 | 3.312306 | -1.61725 | 1.077771 | GALNT3 |
| 219926_at | 1.31E-01 | 2.79E-02 | 2.266569 | -4.11618 | 1.078262 | POPDC3 |
| 1557998_at | 7.61E-02 | 1.10E-02 | 2.64331 | -3.29511 | 1.078309 | NAALADL2 |
| 241603_at | 4.21E-02 | 4.53E-03 | 2.976481 | -2.49187 | 1.078592 | ATP11A |
| 237515_at | 1.21E-02 | 8.06E-04 | 3.573019 | -0.89836 | 1.078754 | TMEM56 |
| 213288_at | 4.07E-02 | 4.32E-03 | 2.993557 | -2.4489 | 1.079234 | MBOAT2 |
| 212556_at | 3.15E-05 | 3.39E-07 | 5.898681 | 6.499402 | 1.079806 | SCRIB |
| 215726_s_at | 1.93E-03 | 6.98E-05 | 4.347102 | 1.404372 | 1.08135 | CYB5A |
| 225038_s_at | 3.23E-04 | 6.84E-06 | 5.037873 | 3.617247 | 1.082368 | SURF6 |
| 236653_at | 4.13E-02 | 4.41E-03 | 2.985956 | -2.46805 | 1.083746 | LINC00662 |
| 219851_at | 3.18E-03 | 1.34E-04 | 4.14727 | 0.788928 | 1.085324 | ZNF613 |
| 211215_x_at | 8.23E-02 | 1.25E-02 | 2.594796 | -3.40625 | 1.085751 | DIO2 |
| 205248_at | 2.74E-02 | 2.48E-03 | 3.190968 | -1.94029 | 1.086247 | DOPEY2 |
| 210981_s_at | 1.85E-05 | 1.67E-07 | 6.098796 | 7.180841 | 1.086595 | GRK6 |
| 226459_at | 2.82E-02 | 2.58E-03 | 3.177242 | -1.97634 | 1.086986 | PIK3AP1 |
| 1554001_at | 2.49E-02 | 2.15E-03 | 3.24036 | -1.80972 | 1.087339 | TRIM37 |
| 236585_at | 7.51E-03 | 4.22E-04 | 3.784017 | -0.29379 | 1.087355 | RP5-894A10.6 |
| 204756_at | 2.29E-05 | 2.23E-07 | 6.016795 | 6.901273 | 1.08795 | MAP2K5 |
| 203755_at | 6.67E-03 | 3.59E-04 | 3.836317 | -0.14103 | 1.088084 | BUB1B |
| 204165_at | 8.80E-04 | 2.50E-05 | 4.655619 | 2.378016 | 1.089154 | WASF1 |
| 238332_at | 1.14E-01 | 2.15E-02 | 2.375526 | -3.88884 | 1.090189 | ANKRD29 |
| 242888_at | 1.43E-01 | 3.29E-02 | 2.195711 | -4.2594 | 1.090349 | PRRT3-AS1 |
| 243362_s_at | 5.22E-03 | 2.57E-04 | 3.942167 | 0.17147 | 1.091526 | LEF1-AS1 |
| 201088_at | 2.25E-04 | 4.25E-06 | 5.175718 | 4.071481 | 1.092923 | KPNA2 |
| 204170_s_at | 6.44E-02 | 8.45E-03 | 2.744911 | -3.05744 | 1.093076 | CKS2 |
| 235191_at | 1.56E-02 | 1.14E-03 | 3.458227 | -1.2189 | 1.093233 | LINC00662 |
| 230216_at | 3.98E-03 | 1.80E-04 | 4.054306 | 0.507182 | 1.093768 | HECTD4 |
| 227380_x_at | 8.58E-05 | 1.24E-06 | 5.529653 | 5.251953 | 1.093938 | C16orf13 |
| 223538_at | 1.49E-02 | 1.07E-03 | 3.478416 | -1.16298 | 1.094051 | SERF1A |
| 238165_at | 5.77E-02 | 7.16E-03 | 2.807375 | -2.90809 | 1.094732 | PDZRN3-AS1 |
| 228238_at | 1.81E-03 | 6.43E-05 | 4.371883 | 1.481573 | 1.095676 | GAS5 |
| 239450_at | 5.50E-04 | 1.35E-05 | 4.838236 | 2.965986 | 1.096009 | NDUFV2-AS1 |
| 209971_x_at | 1.45E-04 | 2.45E-06 | 5.334989 | 4.600386 | 1.096277 | AIMP2 |
| 210757_x_at | 5.12E-03 | 2.50E-04 | 3.951388 | 0.198898 | 1.096379 | DAB2 |
| 221646_s_at | 4.85E-03 | 2.33E-04 | 3.97348 | 0.264741 | 1.097508 | ZDHHC11 |
| 224650_at | 1.46E-04 | 2.47E-06 | 5.332381 | 4.591693 | 1.098093 | MAL2 |
| 230624_at | 2.47E-03 | 9.56E-05 | 4.250695 | 1.105846 | 1.098944 | SLC25A27 |
| 228260_at | 3.95E-02 | 4.13E-03 | 3.009186 | -2.40943 | 1.099608 | ELAVL2 |
| 212995_x_at | 6.29E-06 | 4.42E-08 | 6.472204 | 8.458109 | 1.102646 | MZT2A |
| 203354_s_at | 2.97E-02 | 2.76E-03 | 3.15318 | -2.0393 | 1.104567 | PSD3 |
| 216905_s_at | 1.16E-04 | 1.83E-06 | 5.419043 | 4.881086 | 1.104756 | ST14 |
| 229156_s_at | 4.53E-02 | 5.05E-03 | 2.936514 | -2.59177 | 1.10586 | PRKAG2-AS1 |
| 235881_at | 4.99E-04 | 1.19E-05 | 4.875259 | 3.086128 | 1.107391 | FMNL2 |
| 206261_at | 2.19E-02 | 1.81E-03 | 3.300532 | -1.64893 | 1.107419 | ZNF239 |
| 218613_at | 2.66E-02 | 2.37E-03 | 3.206703 | -1.89883 | 1.107849 | PSD3 |
| 208466_at | 1.71E-04 | 3.02E-06 | 5.274366 | 4.39859 | 1.108961 | RAB3D |
| 1554383_a_at | 6.42E-04 | 1.65E-05 | 4.779717 | 2.776718 | 1.109776 | TRAM2 |
| 221119_at | 5.04E-03 | 2.45E-04 | 3.957544 | 0.217227 | 1.110086 | ARHGEF38 |
| 200874_s_at | 9.72E-04 | 2.86E-05 | 4.616069 | 2.251752 | 1.110257 | MIR1292 |
| 208851_s_at | 5.54E-02 | 6.72E-03 | 2.830879 | -2.85127 | 1.110959 | THY1 |
| 228273_at | 3.49E-02 | 3.48E-03 | 3.071592 | -2.25044 | 1.111002 | PRR11 |
| 220192_x_at | 3.00E-04 | 6.17E-06 | 5.067761 | 3.715436 | 1.111394 | SPDEF |
| 1559363_at | 3.45E-02 | 3.41E-03 | 3.078066 | -2.23382 | 1.112079 | LINC01146 |
| 202437_s_at | 9.80E-02 | 1.66E-02 | 2.481167 | -3.66043 | 1.112565 | CYP1B1 |
| 219518_s_at | 1.19E-03 | 3.71E-05 | 4.538277 | 2.004596 | 1.113011 | ELL3 |
| 238333_s_at | 2.33E-02 | 1.97E-03 | 3.270471 | -1.72949 | 1.113135 | PAOX |
| 242794_at | 2.24E-04 | 4.20E-06 | 5.179459 | 4.083856 | 1.113253 | MAML3 |
| 212364_at | 1.36E-03 | 4.45E-05 | 4.483729 | 1.832266 | 1.113258 | MYO1B |
| 217516_x_at | 7.34E-05 | 1.01E-06 | 5.587602 | 5.446877 | 1.113309 | ARVCF |
| 204514_at | 1.94E-05 | 1.80E-07 | 6.077129 | 7.106932 | 1.114498 | DPH2 |
| 203304_at | 1.58E-01 | 3.96E-02 | 2.113994 | -4.41994 | 1.115284 | BAMBI |
| 205750_at | 8.87E-04 | 2.53E-05 | 4.652859 | 2.369193 | 1.115879 | BPHL |
| 227996_at | 2.03E-04 | 3.73E-06 | 5.214034 | 4.198343 | 1.116045 | FARP1 |
| 229407_at | 9.33E-02 | 1.52E-02 | 2.516695 | -3.58189 | 1.116166 | SDK1 |
| 222548_s_at | 4.88E-05 | 5.88E-07 | 5.742479 | 5.969702 | 1.116287 | MAP4K4 |
| 223427_s_at | 2.48E-02 | 2.14E-03 | 3.24197 | -1.80544 | 1.116573 | EPB41L4B |
| 227739_at | 4.15E-04 | 9.44E-06 | 4.943775 | 3.309248 | 1.117971 | NDOR1 |
| 218387_s_at | 1.86E-03 | 6.66E-05 | 4.361465 | 1.449095 | 1.118219 | PGLS |
| 202709_at | 7.54E-02 | 1.09E-02 | 2.648133 | -3.28398 | 1.118433 | FMOD |
| 215747_s_at | 6.96E-04 | 1.85E-05 | 4.746412 | 2.66935 | 1.118771 | RCC1 |
| 236278_at | 1.17E-03 | 3.63E-05 | 4.545048 | 2.026043 | 1.118931 | HIST1H3E |
| 220988_s_at | 4.25E-02 | 4.60E-03 | 2.970492 | -2.5069 | 1.119193 | C1QTNF3 |
| 222862_s_at | 1.51E-01 | 3.62E-02 | 2.154297 | -4.34139 | 1.119217 | AK5 |
| 223194_s_at | 1.51E-04 | 2.58E-06 | 5.319762 | 4.549647 | 1.121179 | SLC22A23 |
| 239921_at | 8.85E-02 | 1.40E-02 | 2.5494 | -3.50884 | 1.123644 | COL28A1 |
| 227220_at | 9.92E-05 | 1.51E-06 | 5.472738 | 5.060918 | 1.124105 | NFXL1 |
| 1568604_a_at | 1.68E-01 | 4.48E-02 | 2.059494 | -4.5242 | 1.124945 | CADPS |
| 243611_at | 4.38E-02 | 4.81E-03 | 2.953987 | -2.54821 | 1.125813 | NSD1 |
| 214472_at | 9.50E-03 | 5.80E-04 | 3.681084 | -0.59113 | 1.126847 | HIST1H2AD |
| 212838_at | 1.31E-02 | 8.94E-04 | 3.538317 | -0.99591 | 1.128998 | DNMBP |
| 229047_at | 4.89E-02 | 5.65E-03 | 2.89543 | -2.69349 | 1.129297 | PLEKHB1 |
| 226548_at | 2.19E-02 | 1.81E-03 | 3.299879 | -1.65069 | 1.129457 | SBK1 |
| 232191_at | 1.35E-01 | 2.93E-02 | 2.244965 | -4.16023 | 1.130898 | ERVH48-1 |
| 209190_s_at | 1.12E-05 | 8.99E-08 | 6.272637 | 7.77477 | 1.131147 | DIAPH1 |
| 217006_x_at | 2.13E-02 | 1.74E-03 | 3.313768 | -1.61331 | 1.132077 | FASN |
| 1557383_a_at | 1.16E-01 | 2.21E-02 | 2.363452 | -3.91445 | 1.132616 | RP5-1092A3.4 |
| 239580_at | 2.87E-03 | 1.17E-04 | 4.189194 | 0.916965 | 1.132719 | GUCY1A3 |
| 203693_s_at | 1.91E-05 | 1.73E-07 | 6.087907 | 7.143694 | 1.13346 | E2F3 |
| 224577_at | 2.56E-05 | 2.54E-07 | 5.979663 | 6.774826 | 1.13351 | ERGIC1 |
| 211709_s_at | 8.24E-03 | 4.79E-04 | 3.742901 | -0.4131 | 1.134228 | CLEC11A |
| 202368_s_at | 2.81E-04 | 5.66E-06 | 5.092793 | 3.797803 | 1.134916 | TRAM2 |
| 231046_at | 9.06E-04 | 2.61E-05 | 4.643416 | 2.339015 | 1.13526 | LINC01023 |
| 209850_s_at | 2.14E-03 | 7.99E-05 | 4.305589 | 1.275469 | 1.13554 | CDC42EP2 |
| 202533_s_at | 2.77E-03 | 1.11E-04 | 4.20463 | 0.964254 | 1.135892 | DHFR |
| 223478_at | 8.35E-03 | 4.88E-04 | 3.737086 | -0.42991 | 1.13608 | TIMM8B |
| 212193_s_at | 2.43E-03 | 9.37E-05 | 4.25692 | 1.125032 | 1.138173 | LARP1 |
| 233233_at | 5.06E-04 | 1.22E-05 | 4.869222 | 3.066519 | 1.138633 | RASSF3 |
| 223854_at | 5.74E-02 | 7.09E-03 | 2.810897 | -2.89959 | 1.138695 | PCDHB10 |
| 225777_at | 2.04E-03 | 7.50E-05 | 4.325141 | 1.336113 | 1.139306 | SAPCD2 |
| 209166_s_at | 6.22E-06 | 4.33E-08 | 6.477656 | 8.476794 | 1.139965 | MAN2B1 |
| 1564907_s_at | 1.91E-02 | 1.49E-03 | 3.367079 | -1.46896 | 1.141192 | MATR3 |
| 219857_at | 2.11E-02 | 1.70E-03 | 3.321042 | -1.5937 | 1.141272 | PLEKHS1 |
| 220342_x_at | 4.64E-03 | 2.21E-04 | 3.989417 | 0.312354 | 1.142218 | EDEM3 |
| 48808_at | 5.18E-03 | 2.54E-04 | 3.946395 | 0.184042 | 1.142412 | DHFR |
| 219438_at | 1.38E-01 | 3.08E-02 | 2.224155 | -4.20235 | 1.142855 | NKAIN1 |
| 205110_s_at | 5.83E-04 | 1.45E-05 | 4.81663 | 2.896017 | 1.143131 | FGF13 |
| 204700_x_at | 9.09E-03 | 5.47E-04 | 3.699948 | -0.53697 | 1.146647 | DIEXF |
| 223361_at | 1.62E-02 | 1.19E-03 | 3.443644 | -1.25918 | 1.147603 | ABRACL |
| 230784_at | 6.43E-03 | 3.42E-04 | 3.851605 | -0.09617 | 1.147663 | PRAC1 |
| 223275_at | 9.06E-03 | 5.45E-04 | 3.701251 | -0.53323 | 1.147779 | PRMT6 |
| 205879_x_at | 8.60E-02 | 1.34E-02 | 2.567224 | -3.46872 | 1.147958 | RET |
| 228114_x_at | 3.45E-06 | 2.01E-08 | 6.692717 | 9.214213 | 1.148706 | C16orf13 |
| 219127_at | 1.10E-02 | 7.08E-04 | 3.615437 | -0.77838 | 1.152442 | PRR15L |
| 223700_at | 4.14E-02 | 4.42E-03 | 2.984908 | -2.47069 | 1.154627 | MND1 |
| 211303_x_at | 1.77E-01 | 4.94E-02 | 2.014938 | -4.60775 | 1.1547 | FOLH1B |
| 203023_at | 1.52E-02 | 1.10E-03 | 3.468823 | -1.18958 | 1.15633 | NOP16 |
| 226828_s_at | 2.50E-02 | 2.16E-03 | 3.238274 | -1.81526 | 1.157538 | HEYL |
| 238681_at | 2.10E-02 | 1.70E-03 | 3.322352 | -1.59017 | 1.159419 | GDPD1 |
| 236328_at | 3.60E-03 | 1.57E-04 | 4.097151 | 0.636658 | 1.163446 | ZNF285 |
| 242313_at | 1.35E-02 | 9.32E-04 | 3.524616 | -1.03427 | 1.164014 | LOC728730 |
| 218788_s_at | 4.73E-03 | 2.27E-04 | 3.981974 | 0.290107 | 1.165621 | SMYD3 |
| 209152_s_at | 1.18E-08 | 1.55E-11 | 8.727003 | 16.10594 | 1.166564 | TCF3 |
| 228624_at | 2.68E-03 | 1.06E-04 | 4.218191 | 1.005866 | 1.168803 | TMEM144 |
| 201767_s_at | 2.60E-08 | 3.86E-11 | 8.464147 | 15.23262 | 1.168872 | ELAC2 |
| 212897_at | 4.24E-02 | 4.57E-03 | 2.972744 | -2.50125 | 1.172084 | CDK19 |
| 225220_at | 2.76E-03 | 1.10E-04 | 4.206461 | 0.96987 | 1.172154 | SNHG8 |
| 34260_at | 9.18E-06 | 6.99E-08 | 6.34343 | 8.017022 | 1.17224 | TELO2 |
| 221845_s_at | 4.58E-04 | 1.07E-05 | 4.907368 | 3.190565 | 1.1735 | CLPB |
| 212314_at | 1.82E-02 | 1.40E-03 | 3.387729 | -1.41267 | 1.173797 | SEL1L3 |
| 204717_s_at | 8.91E-03 | 5.31E-04 | 3.709459 | -0.50961 | 1.174034 | SLC29A2 |
| 225625_at | 3.34E-04 | 7.17E-06 | 5.024135 | 3.572171 | 1.176423 | ALKBH2 |
| 224962_at | 2.71E-07 | 8.14E-10 | 7.595361 | 12.30263 | 1.176462 | C9orf69 |
| 1554918_a_at | 5.13E-04 | 1.24E-05 | 4.864014 | 3.049607 | 1.176679 | ABCC4 |
| 225499_at | 2.14E-02 | 1.75E-03 | 3.312108 | -1.61778 | 1.177728 | RALGAPA2 |
| 227378_x_at | 1.63E-04 | 2.84E-06 | 5.292764 | 4.459769 | 1.177978 | C16orf13 |
| 224729_s_at | 1.78E-06 | 8.78E-09 | 6.925861 | 10.01382 | 1.178049 | ATPAF1 |
| 230110_at | 3.22E-02 | 3.09E-03 | 3.113134 | -2.14339 | 1.180147 | MCOLN2 |
| 231726_at | 8.97E-03 | 5.36E-04 | 3.70642 | -0.51836 | 1.182126 | PCDHB14 |
| 91816_f_at | 3.53E-02 | 3.53E-03 | 3.066052 | -2.26464 | 1.183328 | MEX3D |
| 216348_at | 2.46E-04 | 4.77E-06 | 5.142751 | 3.962533 | 1.183403 | RPS17P5 |
| 212311_at | 3.30E-02 | 3.22E-03 | 3.098605 | -2.18094 | 1.186575 | SEL1L3 |
| 224598_at | 1.65E-04 | 2.88E-06 | 5.288418 | 4.445314 | 1.187141 | MGAT4B |
| 228788_at | 2.00E-04 | 3.65E-06 | 5.219733 | 4.217234 | 1.187478 | YPEL1 |
| 219676_at | 2.00E-05 | 1.87E-07 | 6.065801 | 7.068302 | 1.188119 | ZSCAN16 |
| 1556285_s_at | 5.08E-04 | 1.23E-05 | 4.867138 | 3.059751 | 1.189064 | PPA2 |
| 205347_s_at | 7.41E-02 | 1.06E-02 | 2.658676 | -3.25959 | 1.192507 | TMSB15A |
| 225300_at | 6.52E-03 | 3.48E-04 | 3.846 | -0.11263 | 1.192709 | KNSTRN |
| 203196_at | 7.30E-05 | 1.00E-06 | 5.590019 | 5.455014 | 1.194111 | ABCC4 |
| 201796_s_at | 1.19E-06 | 5.20E-09 | 7.073101 | 10.51847 | 1.195779 | VARS |
| 227201_at | 4.49E-02 | 5.00E-03 | 2.94043 | -2.58202 | 1.199525 | LINC01128 |
| 1566557_at | 1.48E-03 | 4.96E-05 | 4.450523 | 1.727769 | 1.201015 | BAIAP2-AS1 |
| 238101_at | 3.39E-03 | 1.45E-04 | 4.121642 | 0.710957 | 1.201093 | BEND4 |
| 205483_s_at | 4.68E-02 | 5.31E-03 | 2.918122 | -2.63743 | 1.201603 | ISG15 |
| 221539_at | 3.95E-03 | 1.78E-04 | 4.057826 | 0.517795 | 1.204968 | EIF4EBP1 |
| 229954_at | 4.20E-03 | 1.93E-04 | 4.032066 | 0.440234 | 1.207862 | CHDH |
| 241310_at | 1.66E-02 | 1.24E-03 | 3.430189 | -1.29625 | 1.20887 | NEK5 |
| 225581_s_at | 7.30E-04 | 1.95E-05 | 4.729739 | 2.615701 | 1.210054 | MRPL50 |
| 212449_s_at | 6.83E-04 | 1.80E-05 | 4.754122 | 2.694183 | 1.21103 | LYPLA1 |
| 223775_at | 6.58E-02 | 8.77E-03 | 2.730386 | -3.09182 | 1.211132 | HHIP |
| 224603_at | 1.30E-05 | 1.08E-07 | 6.222056 | 7.601807 | 1.213349 | SNHG16 |
| 45526_g_at | 1.81E-06 | 8.94E-09 | 6.92073 | 9.996228 | 1.216702 | NAA60 |
| 228560_at | 1.10E-02 | 7.06E-04 | 3.616579 | -0.77514 | 1.217528 | CHDH |
| 227722_at | 2.67E-02 | 2.39E-03 | 3.204154 | -1.90556 | 1.217577 | RPS23 |
| 225520_at | 1.53E-03 | 5.16E-05 | 4.438593 | 1.690304 | 1.218037 | LOC100996643 |
| 230641_at | 1.02E-02 | 6.40E-04 | 3.648833 | -0.68336 | 1.219121 | LOC100505938 |
| 228654_at | 1.96E-03 | 7.11E-05 | 4.341257 | 1.386191 | 1.219833 | SPIN4 |
| 218911_at | 1.13E-03 | 3.46E-05 | 4.558918 | 2.070018 | 1.22326 | YEATS4 |
| 229350_x_at | 1.05E-03 | 3.16E-05 | 4.586187 | 2.156625 | 1.224521 | PARP10 |
| 200875_s_at | 2.18E-09 | 2.08E-12 | 9.314399 | 18.0304 | 1.225228 | MIR1292 |
| 211421_s_at | 5.85E-02 | 7.31E-03 | 2.799506 | -2.92703 | 1.225389 | RET |
| 227690_at | 2.28E-02 | 1.91E-03 | 3.280847 | -1.70174 | 1.226533 | GABRB3 |
| 227830_at | 1.15E-01 | 2.21E-02 | 2.364764 | -3.91167 | 1.226638 | GABRB3 |
| 201761_at | 2.04E-05 | 1.93E-07 | 6.057327 | 7.039409 | 1.226853 | MTHFD2 |
| 220679_s_at | 7.74E-02 | 1.13E-02 | 2.633627 | -3.31742 | 1.227766 | CDH7 |
| 242153_at | 1.10E-05 | 8.82E-08 | 6.27786 | 7.792635 | 1.228184 | LARP1B |
| 210787_s_at | 1.32E-03 | 4.27E-05 | 4.496197 | 1.871582 | 1.228217 | CAMKK2 |
| 236926_at | 1.25E-07 | 2.93E-10 | 7.8845 | 13.28419 | 1.230068 | TBX1 |
| 205850_s_at | 1.54E-01 | 3.75E-02 | 2.1381 | -4.3731 | 1.231017 | GABRB3 |
| 214710_s_at | 1.07E-02 | 6.82E-04 | 3.628148 | -0.74227 | 1.231274 | CCNB1 |
| 209708_at | 4.01E-02 | 4.23E-03 | 3.001344 | -2.42926 | 1.231906 | MOXD1 |
| 209806_at | 3.61E-03 | 1.58E-04 | 4.095475 | 0.631579 | 1.232422 | HIST1H2BK |
| 220541_at | 5.79E-02 | 7.18E-03 | 2.806211 | -2.91089 | 1.233165 | MMP26 |
| 220245_at | 6.25E-02 | 8.09E-03 | 2.761173 | -3.01879 | 1.234136 | SLC45A2 |
| 202005_at | 4.16E-04 | 9.47E-06 | 4.942684 | 3.305685 | 1.234298 | ST14 |
| 219978_s_at | 6.32E-03 | 3.33E-04 | 3.859815 | -0.07204 | 1.237317 | NUSAP1 |
| 240781_x_at | 4.64E-03 | 2.21E-04 | 3.989438 | 0.312418 | 1.237757 | DCUN1D1 |
| 242571_at | 2.60E-03 | 1.02E-04 | 4.229716 | 1.041276 | 1.237915 | REPS2 |
| 213582_at | 1.12E-02 | 7.23E-04 | 3.608906 | -0.79691 | 1.237922 | ATP11A |
| 37512_at | 1.50E-01 | 3.58E-02 | 2.158577 | -4.33297 | 1.239675 | HSD17B6 |
| 64486_at | 1.74E-03 | 6.16E-05 | 4.385023 | 1.522582 | 1.241183 | CORO1B |
| 226039_at | 7.59E-03 | 4.28E-04 | 3.779488 | -0.30697 | 1.241193 | MGAT4A |
| 211419_s_at | 4.68E-02 | 5.30E-03 | 2.918486 | -2.63653 | 1.24212 | CHN2 |
| 229648_at | 4.06E-05 | 4.65E-07 | 5.809132 | 6.195463 | 1.243206 | ARHGAP32 |
| 227062_at | 1.35E-02 | 9.28E-04 | 3.526039 | -1.03029 | 1.243569 | MIR612 |
| 231856_at | 7.53E-04 | 2.04E-05 | 4.716458 | 2.573013 | 1.243571 | KIAA1244 |
| 206307_s_at | 5.16E-02 | 6.08E-03 | 2.868376 | -2.75992 | 1.243926 | FOXD1 |
| 203286_at | 4.72E-06 | 3.00E-08 | 6.580699 | 8.830041 | 1.244634 | RNF44 |
| 218684_at | 2.37E-04 | 4.55E-06 | 5.156073 | 4.006537 | 1.245294 | LRRC8D |
| 201308_s_at | 2.09E-04 | 3.87E-06 | 5.202705 | 4.160809 | 1.24652 | 42989 |
| 205698_s_at | 4.77E-02 | 5.45E-03 | 2.908266 | -2.66181 | 1.246861 | MAP2K6 |
| 219395_at | 3.82E-04 | 8.46E-06 | 4.975793 | 3.413848 | 1.25017 | ESRP2 |
| 226569_s_at | 1.78E-04 | 3.19E-06 | 5.259096 | 4.34785 | 1.250861 | CHTF18 |
| 1555743_s_at | 9.91E-03 | 6.12E-04 | 3.663303 | -0.64203 | 1.251086 | ERVH-6 |
| 229724_at | 1.01E-01 | 1.76E-02 | 2.458164 | -3.71083 | 1.252409 | GABRB3 |
| 201292_at | 2.00E-02 | 1.58E-03 | 3.345595 | -1.52731 | 1.252435 | TOP2A |
| 225752_at | 1.70E-03 | 5.96E-05 | 4.394933 | 1.553544 | 1.254379 | NIPA1 |
| 209781_s_at | 1.31E-01 | 2.81E-02 | 2.262987 | -4.1235 | 1.254436 | KHDRBS3 |
| 214598_at | 1.03E-01 | 1.80E-02 | 2.448989 | -3.73083 | 1.255048 | CLDN8 |
| 213166_x_at | 1.93E-05 | 1.76E-07 | 6.082778 | 7.126199 | 1.255521 | MIR4784 |
| 209172_s_at | 1.43E-02 | 1.01E-03 | 3.498418 | -1.10737 | 1.257682 | CENPF |
| 205288_at | 1.27E-02 | 8.59E-04 | 3.551628 | -0.95855 | 1.258588 | CDC14A |
| 222904_s_at | 2.07E-02 | 1.66E-03 | 3.328962 | -1.57232 | 1.260411 | TMC5 |
| 217266_at | 4.61E-05 | 5.44E-07 | 5.7642 | 6.043227 | 1.260541 | RPL15 |
| 222587_s_at | 1.77E-04 | 3.16E-06 | 5.261618 | 4.35623 | 1.263742 | GALNT7 |
| 226460_at | 1.88E-02 | 1.47E-03 | 3.372425 | -1.45441 | 1.267067 | FNIP2 |
| 235602_at | 3.45E-03 | 1.49E-04 | 4.113324 | 0.685698 | 1.267894 | TP53INP1 |
| 202241_at | 1.50E-02 | 1.08E-03 | 3.474815 | -1.17297 | 1.268618 | TRIB1 |
| 229072_at | 5.83E-03 | 3.00E-04 | 3.893697 | 0.027829 | 1.269766 | RAB30 |
| 218661_at | 1.80E-04 | 3.22E-06 | 5.256021 | 4.337638 | 1.273658 | NAA60 |
| 1559861_at | 2.20E-03 | 8.28E-05 | 4.29482 | 1.242117 | 1.275069 | LOC101928099 |
| 204402_at | 9.43E-05 | 1.41E-06 | 5.493297 | 5.129879 | 1.276952 | RHBDD3 |
| 231022_at | 2.16E-02 | 1.78E-03 | 3.306261 | -1.63353 | 1.277325 | OCLN |
| 224468_s_at | 6.76E-04 | 1.77E-05 | 4.758223 | 2.707397 | 1.278304 | C19orf48 |
| 204401_at | 9.71E-02 | 1.63E-02 | 2.488304 | -3.64473 | 1.278582 | KCNN4 |
| 204141_at | 1.43E-02 | 1.01E-03 | 3.49749 | -1.10996 | 1.281782 | TUBB2A |
| 222317_at | 2.76E-02 | 2.49E-03 | 3.188961 | -1.94556 | 1.283057 | PDE3B |
| 1559633_a_at | 1.24E-01 | 2.53E-02 | 2.308157 | -4.03041 | 1.283147 | CHRM3 |
| 205462_s_at | 2.57E-03 | 1.01E-04 | 4.235116 | 1.057882 | 1.283372 | HPCAL1 |
| 230302_at | 1.15E-02 | 7.49E-04 | 3.59718 | -0.83012 | 1.284279 | RP11-48B3.4 |
| 242626_at | 6.83E-02 | 9.28E-03 | 2.709113 | -3.14193 | 1.285367 | SAMD5 |
| 218039_at | 4.06E-02 | 4.30E-03 | 2.994877 | -2.44557 | 1.286244 | NUSAP1 |
| 203932_at | 1.34E-01 | 2.91E-02 | 2.24883 | -4.15238 | 1.286995 | HLA-DMB |
| 214404_x_at | 1.35E-05 | 1.13E-07 | 6.209138 | 7.557652 | 1.288745 | SPDEF |
| 229099_at | 6.35E-05 | 8.48E-07 | 5.638327 | 5.617824 | 1.289682 | C11orf83 |
| 203230_at | 6.19E-08 | 1.23E-10 | 8.130932 | 14.11603 | 1.291884 | DVL1 |
| 209581_at | 3.82E-02 | 3.93E-03 | 3.027238 | -2.36367 | 1.292428 | PLA2G16 |
| 1556185_a_at | 2.02E-02 | 1.60E-03 | 3.342089 | -1.5368 | 1.293423 | CTB-167B5.2 |
| 1569433_at | 6.44E-02 | 8.44E-03 | 2.745129 | -3.05692 | 1.294976 | SAMD5 |
| 218188_s_at | 2.03E-06 | 1.02E-08 | 6.882781 | 9.866102 | 1.295554 | TIMM13 |
| 219405_at | 1.00E-02 | 6.23E-04 | 3.657413 | -0.65887 | 1.295768 | TRIM68 |
| 233985_x_at | 8.23E-05 | 1.18E-06 | 5.543545 | 5.298644 | 1.29677 | PPP1R9A |
| 237159_x_at | 9.44E-05 | 1.41E-06 | 5.492735 | 5.127993 | 1.297779 | AP1S3 |
| 235102_x_at | 1.08E-01 | 1.96E-02 | 2.413543 | -3.80754 | 1.30042 | SNORD3A |
| 1555993_at | 6.50E-03 | 3.46E-04 | 3.847613 | -0.10789 | 1.301695 | CACNA1D |
| 218654_s_at | 1.26E-04 | 2.03E-06 | 5.388691 | 4.779605 | 1.302439 | MRPS33 |
| 213937_s_at | 4.11E-03 | 1.88E-04 | 4.041537 | 0.468722 | 1.306258 | FTSJ1 |
| 1554607_at | 7.17E-02 | 1.00E-02 | 2.679242 | -3.21181 | 1.307565 | CNKSR2 |
| 224807_at | 2.20E-06 | 1.14E-08 | 6.851943 | 9.760343 | 1.308086 | GRAMD1A |
| 212884_x_at | 1.03E-04 | 1.59E-06 | 5.459557 | 5.016738 | 1.308489 | APOE |
| 215108_x_at | 8.28E-02 | 1.26E-02 | 2.590534 | -3.41594 | 1.308613 | TOX3 |
| 1552283_s_at | 2.99E-04 | 6.13E-06 | 5.069729 | 3.721909 | 1.308778 | ZDHHC11 |
| 213280_at | 7.35E-04 | 1.97E-05 | 4.727271 | 2.607763 | 1.309118 | RAP1GAP2 |
| 232218_at | 6.32E-05 | 8.40E-07 | 5.641065 | 5.627057 | 1.310892 | RP11-339B21.15 |
| 203355_s_at | 3.66E-03 | 1.61E-04 | 4.088767 | 0.61127 | 1.311433 | PSD3 |
| 213553_x_at | 1.15E-02 | 7.51E-04 | 3.596105 | -0.83316 | 1.311957 | APOC1 |
| 229669_at | 1.52E-02 | 1.10E-03 | 3.468884 | -1.18941 | 1.312672 | LOC440416 |
| 1556639_at | 4.37E-02 | 4.79E-03 | 2.955903 | -2.54342 | 1.314545 | LOC100996455 |
| 212353_at | 3.44E-02 | 3.40E-03 | 3.079651 | -2.22975 | 1.315197 | SULF1 |
| 1559591_s_at | 2.87E-04 | 5.81E-06 | 5.085041 | 3.772285 | 1.318741 | CHDH |
| 210052_s_at | 5.92E-02 | 7.42E-03 | 2.793803 | -2.94074 | 1.319516 | TPX2 |
| 229912_at | 5.83E-02 | 7.26E-03 | 2.802162 | -2.92064 | 1.320401 | SDK1 |
| 1557598_at | 9.97E-02 | 1.71E-02 | 2.469186 | -3.68673 | 1.320978 | LOC101927870 |
| 221754_s_at | 1.13E-04 | 1.77E-06 | 5.428002 | 4.911064 | 1.32155 | CORO1B |
| 205619_s_at | 1.31E-02 | 8.92E-04 | 3.539126 | -0.99364 | 1.322021 | MEOX1 |
| 223500_at | 3.41E-02 | 3.36E-03 | 3.084008 | -2.21854 | 1.324675 | CPLX1 |
| 201577_at | 9.77E-06 | 7.50E-08 | 6.323362 | 7.948328 | 1.325653 | NME1 |
| 202345_s_at | 8.80E-02 | 1.39E-02 | 2.552397 | -3.50211 | 1.326192 | FABP5 |
| 218027_at | 8.29E-04 | 2.32E-05 | 4.678136 | 2.450078 | 1.326748 | MRPL15 |
| 212012_at | 2.11E-05 | 2.01E-07 | 6.045754 | 6.999958 | 1.33213 | PXDN |
| 221942_s_at | 7.18E-04 | 1.92E-05 | 4.735238 | 2.633388 | 1.332526 | GUCY1A3 |
| 209114_at | 5.47E-04 | 1.34E-05 | 4.841194 | 2.975575 | 1.334374 | TSPAN1 |
| 231725_at | 1.22E-01 | 2.43E-02 | 2.324316 | -3.99675 | 1.33538 | PCDHB2 |
| 203029_s_at | 4.29E-04 | 9.86E-06 | 4.93108 | 3.267832 | 1.336241 | PTPRN2 |
| 240124_at | 7.40E-02 | 1.06E-02 | 2.659617 | -3.25741 | 1.338209 | CTA-246H3.12 |
| 218388_at | 1.22E-05 | 9.90E-08 | 6.245368 | 7.681507 | 1.339067 | PGLS |
| 225418_at | 2.81E-04 | 5.62E-06 | 5.094798 | 3.804407 | 1.339402 | PVRL2 |
| 210612_s_at | 3.45E-05 | 3.76E-07 | 5.868732 | 6.397675 | 1.340077 | SYNJ2 |
| 203358_s_at | 3.99E-05 | 4.53E-07 | 5.816006 | 6.218768 | 1.34764 | EZH2 |
| 213234_at | 1.45E-02 | 1.02E-03 | 3.493156 | -1.12202 | 1.350991 | KIAA1467 |
| 229003_x_at | 4.84E-06 | 3.13E-08 | 6.569305 | 8.790973 | 1.351023 | FAM69B |
| 220721_at | 3.15E-03 | 1.32E-04 | 4.151248 | 0.801054 | 1.351251 | ZNF614 |
| 229530_at | 5.43E-05 | 6.77E-07 | 5.702283 | 5.833764 | 1.353931 | GUCY1A3 |
| 204042_at | 5.32E-05 | 6.60E-07 | 5.70943 | 5.857924 | 1.354146 | WASF3 |
| 227123_at | 2.00E-02 | 1.59E-03 | 3.344921 | -1.52913 | 1.355656 | RAB3B |
| 211706_s_at | 1.95E-02 | 1.53E-03 | 3.357019 | -1.49631 | 1.357514 | CDK19 |
| 212022_s_at | 4.92E-04 | 1.17E-05 | 4.88037 | 3.102738 | 1.357729 | MKI67 |
| 239660_at | 2.93E-02 | 2.71E-03 | 3.15955 | -2.02267 | 1.358239 | RALGAPA2 |
| 209925_at | 1.59E-03 | 5.47E-05 | 4.421023 | 1.635201 | 1.364614 | OCLN |
| 227406_at | 8.11E-04 | 2.26E-05 | 4.685832 | 2.474738 | 1.367447 | GABPB1-AS1 |
| 201013_s_at | 3.21E-06 | 1.84E-08 | 6.718146 | 9.301434 | 1.367803 | PAICS |
| 1568603_at | 5.55E-02 | 6.74E-03 | 2.829892 | -2.85366 | 1.371614 | CADPS |
| 225307_at | 5.55E-06 | 3.73E-08 | 6.519691 | 8.620875 | 1.373477 | ZNF511 |
| 226129_at | 4.92E-06 | 3.21E-08 | 6.562024 | 8.766005 | 1.378391 | FAM83H |
| 222305_at | 4.20E-03 | 1.93E-04 | 4.032888 | 0.442705 | 1.378811 | HK2 |
| 222771_s_at | 1.72E-04 | 3.06E-06 | 5.271085 | 4.387685 | 1.381864 | MYEF2 |
| 213498_at | 3.86E-03 | 1.73E-04 | 4.067059 | 0.545655 | 1.383434 | CREB3L1 |
| 241827_at | 3.64E-03 | 1.60E-04 | 4.091543 | 0.619673 | 1.392929 | ZNF615 |
| 1557986_s_at | 3.81E-05 | 4.30E-07 | 5.830775 | 6.268858 | 1.393535 | SMCR8 |
| 230509_at | 5.73E-03 | 2.91E-04 | 3.903026 | 0.055404 | 1.393701 | SNX22 |
| 205145_s_at | 1.88E-03 | 6.77E-05 | 4.356317 | 1.433056 | 1.394751 | MYL5 |
| 1552365_at | 9.00E-02 | 1.44E-02 | 2.538893 | -3.53238 | 1.394806 | SCIN |
| 217851_s_at | 1.13E-04 | 1.77E-06 | 5.42764 | 4.909851 | 1.395377 | SLMO2 |
| 1552898_a_at | 7.08E-02 | 9.84E-03 | 2.686659 | -3.19451 | 1.396055 | KCNG3 |
| 209340_at | 8.64E-05 | 1.26E-06 | 5.526124 | 5.240099 | 1.397062 | UAP1 |
| 203913_s_at | 1.63E-01 | 4.25E-02 | 2.08311 | -4.4793 | 1.400456 | HPGD |
| 218883_s_at | 1.67E-03 | 5.84E-05 | 4.40129 | 1.573422 | 1.401243 | CENPU |
| 244721_at | 1.49E-03 | 4.97E-05 | 4.450016 | 1.726177 | 1.405164 | TP53INP1 |
| 1564063_a_at | 2.81E-04 | 5.66E-06 | 5.092745 | 3.797646 | 1.405234 | ATP11B |
| 244052_at | 7.85E-07 | 3.06E-09 | 7.222163 | 11.02881 | 1.409055 | CBR4 |
| 214011_s_at | 5.62E-04 | 1.39E-05 | 4.830695 | 2.941552 | 1.413772 | NOP16 |
| 238824_at | 3.57E-05 | 3.93E-07 | 5.856137 | 6.354918 | 1.424583 | RPS29 |
| 1569112_at | 1.07E-01 | 1.95E-02 | 2.416012 | -3.80222 | 1.425085 | SLC44A5 |
| 223989_s_at | 1.31E-03 | 4.23E-05 | 4.499046 | 1.880573 | 1.425162 | REXO2 |
| 229964_at | 8.95E-04 | 2.56E-05 | 4.648583 | 2.355526 | 1.429319 | C9orf152 |
| 214774_x_at | 6.33E-02 | 8.25E-03 | 2.753706 | -3.03656 | 1.429393 | TOX3 |
| 239433_at | 1.52E-03 | 5.12E-05 | 4.440821 | 1.697297 | 1.42998 | LRRC8E |
| 218662_s_at | 4.67E-06 | 2.96E-08 | 6.584217 | 8.842103 | 1.431571 | NCAPG |
| 214403_x_at | 1.27E-06 | 5.62E-09 | 7.051104 | 10.44311 | 1.43188 | SPDEF |
| 237241_at | 2.83E-03 | 1.14E-04 | 4.195473 | 0.936192 | 1.432797 | ECT2 |
| 224396_s_at | 1.63E-02 | 1.20E-03 | 3.439968 | -1.26932 | 1.435315 | ASPN |
| 225147_at | 2.42E-04 | 4.69E-06 | 5.147385 | 3.977836 | 1.436978 | CYTH3 |
| 212218_s_at | 6.02E-03 | 3.12E-04 | 3.880765 | -0.01034 | 1.439051 | FASN |
| 228400_at | 4.99E-04 | 1.19E-05 | 4.875947 | 3.088363 | 1.439707 | SHROOM3 |
| 229309_at | 8.58E-05 | 1.24E-06 | 5.529249 | 5.250598 | 1.440026 | ADRB1 |
| 209113_s_at | 3.97E-04 | 8.88E-06 | 4.961437 | 3.366921 | 1.440347 | HMG20B |
| 201418_s_at | 5.99E-06 | 4.15E-08 | 6.48967 | 8.517972 | 1.445446 | SOX4 |
| 1557599_a_at | 1.81E-02 | 1.39E-03 | 3.391223 | -1.40312 | 1.448068 | LOC101927870 |
| 226803_at | 6.48E-04 | 1.67E-05 | 4.775367 | 2.762679 | 1.452616 | CHMP4C |
| 225589_at | 1.41E-05 | 1.19E-07 | 6.192529 | 7.500891 | 1.453016 | SH3RF1 |
| 228461_at | 2.67E-02 | 2.38E-03 | 3.20497 | -1.9034 | 1.45375 | SH3RF3 |
| 233220_at | 5.97E-02 | 7.52E-03 | 2.78883 | -2.95268 | 1.456398 | GRIN3A |
| 206351_s_at | 1.42E-02 | 1.00E-03 | 3.500215 | -1.10237 | 1.456933 | PEX10 |
| 236302_at | 4.17E-02 | 4.47E-03 | 2.981098 | -2.48027 | 1.459633 | PPM1E |
| 228494_at | 1.23E-04 | 1.97E-06 | 5.396878 | 4.806966 | 1.465053 | PPP1R9A |
| 222020_s_at | 2.23E-02 | 1.85E-03 | 3.291808 | -1.67236 | 1.466004 | LOC102725271 |
| 239752_at | 6.72E-04 | 1.76E-05 | 4.760608 | 2.715082 | 1.470372 | CECR2 |
| 209372_x_at | 3.97E-03 | 1.80E-04 | 4.055095 | 0.509563 | 1.471759 | TUBB2A |
| 226912_at | 5.70E-04 | 1.41E-05 | 4.825924 | 2.926103 | 1.47373 | ZDHHC23 |
| 236448_at | 1.03E-01 | 1.81E-02 | 2.446376 | -3.73651 | 1.47426 | UNC5A |
| 219874_at | 3.70E-05 | 4.17E-07 | 5.839882 | 6.299753 | 1.475537 | SLC12A8 |
| 1556905_at | 1.65E-02 | 1.23E-03 | 3.432369 | -1.29025 | 1.476012 | ZNF577 |
| 216250_s_at | 3.66E-02 | 3.71E-03 | 3.04861 | -2.30925 | 1.477383 | LPXN |
| 209825_s_at | 2.79E-05 | 2.90E-07 | 5.942352 | 6.647869 | 1.478332 | MIR3658 |
| 203139_at | 7.78E-03 | 4.42E-04 | 3.769441 | -0.33617 | 1.478788 | DAPK1 |
| 222216_s_at | 1.25E-07 | 2.95E-10 | 7.882332 | 13.27685 | 1.482956 | MRPL17 |
| 222608_s_at | 9.00E-03 | 5.39E-04 | 3.704954 | -0.52258 | 1.482999 | ANLN |
| 221558_s_at | 6.38E-02 | 8.33E-03 | 2.750072 | -3.04519 | 1.484489 | LEF1 |
| 204623_at | 7.78E-02 | 1.14E-02 | 2.630445 | -3.32473 | 1.48498 | TFF3 |
| 228095_at | 5.50E-07 | 1.98E-09 | 7.344195 | 11.44603 | 1.486046 | PHF14 |
| 231123_at | 3.01E-04 | 6.21E-06 | 5.065993 | 3.709623 | 1.486436 | TRIM36 |
| 224467_s_at | 5.13E-04 | 1.24E-05 | 4.863218 | 3.047021 | 1.487115 | PDCD2L |
| 205645_at | 7.28E-04 | 1.94E-05 | 4.731045 | 2.619901 | 1.487272 | REPS2 |
| 229544_at | 7.89E-06 | 5.87E-08 | 6.392447 | 8.184863 | 1.489649 | RP11-391M1.4 |
| 231249_at | 2.55E-04 | 4.98E-06 | 5.130101 | 3.92078 | 1.496272 | SZT2 |
| 223616_at | 1.26E-03 | 4.00E-05 | 4.515301 | 1.93191 | 1.496848 | ZNF649 |
| 227235_at | 5.65E-05 | 7.18E-07 | 5.685585 | 5.777345 | 1.499365 | GUCY1A3 |
| 216623_x_at | 5.44E-02 | 6.55E-03 | 2.840439 | -2.82806 | 1.505445 | TOX3 |
| 232014_at | 2.65E-05 | 2.67E-07 | 5.965499 | 6.726619 | 1.50778 | ZNF30 |
| 227725_at | 4.42E-02 | 4.88E-03 | 2.949025 | -2.5606 | 1.513157 | ST6GALNAC1 |
| 1558292_s_at | 9.03E-05 | 1.34E-06 | 5.507777 | 5.178479 | 1.513569 | PIGW |
| 222079_at | 2.01E-02 | 1.59E-03 | 3.344445 | -1.53042 | 1.514509 | ERG |
| 205426_s_at | 3.23E-05 | 3.49E-07 | 5.889969 | 6.469803 | 1.515717 | HIP1 |
| 221649_s_at | 1.04E-05 | 8.04E-08 | 6.304042 | 7.882211 | 1.518526 | PPAN |
| 207262_at | 1.88E-03 | 6.75E-05 | 4.357091 | 1.435467 | 1.52737 | APOF |
| 223423_at | 6.61E-04 | 1.72E-05 | 4.767375 | 2.736899 | 1.530052 | GPR160 |
| 1555236_a_at | 1.15E-01 | 2.19E-02 | 2.367478 | -3.90592 | 1.530376 | PGC |
| 225984_at | 5.99E-08 | 1.15E-10 | 8.150754 | 14.18273 | 1.530536 | PRKAA1 |
| 205339_at | 5.47E-06 | 3.65E-08 | 6.525702 | 8.641479 | 1.530928 | STIL |
| 228049_x_at | 3.84E-06 | 2.34E-08 | 6.650721 | 9.070174 | 1.540948 | SNHG19 |
| 204319_s_at | 1.00E-06 | 4.23E-09 | 7.130746 | 10.7159 | 1.547968 | RGS10 |
| 207828_s_at | 4.14E-03 | 1.90E-04 | 4.037621 | 0.456941 | 1.55196 | CENPF |
| 223278_at | 2.56E-02 | 2.24E-03 | 3.226988 | -1.84519 | 1.554203 | GJB2 |
| 226884_at | 1.10E-01 | 2.04E-02 | 2.397194 | -3.84263 | 1.555774 | LRRN1 |
| 217771_at | 4.92E-06 | 3.19E-08 | 6.563289 | 8.770344 | 1.558803 | GOLM1 |
| 219266_at | 2.79E-06 | 1.54E-08 | 6.76873 | 9.47494 | 1.559297 | ZNF350 |
| 205434_s_at | 2.65E-05 | 2.69E-07 | 5.963378 | 6.719402 | 1.562954 | AAK1 |
| 242345_at | 4.04E-02 | 4.27E-03 | 2.997244 | -2.4396 | 1.565954 | COL28A1 |
| 204040_at | 2.32E-04 | 4.42E-06 | 5.164629 | 4.034816 | 1.566321 | RNF144A |
| 225922_at | 3.85E-03 | 1.72E-04 | 4.067911 | 0.548227 | 1.566502 | FNIP2 |
| 205923_at | 1.71E-02 | 1.28E-03 | 3.41821 | -1.32918 | 1.576544 | RELN |
| 203953_s_at | 5.77E-03 | 2.94E-04 | 3.899485 | 0.044932 | 1.577039 | CLDN3 |
| 219308_s_at | 2.49E-02 | 2.15E-03 | 3.240039 | -1.81057 | 1.57714 | AK5 |
| 203215_s_at | 4.32E-03 | 2.00E-04 | 4.021618 | 0.408844 | 1.58314 | MYO6 |
| 244116_at | 1.27E-02 | 8.64E-04 | 3.549722 | -0.96391 | 1.585521 | LOC101927391 |
| 204305_at | 5.87E-02 | 7.34E-03 | 2.79806 | -2.93051 | 1.590623 | MIPEP |
| 206507_at | 1.38E-04 | 2.29E-06 | 5.353795 | 4.6631 | 1.592762 | ZSCAN12 |
| 212680_x_at | 1.04E-07 | 2.31E-10 | 7.951746 | 13.51165 | 1.599008 | PPP1R14B |
| 217912_at | 1.45E-07 | 3.72E-10 | 7.816817 | 13.05492 | 1.599589 | DUS1L |
| 238573_at | 1.60E-07 | 4.31E-10 | 7.775089 | 12.91341 | 1.604129 | OTUD7B |
| 200644_at | 1.25E-06 | 5.44E-09 | 7.060028 | 10.47368 | 1.607439 | MARCKSL1 |
| 209522_s_at | 9.88E-05 | 1.50E-06 | 5.475213 | 5.069217 | 1.608142 | CRAT |
| 226594_at | 4.09E-04 | 9.22E-06 | 4.950465 | 3.331087 | 1.608518 | ENTPD5 |
| 219787_s_at | 2.41E-04 | 4.65E-06 | 5.150029 | 3.986569 | 1.617088 | ECT2 |
| 219463_at | 8.22E-03 | 4.78E-04 | 3.743818 | -0.41044 | 1.618048 | LAMP5 |
| 207824_s_at | 3.01E-07 | 9.35E-10 | 7.556196 | 12.16928 | 1.618193 | MAZ |
| 204942_s_at | 1.81E-02 | 1.38E-03 | 3.39156 | -1.4022 | 1.618307 | ALDH3B2 |
| 233903_s_at | 2.59E-05 | 2.58E-07 | 5.975198 | 6.759629 | 1.622913 | ARHGEF26 |
| 210791_s_at | 5.35E-06 | 3.56E-08 | 6.532747 | 8.66563 | 1.629584 | ARHGAP32 |
| 206502_s_at | 4.47E-02 | 4.96E-03 | 2.942954 | -2.57574 | 1.629701 | INSM1 |
| 228653_at | 1.38E-02 | 9.58E-04 | 3.515604 | -1.05945 | 1.629987 | SAMD5 |
| 223743_s_at | 1.27E-04 | 2.06E-06 | 5.385179 | 4.767874 | 1.630958 | MRPL4 |
| 1554556_a_at | 9.86E-05 | 1.49E-06 | 5.476481 | 5.07347 | 1.632174 | ATP11B |
| 213812_s_at | 5.28E-03 | 2.61E-04 | 3.937826 | 0.158567 | 1.633787 | CAMKK2 |
| 219736_at | 4.78E-06 | 3.07E-08 | 6.574233 | 8.807869 | 1.634032 | TRIM36 |
| 223183_at | 2.34E-09 | 2.27E-12 | 9.288538 | 17.94652 | 1.641286 | AGPAT3 |
| 219750_at | 5.77E-04 | 1.43E-05 | 4.820827 | 2.909599 | 1.644109 | TMEM144 |
| 206275_s_at | 1.27E-03 | 4.03E-05 | 4.513425 | 1.92598 | 1.644446 | MICAL2 |
| 226226_at | 7.29E-02 | 1.03E-02 | 2.669216 | -3.23514 | 1.648129 | TMEM45B |
| 1560683_at | 6.91E-02 | 9.42E-03 | 2.703067 | -3.15612 | 1.652394 | NBEAP1 |
| 240304_s_at | 7.59E-03 | 4.28E-04 | 3.779824 | -0.30599 | 1.653536 | LOC102724257 |
| 218931_at | 4.76E-08 | 8.71E-11 | 8.230476 | 14.45064 | 1.663472 | RAB17 |
| 216251_s_at | 8.35E-07 | 3.34E-09 | 7.197081 | 10.94298 | 1.665982 | TTLL12 |
| 231966_at | 2.36E-07 | 6.91E-10 | 7.641649 | 12.46012 | 1.666552 | PPP1R9A |
| 1553172_at | 3.45E-05 | 3.77E-07 | 5.86811 | 6.395563 | 1.668757 | ZNF777 |
| 215785_s_at | 3.13E-05 | 3.35E-07 | 5.901399 | 6.508638 | 1.669277 | CYFIP2 |
| 231557_at | 8.72E-03 | 5.15E-04 | 3.719511 | -0.48065 | 1.670087 | LOC102724842 |
| 1560684_x_at | 6.05E-02 | 7.67E-03 | 2.781185 | -2.971 | 1.673026 | NBEAP1 |
| 237563_s_at | 7.32E-04 | 1.96E-05 | 4.728895 | 2.612986 | 1.673082 | RP11-295G20.2 |
| 219584_at | 7.64E-02 | 1.11E-02 | 2.641232 | -3.2999 | 1.677019 | PLA1A |
| 214000_s_at | 1.07E-06 | 4.56E-09 | 7.109594 | 10.64347 | 1.677384 | RGS10 |
| 1562969_at | 3.21E-04 | 6.75E-06 | 5.041673 | 3.629721 | 1.681165 | DNMBP |
| 1552742_at | 3.54E-02 | 3.53E-03 | 3.065651 | -2.26567 | 1.682406 | KCNH8 |
| 227429_at | 3.29E-06 | 1.89E-08 | 6.710059 | 9.273694 | 1.685635 | EFCAB4A |
| 201516_at | 7.55E-07 | 2.91E-09 | 7.23584 | 11.07559 | 1.686141 | SRM |
| 235591_at | 9.13E-02 | 1.47E-02 | 2.529721 | -3.55288 | 1.687038 | SSTR1 |
| 235763_at | 8.09E-02 | 1.22E-02 | 2.60467 | -3.38375 | 1.68793 | SLC44A5 |
| 209921_at | 4.38E-03 | 2.03E-04 | 4.016272 | 0.392801 | 1.690441 | SLC7A11 |
| 218638_s_at | 5.92E-02 | 7.42E-03 | 2.793805 | -2.94074 | 1.690953 | LOC100130872 |
| 213622_at | 4.03E-02 | 4.26E-03 | 2.99828 | -2.43699 | 1.696609 | COL9A2 |
| 1555826_at | 8.54E-04 | 2.41E-05 | 4.667455 | 2.415878 | 1.706047 | BIRC5 |
| 225681_at | 2.94E-02 | 2.72E-03 | 3.158005 | -2.0267 | 1.71681 | CTHRC1 |
| 205769_at | 7.54E-04 | 2.04E-05 | 4.716079 | 2.571796 | 1.721892 | SLC27A2 |
| 210809_s_at | 4.89E-02 | 5.66E-03 | 2.894949 | -2.69467 | 1.725958 | POSTN |
| 242488_at | 4.98E-02 | 5.79E-03 | 2.886156 | -2.71631 | 1.729336 | CHRM3 |
| 230766_at | 6.38E-04 | 1.64E-05 | 4.781972 | 2.783996 | 1.730874 | GART |
| 219521_at | 1.21E-04 | 1.92E-06 | 5.404956 | 4.833971 | 1.731111 | B3GAT1 |
| 203911_at | 4.57E-05 | 5.38E-07 | 5.767621 | 6.05481 | 1.732162 | RAP1GAP |
| 206110_at | 8.49E-03 | 4.98E-04 | 3.730287 | -0.44956 | 1.73388 | HIST1H3A |
| 230875_s_at | 3.29E-03 | 1.40E-04 | 4.133951 | 0.74838 | 1.736672 | ATP11A |
| 1555842_at | 5.09E-06 | 3.36E-08 | 6.549147 | 8.721858 | 1.737356 | CYTH2 |
| 205768_s_at | 2.59E-04 | 5.12E-06 | 5.122221 | 3.894786 | 1.740484 | SLC27A2 |
| 215011_at | 6.19E-03 | 3.23E-04 | 3.870096 | -0.04179 | 1.74287 | SNHG3 |
| 222774_s_at | 2.86E-02 | 2.63E-03 | 3.170471 | -1.99409 | 1.75029 | NETO2 |
| 224870_at | 5.85E-04 | 1.46E-05 | 4.814884 | 2.890366 | 1.751095 | DANCR |
| 226237_at | 9.06E-03 | 5.44E-04 | 3.701793 | -0.53167 | 1.759844 | COL8A1 |
| 234951_s_at | 2.15E-02 | 1.76E-03 | 3.309852 | -1.62386 | 1.763515 | COL12A1 |
| 1567906_at | 9.81E-05 | 1.49E-06 | 5.47827 | 5.079469 | 1.767049 | SOX4 |
| 225666_at | 5.58E-06 | 3.77E-08 | 6.517132 | 8.612101 | 1.767187 | TMTC4 |
| 215300_s_at | 5.24E-02 | 6.22E-03 | 2.860034 | -2.78031 | 1.768717 | FMO5 |
| 1557080_s_at | 1.85E-02 | 1.43E-03 | 3.381454 | -1.42979 | 1.768945 | ITGBL1 |
| 216803_at | 7.32E-03 | 4.08E-04 | 3.794777 | -0.26246 | 1.769923 | PDLIM5 |
| 225762_x_at | 5.97E-06 | 4.12E-08 | 6.491892 | 8.525585 | 1.7786 | RNA45S5 |
| 223235_s_at | 1.26E-04 | 2.03E-06 | 5.388422 | 4.778706 | 1.779543 | SMOC2 |
| 240838_s_at | 1.68E-03 | 5.89E-05 | 4.398492 | 1.564673 | 1.780205 | LOC145837 |
| 230075_at | 3.15E-03 | 1.32E-04 | 4.15086 | 0.79987 | 1.782592 | RAB39B |
| 212472_at | 2.16E-04 | 4.02E-06 | 5.192194 | 4.126001 | 1.783724 | MICAL2 |
| 203954_x_at | 1.84E-04 | 3.31E-06 | 5.24797 | 4.310907 | 1.78526 | CLDN3 |
| 1552257_a_at | 2.81E-07 | 8.48E-10 | 7.583624 | 12.26268 | 1.791633 | TTLL12 |
| 219580_s_at | 2.17E-03 | 8.13E-05 | 4.300387 | 1.259352 | 1.79179 | TMC5 |
| 207147_at | 8.32E-03 | 4.86E-04 | 3.738604 | -0.42552 | 1.793101 | DLX2 |
| 203216_s_at | 9.91E-05 | 1.51E-06 | 5.473241 | 5.062605 | 1.79735 | MYO6 |
| 221576_at | 6.94E-03 | 3.79E-04 | 3.818495 | -0.19321 | 1.802938 | GDF15 |
| 202790_at | 3.63E-03 | 1.60E-04 | 4.092041 | 0.621182 | 1.805221 | CLDN7 |
| 235937_at | 2.71E-04 | 5.41E-06 | 5.106173 | 3.841879 | 1.806005 | OCLN |
| 231964_at | 6.19E-04 | 1.57E-05 | 4.793486 | 2.821181 | 1.838278 | BICD1 |
| 210480_s_at | 1.56E-03 | 5.29E-05 | 4.430984 | 1.666431 | 1.838963 | MYO6 |
| 229002_at | 6.14E-05 | 8.04E-07 | 5.653202 | 5.668008 | 1.842744 | FAM69B |
| 202095_s_at | 3.34E-04 | 7.16E-06 | 5.024384 | 3.572987 | 1.844166 | BIRC5 |
| 216990_at | 2.86E-07 | 8.72E-10 | 7.575699 | 12.23569 | 1.863661 | GART |
| 236121_at | 8.56E-02 | 1.33E-02 | 2.56995 | -3.46256 | 1.866026 | OR51E2 |
| 74694_s_at | 4.33E-06 | 2.69E-08 | 6.611744 | 8.936498 | 1.867654 | RABEP2 |
| 209504_s_at | 3.10E-03 | 1.29E-04 | 4.157457 | 0.819987 | 1.873785 | PLEKHB1 |
| 220334_at | 4.04E-03 | 1.84E-04 | 4.048118 | 0.488539 | 1.878065 | RGS17 |
| 204179_at | 2.61E-03 | 1.03E-04 | 4.228346 | 1.037064 | 1.891484 | MB |
| 36830_at | 4.07E-02 | 4.32E-03 | 2.993334 | -2.44947 | 1.89187 | MIPEP |
| 1554102_a_at | 1.25E-05 | 1.02E-07 | 6.237414 | 7.65431 | 1.900209 | TMTC4 |
| 201340_s_at | 2.81E-04 | 5.64E-06 | 5.093615 | 3.80051 | 1.905638 | ENC1 |
| 207998_s_at | 3.04E-02 | 2.85E-03 | 3.142156 | -2.06804 | 1.914758 | CACNA1D |
| 212252_at | 1.04E-05 | 8.05E-08 | 6.303768 | 7.881276 | 1.91993 | CAMKK2 |
| 221018_s_at | 2.40E-02 | 2.05E-03 | 3.256965 | -1.76554 | 1.920803 | TDRD1 |
| 204394_at | 2.17E-04 | 4.05E-06 | 5.190057 | 4.118926 | 1.927186 | SLC43A1 |
| 205757_at | 4.09E-05 | 4.69E-07 | 5.806361 | 6.186069 | 1.943834 | ENTPD5 |
| 205073_at | 7.57E-03 | 4.26E-04 | 3.780804 | -0.30314 | 1.951535 | CYP2J2 |
| 231879_at | 8.76E-03 | 5.20E-04 | 3.716797 | -0.48847 | 1.952981 | COL12A1 |
| 223642_at | 2.77E-02 | 2.51E-03 | 3.187091 | -1.95048 | 1.956612 | ZIC2 |
| 1552735_at | 4.31E-03 | 1.99E-04 | 4.022986 | 0.412953 | 1.962015 | PCDHGA4 |
| 223897_at | 2.51E-03 | 9.75E-05 | 4.244626 | 1.087151 | 1.967672 | ZNF765 |
| 204926_at | 3.66E-03 | 1.61E-04 | 4.088802 | 0.611376 | 1.973891 | INHBA |
| 202431_s_at | 4.30E-04 | 9.90E-06 | 4.929889 | 3.263949 | 1.979693 | MYC |
| 218469_at | 3.44E-03 | 1.49E-04 | 4.114012 | 0.687787 | 1.987581 | GREM1 |
| 204875_s_at | 3.44E-03 | 1.49E-04 | 4.113835 | 0.68725 | 1.988861 | GMDS |
| 225541_at | 2.90E-06 | 1.63E-08 | 6.752289 | 9.418547 | 1.989223 | RPL22L1 |
| 220226_at | 1.41E-02 | 9.83E-04 | 3.506963 | -1.08357 | 1.993605 | TRPM8 |
| 216920_s_at | 7.85E-05 | 1.11E-06 | 5.561194 | 5.357998 | 2.005804 | TARP |
| 205357_s_at | 3.11E-02 | 2.95E-03 | 3.130339 | -2.09878 | 2.007211 | AGTR1 |
| 204052_s_at | 3.55E-03 | 1.54E-04 | 4.102971 | 0.654294 | 2.016532 | SFRP4 |
| 1555173_at | 1.64E-06 | 7.74E-09 | 6.961159 | 10.13484 | 2.017414 | STX19 |
| 219477_s_at | 6.24E-05 | 8.22E-07 | 5.647056 | 5.64727 | 2.019227 | LOC101930578 |
| 223581_at | 3.75E-06 | 2.24E-08 | 6.662511 | 9.11061 | 2.019944 | ZNF577 |
| 1557203_at | 1.44E-03 | 4.75E-05 | 4.463582 | 1.768828 | 2.020643 | PABPC1L2A |
| 1555778_a_at | 7.48E-03 | 4.19E-04 | 3.786402 | -0.28685 | 2.021987 | POSTN |
| 234197_at | 7.86E-03 | 4.48E-04 | 3.76483 | -0.34955 | 2.026891 | ACSM1 |
| 228241_at | 4.12E-02 | 4.39E-03 | 2.987607 | -2.46389 | 2.027611 | AGR3 |
| 218145_at | 9.88E-05 | 1.50E-06 | 5.475134 | 5.068953 | 2.030698 | TRIB3 |
| 205505_at | 7.44E-04 | 2.00E-05 | 4.721939 | 2.590624 | 2.041227 | GCNT1 |
| 224355_s_at | 3.17E-03 | 1.33E-04 | 4.147897 | 0.790841 | 2.042008 | MS4A8 |
| 212412_at | 1.00E-08 | 1.21E-11 | 8.799825 | 16.34662 | 2.045284 | PDLIM5 |
| 215432_at | 1.43E-04 | 2.39E-06 | 5.342361 | 4.624963 | 2.941853 | ACSM1 |
| 228969_at | 2.81E-04 | 5.66E-06 | 5.09299 | 3.798454 | 2.853043 | AGR2 |
| 209173_at | 1.57E-04 | 2.70E-06 | 5.306926 | 4.5069 | 3.241381 | AGR2 |
| 217113_at | 1.02E-03 | 3.06E-05 | 4.595724 | 2.18696 | 2.633504 | AMACR |
| 217111_at | 4.30E-05 | 4.97E-07 | 5.790154 | 6.131141 | 2.956508 | AMACR |
| 236365_at | 2.03E-04 | 3.72E-06 | 5.214201 | 4.198895 | 3.03945 | AMACR |
| 209426_s_at | 3.90E-06 | 2.38E-08 | 6.645619 | 9.052676 | 3.230522 | AMACR |
| 209425_at | 7.49E-07 | 2.88E-09 | 7.239489 | 11.08808 | 3.830373 | AMACR |
| 209424_s_at | 3.25E-07 | 1.02E-09 | 7.531105 | 12.0838 | 4.307398 | AMACR |
| 204416_x_at | 3.23E-04 | 6.83E-06 | 5.038361 | 3.618848 | 2.11632 | APOC1 |
| 222121_at | 8.69E-05 | 1.27E-06 | 5.521995 | 5.226226 | 2.057656 | ARHGEF26 |
| 227197_at | 4.47E-08 | 7.93E-11 | 8.257163 | 14.5402 | 2.142483 | ARHGEF26 |
| 219087_at | 7.79E-05 | 1.10E-06 | 5.564025 | 5.367523 | 2.713864 | ASPN |
| 208079_s_at | 2.24E-04 | 4.21E-06 | 5.178881 | 4.081946 | 2.223594 | AURKA |
| 230896_at | 3.13E-06 | 1.78E-08 | 6.727856 | 9.33474 | 2.26375 | BEND4 |
| 242228_at | 6.30E-04 | 1.61E-05 | 4.787066 | 2.800442 | 2.072875 | BICD1 |
| 214806_at | 4.57E-05 | 5.39E-07 | 5.766961 | 6.052577 | 2.236827 | BICD1 |
| 1556051_a_at | 1.00E-05 | 7.75E-08 | 6.314338 | 7.917448 | 2.310203 | BICD1 |
| 204741_at | 3.14E-06 | 1.79E-08 | 6.726426 | 9.329835 | 2.31932 | BICD1 |
| 213143_at | 3.21E-06 | 1.83E-08 | 6.719741 | 9.306906 | 2.224758 | C2orf72 |
| 214428_x_at | 1.88E-03 | 6.78E-05 | 4.355939 | 1.431881 | 2.305173 | C4A |
| 208451_s_at | 3.23E-03 | 1.37E-04 | 4.1407 | 0.768918 | 2.549471 | C4A |
| 221823_at | 2.85E-04 | 5.77E-06 | 5.087259 | 3.779586 | 2.049627 | C5orf30 |
| 236340_at | 1.02E-03 | 3.04E-05 | 4.597778 | 2.193495 | 2.691266 | C7orf13 |
| 210108_at | 4.72E-03 | 2.26E-04 | 3.983602 | 0.29497 | 2.081859 | CACNA1D |
| 205937_at | 2.12E-06 | 1.08E-08 | 6.868458 | 9.816984 | 2.613281 | CGREF1 |
| 214596_at | 5.43E-02 | 6.53E-03 | 2.841875 | -2.82456 | 2.162364 | CHRM3 |
| 231766_s_at | 1.83E-03 | 6.52E-05 | 4.367554 | 1.468072 | 2.383925 | COL12A1 |
| 225664_at | 2.06E-05 | 1.95E-07 | 6.05479 | 7.030758 | 2.895544 | COL12A1 |
| 217404_s_at | 4.45E-02 | 4.93E-03 | 2.945051 | -2.57051 | 2.479984 | COL2A1 |
| 213492_at | 5.32E-02 | 6.35E-03 | 2.852139 | -2.79958 | 2.516268 | COL2A1 |
| 205713_s_at | 1.52E-02 | 1.10E-03 | 3.470596 | -1.18466 | 2.055304 | COMP |
| 218002_s_at | 1.17E-02 | 7.68E-04 | 3.588858 | -0.85365 | 2.74472 | CXCL14 |
| 222484_s_at | 2.96E-03 | 1.22E-04 | 4.175158 | 0.874032 | 3.356898 | CXCL14 |
| 242138_at | 5.09E-06 | 3.35E-08 | 6.549686 | 8.723704 | 4.410439 | DLX1 |
| 232381_s_at | 4.00E-05 | 4.56E-07 | 5.814258 | 6.212842 | 2.382004 | DNAH5 |
| 219517_at | 4.90E-07 | 1.74E-09 | 7.381193 | 11.57241 | 2.191475 | ELL3 |
| 241926_s_at | 3.78E-02 | 3.88E-03 | 3.032346 | -2.35068 | 2.524717 | ERG |
| 213541_s_at | 2.32E-02 | 1.97E-03 | 3.271636 | -1.72638 | 2.936452 | ERG |
| 211626_x_at | 1.01E-02 | 6.31E-04 | 3.653545 | -0.66991 | 2.973511 | ERG |
| 231029_at | 5.35E-03 | 2.66E-04 | 3.931005 | 0.138308 | 2.433195 | F5 |
| 204713_s_at | 3.90E-03 | 1.75E-04 | 4.062644 | 0.532328 | 2.962089 | F5 |
| 204714_s_at | 1.63E-03 | 5.63E-05 | 4.41234 | 1.608004 | 3.592734 | F5 |
| 226487_at | 5.07E-05 | 6.17E-07 | 5.728558 | 5.922604 | 2.091559 | FAM222A |
| 209696_at | 1.25E-06 | 5.47E-09 | 7.058466 | 10.46833 | 2.138355 | FBP1 |
| 205776_at | 1.60E-03 | 5.50E-05 | 4.419458 | 1.630299 | 2.090409 | FMO5 |
| 227405_s_at | 1.94E-05 | 1.80E-07 | 6.077272 | 7.107418 | 2.225357 | FZD8 |
| 217445_s_at | 7.05E-08 | 1.44E-10 | 8.086129 | 13.96516 | 2.153525 | GART |
| 239761_at | 6.25E-04 | 1.59E-05 | 4.789799 | 2.809271 | 2.549322 | GCNT1 |
| 221577_x_at | 1.65E-04 | 2.89E-06 | 5.287718 | 4.442986 | 2.964905 | GDF15 |
| 229868_s_at | 4.93E-05 | 5.95E-07 | 5.738944 | 5.957741 | 3.169174 | GDF15 |
| 204973_at | 7.93E-09 | 9.28E-12 | 8.876156 | 16.59828 | 2.505874 | GJB1 |
| 1562089_at | 2.78E-05 | 2.88E-07 | 5.944461 | 6.655043 | 3.266916 | GLYATL1 |
| 227695_at | 4.50E-06 | 2.82E-08 | 6.597889 | 8.888987 | 2.459207 | GLYATL1 |
| 227794_at | 8.57E-07 | 3.45E-09 | 7.188433 | 10.91339 | 3.928912 | GLYATL1 |
| 214106_s_at | 9.06E-08 | 1.94E-10 | 8.002166 | 13.68197 | 2.318335 | GMDS |
| 218468_s_at | 3.74E-03 | 1.66E-04 | 4.079806 | 0.584163 | 2.218785 | GREM1 |
| 221582_at | 4.08E-07 | 1.38E-09 | 7.446094 | 11.79394 | 2.469479 | HIST3H2A |
| 206194_at | 4.33E-07 | 1.51E-09 | 7.42169 | 11.71066 | 2.505414 | HOXC4 |
| 206858_s_at | 4.60E-06 | 2.91E-08 | 6.589116 | 8.858904 | 3.099557 | HOXC6 |
| 204934_s_at | 7.76E-11 | 2.97E-14 | 10.59513 | 22.07723 | 2.60267 | HPN |
| 210511_s_at | 1.33E-03 | 4.30E-05 | 4.493749 | 1.863859 | 2.209158 | INHBA |
| 227140_at | 1.20E-03 | 3.76E-05 | 4.534368 | 1.99222 | 2.622898 | INHBA |
| 231993_at | 1.37E-04 | 2.27E-06 | 5.356171 | 4.671027 | 2.386657 | ITGBL1 |
| 214927_at | 2.57E-03 | 1.00E-04 | 4.235871 | 1.060205 | 2.408626 | ITGBL1 |
| 205422_s_at | 4.74E-04 | 1.11E-05 | 4.895257 | 3.151148 | 2.747703 | ITGBL1 |
| 1552897_a_at | 2.64E-02 | 2.33E-03 | 3.212406 | -1.88377 | 2.062449 | KCNG3 |
| 239319_at | 1.90E-04 | 3.44E-06 | 5.23718 | 4.275096 | 2.288701 | LINC00992 |
| 207414_s_at | 2.36E-04 | 4.53E-06 | 5.157545 | 4.011402 | 2.07582 | LOC100507472 |
| 244667_at | 2.92E-03 | 1.20E-04 | 4.181217 | 0.892558 | 3.073577 | LOC100996425 |
| 238898_at | 6.44E-05 | 8.64E-07 | 5.633043 | 5.600001 | 2.188024 | LOC101060264 |
| 211576_s_at | 2.84E-06 | 1.58E-08 | 6.760517 | 9.446769 | 2.138235 | LOC101928717 |
| 239594_at | 1.20E-04 | 1.89E-06 | 5.408785 | 4.846773 | 2.865437 | LOC145837 |
| 215323_at | 8.41E-05 | 1.21E-06 | 5.536578 | 5.275228 | 3.789194 | LUZP2 |
| 212473_s_at | 3.66E-05 | 4.09E-07 | 5.844937 | 6.316905 | 2.354535 | MICAL2 |
| 1554474_a_at | 4.73E-05 | 5.62E-07 | 5.754961 | 6.011949 | 2.208716 | MOXD1 |
| 214156_at | 3.46E-05 | 3.78E-07 | 5.86733 | 6.392918 | 2.344456 | MYRIP |
| 237168_at | 1.33E-05 | 1.10E-07 | 6.215416 | 7.579109 | 2.715461 | NEK5 |
| 213945_s_at | 4.35E-10 | 2.47E-13 | 9.949337 | 20.06373 | 2.192369 | NUP210 |
| 232482_at | 7.24E-03 | 4.02E-04 | 3.799753 | -0.24795 | 2.539651 | OR51E2 |
| 221424_s_at | 3.08E-02 | 2.90E-03 | 3.135679 | -2.0849 | 2.599548 | OR51E2 |
| 243334_at | 5.13E-03 | 2.51E-04 | 3.950422 | 0.196021 | 2.357963 | P4HA1 |
| 232575_at | 1.28E-04 | 2.08E-06 | 5.38236 | 4.758457 | 3.822882 | PCA3 |
| 232572_at | 7.89E-06 | 5.87E-08 | 6.392371 | 8.184602 | 4.558841 | PCA3 |
| 1559276_at | 3.05E-02 | 2.86E-03 | 3.140334 | -2.07279 | 2.174521 | PCAT18 |
| 216804_s_at | 4.46E-06 | 2.79E-08 | 6.601491 | 8.901339 | 2.142405 | PDLIM5 |
| 203243_s_at | 1.85E-07 | 5.04E-10 | 7.730709 | 12.76278 | 2.468702 | PDLIM5 |
| 203242_s_at | 9.81E-08 | 2.14E-10 | 7.974574 | 13.58879 | 2.842288 | PDLIM5 |
| 223551_at | 8.12E-05 | 1.16E-06 | 5.548153 | 5.314138 | 2.096671 | PKIB |
| 221088_s_at | 2.64E-06 | 1.41E-08 | 6.793699 | 9.560584 | 2.047349 | PPP1R9A |
| 206574_s_at | 5.79E-04 | 1.44E-05 | 4.819769 | 2.906174 | 2.093882 | PTP4A3 |
| 209695_at | 4.63E-05 | 5.48E-07 | 5.762056 | 6.035967 | 2.179391 | PTP4A3 |
| 227425_at | 8.56E-06 | 6.45E-08 | 6.366019 | 8.094359 | 2.188014 | REPS2 |
| 225767_at | 4.08E-07 | 1.37E-09 | 7.447833 | 11.79987 | 2.18404 | RNA45S5 |
| 201890_at | 2.62E-05 | 2.64E-07 | 5.969299 | 6.739551 | 2.707336 | RRM2 |
| 209773_s_at | 1.53E-04 | 2.62E-06 | 5.316019 | 4.537178 | 2.838372 | RRM2 |
| 204051_s_at | 1.53E-03 | 5.14E-05 | 4.439812 | 1.694132 | 2.905361 | SFRP4 |
| 206558_at | 2.60E-08 | 3.80E-11 | 8.46845 | 15.24697 | 3.518509 | SIM2 |
| 229599_at | 9.97E-03 | 6.17E-04 | 3.66054 | -0.64993 | 2.049138 | SMIM22 |
| 215806_x_at | 1.21E-04 | 1.92E-06 | 5.404178 | 4.831368 | 2.063565 | TARP |
| 209813_x_at | 7.01E-05 | 9.54E-07 | 5.604696 | 5.504452 | 2.201592 | TARP |
| 211144_x_at | 7.67E-05 | 1.07E-06 | 5.571348 | 5.392163 | 2.240111 | TARP |
| 204776_at | 1.79E-04 | 3.21E-06 | 5.256816 | 4.34028 | 2.367409 | THBS4 |
| 240303_at | 1.18E-03 | 3.66E-05 | 4.542288 | 2.017301 | 2.295923 | TMC5 |
| 229302_at | 1.64E-03 | 5.67E-05 | 4.410241 | 1.601433 | 2.62955 | TMEM178A |
| 230323_s_at | 8.59E-03 | 5.05E-04 | 3.725763 | -0.46262 | 2.424322 | TMEM45B |
| 1554101_a_at | 5.43E-05 | 6.78E-07 | 5.702024 | 5.832891 | 2.226621 | TMTC4 |
| 201291_s_at | 5.56E-05 | 7.02E-07 | 5.691831 | 5.798446 | 2.640893 | TOP2A |
| 219360_s_at | 2.24E-07 | 6.50E-10 | 7.658652 | 12.51795 | 2.647612 | TRPM4 |
| 213943_at | 6.57E-03 | 3.51E-04 | 3.843509 | -0.11994 | 2.11728 | TWIST1 |
