# Supplementary material for: Construction and analysis of mRNA, miRNA, lncRNA, and TF regulatory networks reveal the key genes associated with prostate cancer
Source: PLoS One. 2018 Aug 23;13(8):e0198055. doi: 10.1371/journal.pone.0198055 (PMC6107126; doi:10.1371/journal.pone.0198055)
Supplement: S5 Table — (DOC) [file pone.0198055.s005.doc]

**Table S5. The differentially expressed lncRNAs in GSE46602 dataset**

| ID | adj.P.Val | P.Value | t | B | logFC | Gene Symbol |
| --- | --- | --- | --- | --- | --- | --- |
| 230577_at | 5.83E-09 | 6.18E-12 | -8.99448 | 16.98711 | -4.45143 | LINC00844 |
| 231096_at | 9.56E-03 | 5.84E-04 | -3.67854 | -0.59843 | -4.0057 | PCAT4 |
| 1555942_a_at | 2.60E-08 | 3.79E-11 | -8.46927 | 15.2497 | -3.44643 | MIR205 |
| 226755_at | 4.37E-13 | 5.59E-17 | -12.6125 | 27.98743 | -3.34821 | MIR205HG |
| 239860_at | 1.83E-08 | 2.54E-11 | -8.58445 | 15.63318 | -3.19627 | LOC100130232 |
| 212732_at | 7.27E-10 | 5.05E-13 | -9.73436 | 19.38106 | -3.09182 | MEG3 |
| 238632_at | 2.14E-07 | 6.06E-10 | -7.67892 | 12.58685 | -3.01543 | RP11-44F21.5 |
| 239237_at | 3.43E-04 | 7.43E-06 | -5.0137 | 3.537954 | -3.00848 | TRG-AS1 |
| 1552463_at | 2.41E-03 | 9.28E-05 | -4.25979 | 1.13389 | -2.97481 | SERPINB11 |
| 223890_at | 3.38E-08 | 5.25E-11 | -8.37524 | 14.9357 | -2.61746 | PRO1082 |
| 216298_at | 1.17E-04 | 1.85E-06 | -5.41573 | 4.869998 | -2.5777 | TARP |
| 1558430_at | 1.49E-05 | 1.28E-07 | -6.17266 | 7.43301 | -2.57293 | RP11-680G24.5 |
| 226210_s_at | 2.77E-05 | 2.85E-07 | -5.94773 | 6.666146 | -2.41457 | MEG3 |
| 231527_at | 1.12E-03 | 3.44E-05 | -4.56092 | 2.076377 | -2.30306 | FLJ36840 |
| 1562269_at | 2.96E-07 | 9.10E-10 | -7.56379 | 12.19513 | -2.25844 | RP11-203B7.2 |
| 1556190_s_at | 1.24E-04 | 2.00E-06 | -5.39315 | 4.794514 | -2.23474 | LOC100507516 |
| 235077_at | 8.25E-10 | 6.12E-13 | -9.67708 | 19.19815 | -2.19015 | MEG3 |
| 213520_at | 4.73E-05 | 5.65E-07 | -5.75354 | 6.007121 | -2.15729 | RECQL4 |
| 1565786_x_at | 2.68E-05 | 2.73E-07 | -5.95967 | 6.706777 | -2.153 | FLJ45482 |
| 1560834_a_at | 3.59E-05 | 4.00E-07 | -5.85184 | 6.340323 | -2.14525 | RMST |
| 1558569_at | 1.66E-03 | 5.76E-05 | -4.40526 | 1.585855 | -2.09519 | LOC100131541 |
| 227390_at | 5.31E-05 | 6.59E-07 | -5.7099 | 5.859513 | -2.03453 | MEG3 |
| 227554_at | 1.29E-07 | 3.08E-10 | -7.87038 | 13.23638 | -1.94617 | MAGI2-AS3 |
| 237027_at | 4.05E-06 | 2.48E-08 | -6.63385 | 9.012299 | -1.94409 | LSAMP-AS1 |
| 241124_at | 1.76E-06 | 8.62E-09 | -6.93106 | 10.03165 | -1.94095 | LOC101928702 |
| 1560119_at | 1.63E-04 | 2.83E-06 | -5.29352 | 4.462285 | -1.93046 | LINC00937 |
| 242714_at | 1.38E-07 | 3.42E-10 | -7.84108 | 13.13716 | -1.89889 | LOC101928429 |
| 214823_at | 1.15E-04 | 1.81E-06 | -5.42182 | 4.890368 | -1.87174 | ZNF204P |
| 1567028_s_at | 5.82E-03 | 2.98E-04 | -3.8953 | 0.03255 | -1.87021 | SH3GL1P2 |
| 239466_at | 3.22E-04 | 6.79E-06 | -5.03977 | 3.623471 | -1.86947 | LINC00883 |
| 230595_at | 4.00E-02 | 4.20E-03 | -3.00334 | -2.42422 | -1.86179 | PGM5-AS1 |
| 227519_at | 1.84E-02 | 1.42E-03 | -3.38313 | -1.42522 | -1.77044 | PLAC4 |
| 1559296_at | 1.51E-07 | 3.99E-10 | -7.79681 | 12.98709 | -1.73538 | ADAMTS9-AS2 |
| 243123_at | 7.12E-05 | 9.77E-07 | -5.59782 | 5.481302 | -1.71235 | RRN3P2 |
| 236785_at | 1.90E-07 | 5.22E-10 | -7.72113 | 12.73025 | -1.70006 | LOC101928461 |
| 231882_at | 7.06E-02 | 9.77E-03 | -2.68932 | -3.18829 | -1.65688 | AP000525.9 |
| 214340_at | 4.56E-04 | 1.06E-05 | -4.90877 | 3.195129 | -1.62597 | ALOX12P2 |
| 235606_at | 5.74E-03 | 2.92E-04 | -3.90206 | 0.052551 | -1.62081 | LINC00883 |
| 231672_at | 4.38E-03 | 2.04E-04 | -4.01594 | 0.391792 | -1.59164 | LOC100653086 |
| 241614_at | 1.80E-02 | 1.38E-03 | -3.39312 | -1.39794 | -1.56974 | RP11-471B22.2 |
| 222274_at | 3.65E-02 | 3.69E-03 | -3.05004 | -2.30559 | -1.55721 | ZDHHC8P1 |
| 232034_at | 7.21E-03 | 4.00E-04 | -3.80162 | -0.24249 | -1.54411 | LINC00537 |
| 236950_s_at | 1.48E-02 | 1.06E-03 | -3.48217 | -1.15256 | -1.5063 | LINC00964 |
| 233551_at | 1.09E-03 | 3.32E-05 | -4.57193 | 2.111309 | -1.48792 | LOC642776 |
| 1561817_at | 2.20E-02 | 1.82E-03 | -3.29757 | -1.6569 | -1.48029 | AX747630 |
| 1560446_at | 8.04E-04 | 2.24E-05 | -4.68932 | 2.48591 | -1.44944 | IPO5P1 |
| 1560001_at | 8.79E-05 | 1.30E-06 | -5.51746 | 5.210985 | -1.42717 | LOC100131581 |
| 230631_s_at | 1.92E-02 | 1.50E-03 | -3.3633 | -1.47924 | -1.40391 | IL10RB-AS1 |
| 229782_at | 4.10E-02 | 4.37E-03 | -2.98952 | -2.45908 | -1.39275 | RMST |
| 215513_at | 5.77E-03 | 2.94E-04 | -3.8995 | 0.044971 | -1.37444 | HYMAI |
| 223797_at | 1.18E-02 | 7.80E-04 | -3.58372 | -0.86817 | -1.37208 | PRO2852 |
| 1561418_at | 1.48E-05 | 1.27E-07 | -6.17575 | 7.443575 | -1.35066 | LOC101928245 |
| 1559807_at | 8.10E-03 | 4.68E-04 | -3.75051 | -0.39108 | -1.34567 | BC045789 |
| 222208_s_at | 5.40E-04 | 1.32E-05 | -4.84581 | 2.990525 | -1.33809 | POLR2J4 |
| 235898_at | 9.93E-07 | 4.18E-09 | -7.13453 | 10.72885 | -1.30844 | LOC102723845 |
| 238716_at | 2.87E-03 | 1.17E-04 | -4.18778 | 0.912624 | -1.29317 | LOC100506990 |
| 232464_at | 1.11E-02 | 7.13E-04 | -3.6131 | -0.78502 | -1.27347 | TRIM78P |
| 229716_at | 2.37E-05 | 2.32E-07 | -6.00533 | 6.862207 | -1.2541 | UBXN10-AS1 |
| 208816_x_at | 6.27E-05 | 8.31E-07 | -5.64413 | 5.63738 | -1.23561 | ANXA2P2 |
| 235813_at | 1.80E-03 | 6.37E-05 | -4.3747 | 1.490363 | -1.23429 | LOC101927027 |
| 244553_at | 2.64E-03 | 1.04E-04 | -4.22339 | 1.021818 | -1.23329 | HOTTIP |
| 232300_at | 1.47E-03 | 4.90E-05 | -4.45454 | 1.740393 | -1.22922 | ADIRF-AS1 |
| 1559957_a_at | 3.39E-02 | 3.33E-03 | -3.08677 | -2.21144 | -1.20863 | LOC642852 |
| 60815_at | 1.14E-02 | 7.44E-04 | -3.59922 | -0.82435 | -1.20282 | POLR2J4 |
| 241824_at | 7.54E-03 | 4.24E-04 | -3.78247 | -0.2983 | -1.174 | RP11-373D23.2 |
| 229820_at | 4.02E-02 | 4.24E-03 | -2.99993 | -2.43284 | -1.16672 | LINC00969 |
| 240435_at | 2.06E-03 | 7.57E-05 | -4.32219 | 1.326959 | -1.16514 | LOC101928635 |
| 220701_at | 1.76E-02 | 1.33E-03 | -3.40479 | -1.366 | -1.14066 | LINC00216 |
| 227074_at | 1.01E-01 | 1.74E-02 | -2.46104 | -3.70456 | -1.13897 | LOC100131564 |
| 228215_at | 5.08E-05 | 6.20E-07 | -5.72701 | 5.917371 | -1.13393 | ADD3-AS1 |
| 232174_at | 1.34E-02 | 9.23E-04 | -3.52785 | -1.02521 | -1.12082 | AK025288 |
| 232113_at | 9.60E-02 | 1.60E-02 | -2.49666 | -3.62629 | -1.12001 | AK021804 |
| 238463_at | 1.91E-05 | 1.73E-07 | -6.0877 | 7.14298 | -1.10513 | LOC100506834 |
| 239321_at | 1.63E-02 | 1.20E-03 | -3.4395 | -1.27062 | -1.10101 | LOC441454 |
| 1559078_at | 1.26E-04 | 2.05E-06 | -5.38607 | 4.770858 | -1.09668 | AL833181 |
| 235761_at | 9.37E-02 | 1.53E-02 | -2.51377 | -3.5884 | -1.08149 | CTA-445C9.15 |
| 241873_at | 9.39E-03 | 5.72E-04 | -3.68573 | -0.57781 | -1.07698 | RP11-710C12.1 |
| 224517_at | 1.29E-02 | 8.74E-04 | -3.54587 | -0.97472 | -1.07311 | POLR2J4 |
| 230068_s_at | 9.89E-03 | 6.11E-04 | -3.66413 | -0.63967 | -1.06708 | PEG3-AS1 |
| 242465_at | 6.48E-02 | 8.54E-03 | -2.74069 | -3.06744 | -1.05517 | LOC100505592 |
| 1560109_s_at | 1.57E-01 | 3.89E-02 | -2.12204 | -4.40435 | -1.03725 | AK055458 |
| 1568647_at | 9.81E-02 | 1.66E-02 | -2.48074 | -3.66138 | -1.0322 | LOC100505851 |
| 1566968_at | 2.12E-02 | 1.72E-03 | -3.3167 | -1.60542 | -1.02409 | SPRY4-IT1 |
| 232230_at | 1.19E-01 | 2.32E-02 | -2.3434 | -3.95675 | -1.00787 | LINC00263 |
| 233599_at | 1.30E-03 | 4.16E-05 | -4.50372 | 1.895336 | -1.00704 | LOC728061 |
| 228723_at | 1.22E-02 | 8.18E-04 | -3.56797 | -0.91258 | -1.0031 | NPTN-IT1 |
| 229363_at | 1.11E-01 | 2.06E-02 | 2.392775 | -3.85208 | 1.001089 | LINC00920 |
| 228832_at | 9.14E-03 | 5.51E-04 | 3.697859 | -0.54298 | 1.010532 | FLJ20021 |
| 238623_at | 2.47E-02 | 2.13E-03 | 3.244341 | -1.79914 | 1.019724 | RP3-428L16.2 |
| 235497_at | 9.82E-02 | 1.66E-02 | 2.479816 | -3.6634 | 1.022879 | LINC01128 |
| 229899_s_at | 2.27E-02 | 1.90E-03 | 3.282663 | -1.69687 | 1.041761 | ZFAS1 |
| 228209_at | 4.64E-03 | 2.21E-04 | 3.989625 | 0.312976 | 1.063662 | LHX4-AS1 |
| 242462_at | 3.25E-04 | 6.90E-06 | 5.035418 | 3.609187 | 1.065374 | LINC00665 |
| 244749_at | 1.98E-02 | 1.56E-03 | 3.350369 | -1.51436 | 1.068471 | LOC101927204 |
| 237745_at | 1.74E-02 | 1.31E-03 | 3.410221 | -1.35111 | 1.074212 | TSC22D1-AS1 |
| 236653_at | 4.13E-02 | 4.41E-03 | 2.985956 | -2.46805 | 1.083746 | LINC00662 |
| 236585_at | 7.51E-03 | 4.22E-04 | 3.784017 | -0.29379 | 1.087355 | RP5-894A10.6 |
| 242888_at | 1.43E-01 | 3.29E-02 | 2.195711 | -4.2594 | 1.090349 | PRRT3-AS1 |
| 243362_s_at | 5.22E-03 | 2.57E-04 | 3.942167 | 0.17147 | 1.091526 | LEF1-AS1 |
| 235191_at | 1.56E-02 | 1.14E-03 | 3.458227 | -1.2189 | 1.093233 | LINC00662 |
| 238165_at | 5.77E-02 | 7.16E-03 | 2.807375 | -2.90809 | 1.094732 | PDZRN3-AS1 |
| 239450_at | 5.50E-04 | 1.35E-05 | 4.838236 | 2.965986 | 1.096009 | NDUFV2-AS1 |
| 229156_s_at | 4.53E-02 | 5.05E-03 | 2.936514 | -2.59177 | 1.10586 | PRKAG2-AS1 |
| 1559363_at | 3.45E-02 | 3.41E-03 | 3.078066 | -2.23382 | 1.112079 | LINC01146 |
| 232191_at | 1.35E-01 | 2.93E-02 | 2.244965 | -4.16023 | 1.130898 | ERVH48-1 |
| 1557383_a_at | 1.16E-01 | 2.21E-02 | 2.363452 | -3.91445 | 1.132616 | RP5-1092A3.4 |
| 231046_at | 9.06E-04 | 2.61E-05 | 4.643416 | 2.339015 | 1.13526 | LINC01023 |
| 242313_at | 1.35E-02 | 9.32E-04 | 3.524616 | -1.03427 | 1.164014 | LOC728730 |
| 227201_at | 4.49E-02 | 5.00E-03 | 2.94043 | -2.58202 | 1.199525 | LINC01128 |
| 1566557_at | 1.48E-03 | 4.96E-05 | 4.450523 | 1.727769 | 1.201015 | BAIAP2-AS1 |
| 230641_at | 1.02E-02 | 6.40E-04 | 3.648833 | -0.68336 | 1.219121 | LOC100505938 |
| 1555743_s_at | 9.91E-03 | 6.12E-04 | 3.663303 | -0.64203 | 1.251086 | ERVH-6 |
| 1559861_at | 2.20E-03 | 8.28E-05 | 4.29482 | 1.242117 | 1.275069 | LOC101928099 |
| 230302_at | 1.15E-02 | 7.49E-04 | 3.59718 | -0.83012 | 1.284279 | RP11-48B3.4 |
| 1556185_a_at | 2.02E-02 | 1.60E-03 | 3.342089 | -1.5368 | 1.293423 | CTB-167B5.2 |
| 232218_at | 6.32E-05 | 8.40E-07 | 5.641065 | 5.627057 | 1.310892 | RP11-339B21.15 |
| 229669_at | 1.52E-02 | 1.10E-03 | 3.468884 | -1.18941 | 1.312672 | LOC440416 |
| 1556639_at | 4.37E-02 | 4.79E-03 | 2.955903 | -2.54342 | 1.314545 | LOC100996455 |
| 1557598_at | 9.97E-02 | 1.71E-02 | 2.469186 | -3.68673 | 1.320978 | LOC101927870 |
| 240124_at | 7.40E-02 | 1.06E-02 | 2.659617 | -3.25741 | 1.338209 | CTA-246H3.12 |
| 227406_at | 8.11E-04 | 2.26E-05 | 4.685832 | 2.474738 | 1.367447 | GABPB1-AS1 |
| 1557599_a_at | 1.81E-02 | 1.39E-03 | 3.391223 | -1.40312 | 1.448068 | LOC101927870 |
| 229544_at | 7.89E-06 | 5.87E-08 | 6.392447 | 8.184863 | 1.489649 | RP11-391M1.4 |
| 228049_x_at | 3.84E-06 | 2.34E-08 | 6.650721 | 9.070174 | 1.540948 | SNHG19 |
| 244116_at | 1.27E-02 | 8.64E-04 | 3.549722 | -0.96391 | 1.585521 | LOC101927391 |
| 1560683_at | 6.91E-02 | 9.42E-03 | 2.703067 | -3.15612 | 1.652394 | NBEAP1 |
| 231557_at | 8.72E-03 | 5.15E-04 | 3.719511 | -0.48065 | 1.670087 | LOC102724842 |
| 1560684_x_at | 6.05E-02 | 7.67E-03 | 2.781185 | -2.971 | 1.673026 | NBEAP1 |
| 237563_s_at | 7.32E-04 | 1.96E-05 | 4.728895 | 2.612986 | 1.673082 | RP11-295G20.2 |
| 224870_at | 5.85E-04 | 1.46E-05 | 4.814884 | 2.890366 | 1.751095 | DANCR |
| 225762_x_at | 5.97E-06 | 4.12E-08 | 6.491892 | 8.525585 | 1.7786 | RNA45S5 |
| 240838_s_at | 1.68E-03 | 5.89E-05 | 4.398492 | 1.564673 | 1.780205 | LOC145837 |
| 225767_at | 4.08E-07 | 1.37E-09 | 7.447833 | 11.79987 | 2.18404 | RNA45S5 |
| 239594_at | 1.20E-04 | 1.89E-06 | 5.408785 | 4.846773 | 2.865437 | LOC145837 |
| 239319_at | 1.90E-04 | 3.44E-06 | 5.23718 | 4.275096 | 2.288701 | LINC00992 |
| 244667_at | 2.92E-03 | 1.20E-04 | 4.181217 | 0.892558 | 3.073577 | LOC100996425 |
| 238898_at | 6.44E-05 | 8.64E-07 | 5.633043 | 5.600001 | 2.188024 | LOC101060264 |
| 211576_s_at | 2.84E-06 | 1.58E-08 | 6.760517 | 9.446769 | 2.138235 | LOC101928717 |
| 239594_at | 1.20E-04 | 1.89E-06 | 5.408785 | 4.846773 | 2.865437 | LOC145837 |
| 232575_at | 1.28E-04 | 2.08E-06 | 5.38236 | 4.758457 | 3.822882 | PCA3 |
| 232572_at | 7.89E-06 | 5.87E-08 | 6.392371 | 8.184602 | 4.558841 | PCA3 |
| 1559276_at | 3.05E-02 | 2.86E-03 | 3.140334 | -2.07279 | 2.174521 | PCAT18 |
